# Pd-***η*** ^**3**^-C_6_H_9_ complexes of the Trost modular ligand: high nuclearity columnar aggregation controlled by concentration, solvent and counterion†
†Electronic supplementary information (ESI) available: Synthesis and Characterisation of compounds, and full details of aggregation studies by NMR, SANS, and MM3. See DOI: 10.1039/c5sc01181g



**DOI:** 10.1039/c5sc01181g

**Published:** 2015-07-15

**Authors:** Daugirdas Tomas Racys, Julian Eastoe, Per-Ola Norrby, Isabelle Grillo, Sarah E. Rogers, Guy C. Lloyd-Jones

**Affiliations:** a School of Chemistry , University of Bristol , Office S314, Cantock's Close , Clifton , Bristol BS8 1TS , UK . Email: Julian.Eastoe@bristol.ac.uk; b AstraZeneca Pharmaceutical Development , Global Medicines Development , Pepparedsleden 1 , SE-431 83 Mölndal , Sweden . Email: per-ola.norrby@astrazeneca.com; c Department of Chemistry and Molecular Biology , University of Gothenburg , Kemigården 4, #8076 , SE-412 96 Göteborg , Sweden; d ILL , CS20156 , 38042 Grenoble Cedex 9 , France . Email: grillo@ill.fr; e ISIS-STFC , Rutherford Appleton Laboratory , Chilton , Oxon OX11 0QX , UK . Email: sarah.rogers@stfc.ac.uk; f School of Chemistry , University of Edinburgh , Joseph Black Building, West Mains Road , Edinburgh , Scotland EH9 3JJ , UK . Email: guy.lloyd-jones@ed.ac.uk

## Abstract

Under optimised conditions, the Trost modular ligand (TML) series induces high levels of asymmetric induction in an extraordinarily wide range of reactions involving palladium *π*-allyl intermediates. Prior mechanistic investigations into reactions involving Pd-*η* ^3^-C_6_H_9_ intermediates have focussed on the monomeric 13-membered ring formed *via* P,P-chelation of the ligand to Pd. However, it is also recognised that ring-opening oligomerisation provides a pool of high nuclearity Pd-*η* ^3^-C_6_H_9_ species that, by affording a low level, or even the opposite sense, of asymmetric induction relative to the mononuclear species, are responsible for a reduction in selectivity under non-optimised conditions. Herein we describe an investigation by NMR spectroscopy, molecular mechanics, molecular dynamics, and small-angle neutron scattering (SANS), of a Pd-*η* ^3^-C_6_H_9_ cation bearing the 1,2-diaminocyclohexane TML ligand (**2**). Using both nondeuterated and perdeuterated (D_47_) isotopologues of the resulting complexes ([**1**]^+^), we show that a two-stage oligomerisation-aggregation process forms self assembled cylindrical aggregates of very high nuclearity (up to 56 Pd centres). We also investigate how concentration, solvent and counter-anion all modulate the extent of oligomerisation.

## Abstract 2

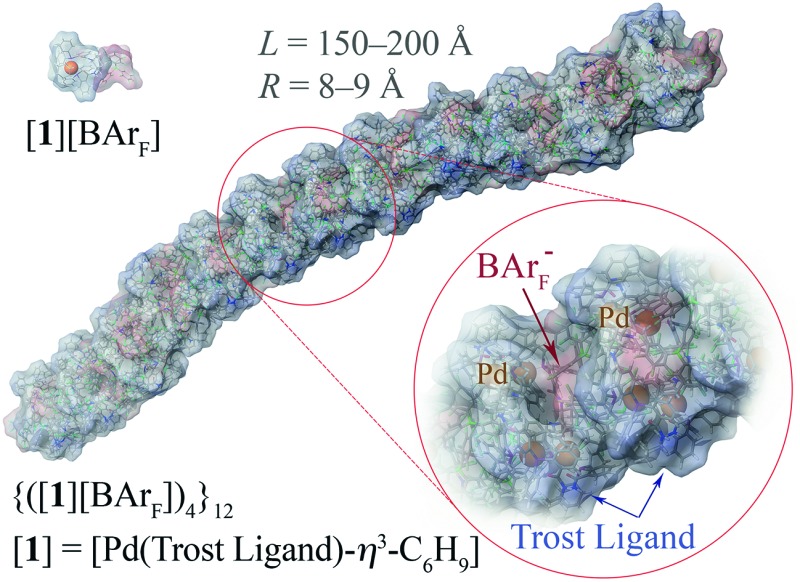
Pd-*η* ^3^-C_6_H_9_ cations bearing the Trost ligand (**2**) undergo two-stage oligomerisation-aggregation to form high nuclearity aggregates (up to 56 Pd centres), with aggregation strongly modulated by concentration, solvent and counter-anion.

## Introduction

The Trost modular ligand (TML) series^1^ has been applied to an extraordinarily wide range of allylic alkylation (Tsuji-Trost) reactions.^2^ Under carefully optimised conditions, these ligands frequently provide very high enantioselectivity in reactions that have proven challenging with other chiral ligands, particularly those involving cyclic allylic substrates.^3–6^ These features have led to broad use of the TML in the synthesis of natural products,^7^ as well as industrial application for the construction of high-enantiopurity chiral building blocks. However, reactions involving the TML can exhibit memory effects,^8,9^ and a high sensitivity of the enantioselectivity to reaction temperature, catalyst concentration, solvent and nucleophile counter-ion.
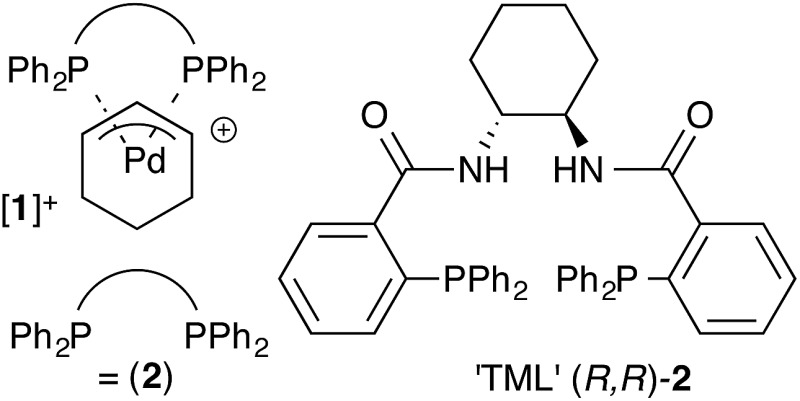


**Scheme 1:**
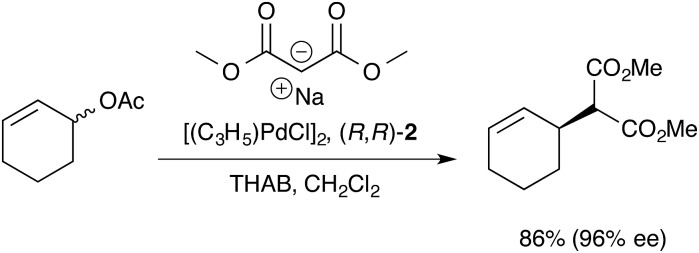
Asymmetric allylic alkylation of racemic 2-cyclohexenyl acetate; **2** = 1,2-diaminocyclohexane TML ligand, THAB = tetrahexylammonium bromide.

Our previous mechanistic studies of this system focussed on the *monomeric* cationic complex [**1**]^+^, in which a Pd(*η* ^3^-C_6_H_9_) unit is chelated by the 1,2-diaminocyclohexane-derived TML ligand (**2**).^10^ The monomeric cation [**1**]^+^ was identified as an intermediate capable of leading to high asymmetric induction on attack of, for example, a malonate anion nucleophile, Scheme 1. Detailed NMR studies facilitated by isotopic labelling – in conjunction with MM-DFT simulations – led to a model^10^ in which the amide units in the catalyst facilitate enantioselective ligand-accelerated catalysis.^11^

**Scheme 2:**
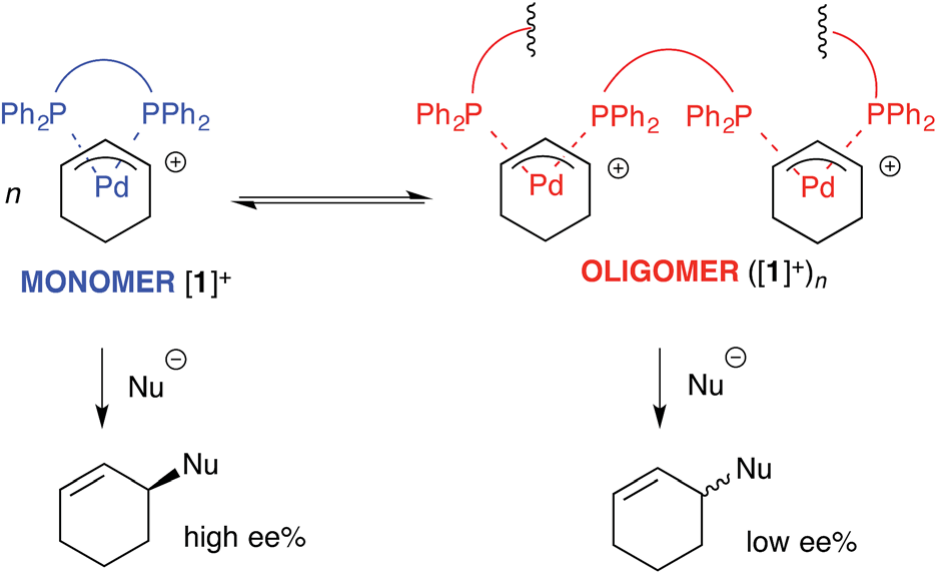
Oligomerisation of [**1**]^+^ erodes enantioselectivity during asymmetric Pd-catalysed allylic alkylation mediated by **2** (as Scheme 1). Nu = nucleophile.

For the ligand-accelerated catalysis to function efficiently, cation [**1**]^+^ requires a degree of flexibility. This flexibility is provided by the 13-membered chelate ring, but at a cost: complex [**1**]^+^ can readily undergo ring-opening oligomerisation to generate polynuclear species ([**1**]^+^)_***n***_, Scheme 2.^12^ Competing nucleophilic attack on the oligomer, rather than the monomer [**1**]^+^ is, in part, responsible for a reduction in overall enantioselectivity under non-optimised conditions.^13,14^

To date, the structure and origin of the formation of these oligomeric species has not been studied in detail. Herein, we describe an investigation of the oligomerisation of D_0_ and D_47_ isotopologues of [**1**]^+^, employing NMR spectroscopy, molecular mechanics (MM), molecular dynamics (MD), and contrast variation small-angle neutron scattering (SANS). The data obtained indicate that the impact of a first-stage of depletion of the monomeric species [**1**]^+^ from the catalyst pool, *via* cyclic oligimerisation, is amplified by a second-stage process involving columnar aggregation of the oligomers, leading to species with very high nuclearity (up to 56 Pd centres). The effects of solvent, ligand enantiopurity and counter-ion on the degree of aggregation are explored in detail, and it is concluded that a relatively small and restricted set of conditions facilitate dissolution of the complexes in a low aggregation state, consistent with the extensive optimisation frequently required for these catalyst systems.

## Results and discussion

### Preliminary NMR studies and synthesis of [D_47_]-[**1**][B(C_6_F_5_)_4_]

Despite extensive efforts,^15^ we have been unable to crystallise any Pd(*η* ^3^-C_6_H_9_) complexes of **2**, in either oligomeric or monomeric forms. Indeed, to date, the only the X-ray crystal structures of Pd-allyl complexes of ligand **2** ^16^ are *η* ^3^-C_3_H_5_ complexes with triflate counter anions: one a racemic tetranuclear cyclo-oligomer,^12^ the other an acyclic dinuclear *bis-P,O*-chelate.^17^

**Fig. 1:**
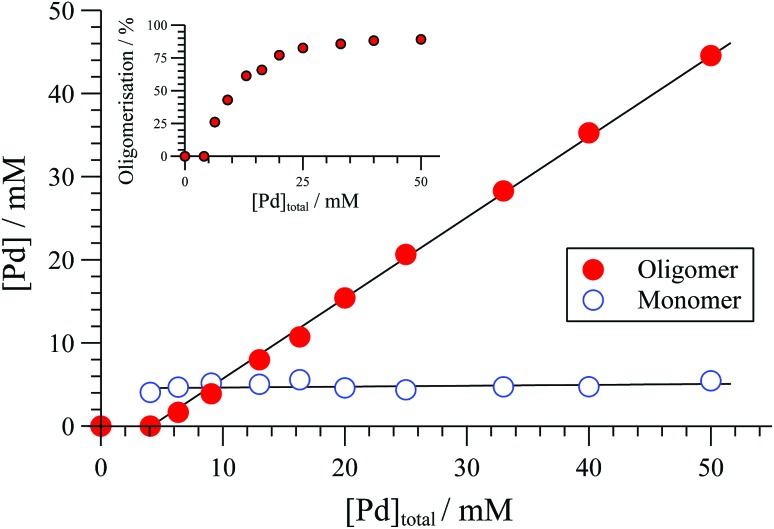
Speciation of [(*R*,*R*)-**1**][BAr_F_] (BAr_F_ = B(C_6_F_5_)_4_) determined by ^31^P{^1^H} NMR (CD_2_Cl_2_, 25 °C). Solid lines through data are solely a guide to the eye. Inset shows proportion (%) of [Pd]_tot_ that is in oligomeric form.

The extent of solution-phase oligomerisation of cationic complexes of type [**1**]^+^ can be conveniently estimated by ^31^P{^1^H} NMR spectroscopy.^10,12^ Analysis of [(*R*,*R*)-**1**][BAr_4_] complexes in CH_2_Cl_2_, where Ar = C_6_Cl_5_, 3,5-(CF_3_)_2_C_6_H_3_, or C_6_F_5_ (“BAr_F_”), indicates a maximum monomer concentration ([**1**]^+^) of about 4 mM, Fig. 1. In THF, the monomer maximum is lower (approx. 1.6 mM) and decreases as [Pd]_tot_ is raised above 10 mM. With smaller, less charge-diffuse, counter-anions such as chloride or triflate, the maximum monomer concentrations are lower still. We were unable to fit simple analytical solutions^18^ for monomer-oligomer distributions to any of the ^31^P NMR data, indicative that physicochemical effects dominate over simple solution-phase equilibria, even at low [Pd]_tot_.

**Scheme 3:**
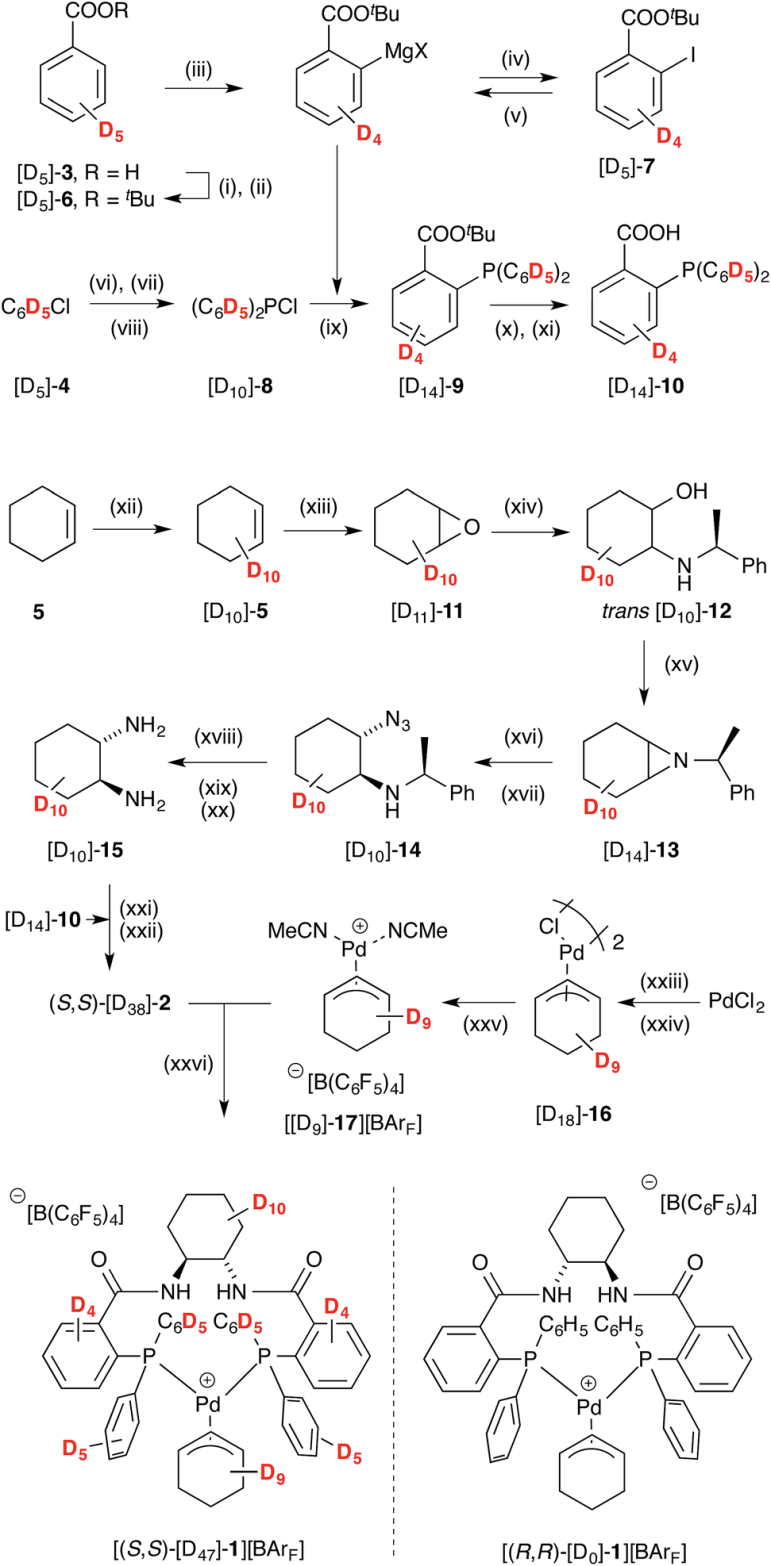
Synthesis of pseudo enantiomer [(*S*,*S*)-[D_47_]-**1**][BAr_F_]; BAr_F_ = B(C_6_F_6_)_4_. Conditions: (i) SOCl_2_, toluene, 100 °C; (ii) KO^*t*^Bu, THF 0 °C; 84%; (iii) (TMP)_2_Mg·2LiCl, THF, 0 °C to 30 °C; (iv) I_2_, THF, -30 °C; 49%; (v) ^*i*^PrMgCl, THF, -40 °C, 2 h; (vi) Mg, LiCl, THF, reflux; (vii) Et_2_NPCl_2_, THF; (viii) HCl, Et_2_O, -40 °C; 29%; (ix) [D_10_]-**8** drop-wise addition, THF, -78 °C; 68%; (x) KOH, THF, r.t., 24 h; (xi) HCl; 58%; (xii) 5% Ru(PPh_3_)_3_Cl_2_, D_2_O, 10% SDS, microwave, 140 °C, 1 h; (xiii) *m*CPBA, CH_2_Cl_2_, NaHCO_3_; 51%; (xiv) (*S*)-1-phenylethylamine, MeCN, LiBr, 60 °C; 74%; (xv) DIAD, PPh_3_, THF, 0 °C to r.t.; 62%; (xvi) NaN_3_, CeCl_3_·7H_2_O, MeCN/H_2_O 9 : 1, reflux; (xvii) silica-gel chromatography; 61%; (xviii) 20% Pd(OH)_2_/C, MeOH, r.t., 1 atm H_2_; (xix) 20% Pd(OH)_2_/C, MeOH, 2 M HCl/Et_2_O, HCO_2_NH_4_, 65 °C; (xx) KOH, CH_2_Cl_2_, r.t.; (xxi) EDCI·HCl, HOBt·H_2_O, ^*i*^Pr_2_NEt, CH_2_Cl_2_, r.t.; (xxii) Et_2_O, HCl; 30%; (xxiii) NaCl, NaOAc, CuCl_2_, AcOH, Ac_2_O, 2 h, 95 °C; (xxiv) [D_10_]-**5**, 60 °C, 20 h; 13% (xxv) KBAr_F_, MeCN, CH_2_Cl_2_, r.t.; (xxvi) CH_2_Cl_2_, r.t.; 99%. See ESI† for full details.

We thus elected to study the oligomeric species by SANS – a technique that can be used for characterising the shape and dimensions of self-assembly structure and colloids.^19^ We began with [(*R*,*R*)-**1**][BAr_F_],^20^ and, to aid the studies, also synthesised the perdeuterated enantiomeric complex [(*S*,*S*)-[D_47_]-**1**][BAr_F_]. Not only does this facilitate SANS in a non-deuterated solvent, thus providing greater neutron scattering contrast,^19^ it also allows pseudo racemic and pseudo scalemic mixtures to be prepared by mixing [(*S*,*S*)-[D_47_]-**1**][BAr_F_] with [(*R*,*R*)-**1**][BAr_F_]. The perdeuterated complex was synthesised from benzoic acid ([D_5_]-**3**), chlorobenzene ([D_5_]-**4**) and cyclohexene ([D_10_]-**5**), Scheme 3. A major hurdle was the *ortho*-metallation of ester [D_5_]-**6** with (TMP)_2_Mg·LiCl,^21^ which proceeded with an unexpectedly large net kinetic isotope effect (*k*_H_/*k*_D_ ≈ 30).^22^ This required an excess of base to be employed, and interfered with a planned direct phosphination of the metallated intermediate. Instead, the intermediate was trapped with I_2_. The iodide [D_4_]-**7** was then converted to a more conventional Grignard reagent,^23^ before reaction with chlorophosphine [D_10_]-**8** ^10,24^ to give phosphine [D_14_]-**9**, and thus acid [D_14_]-**10**.^25^

Cyclohexene [D_10_]-**5**,^26^ was epoxidised, to give [D_10_]-**11**, and this ring-opened^27^ to give aminoalcohol [D_10_]-**12**.^28^ Aziridine [D_10_]-**13**, obtained under Mitsunobu conditions, was converted to azide [D_10_]-**14**.^29^ After diastereoisomer separation, hydrogenolysis^30^ gave (*S*,*S*)-diamine [D_10_]-**15**, which was coupled with acid [D_14_]-**10** to afford Trost ligand [D_38_]-**2**. The chloro-bridged dimer [D_18_]-**16**, prepared^31^ from [D_10_]-**5**, was converted to cationic complex [D_9_]-**17**, and then reacted with [D_38_]-**2** to generate [(*S*,*S*)-[D_47_]-**1**][BAr_F_] in good yield.

### SANS analysis of aggregation of [**1**][BAr_F_] in THF

**Fig. 2:**
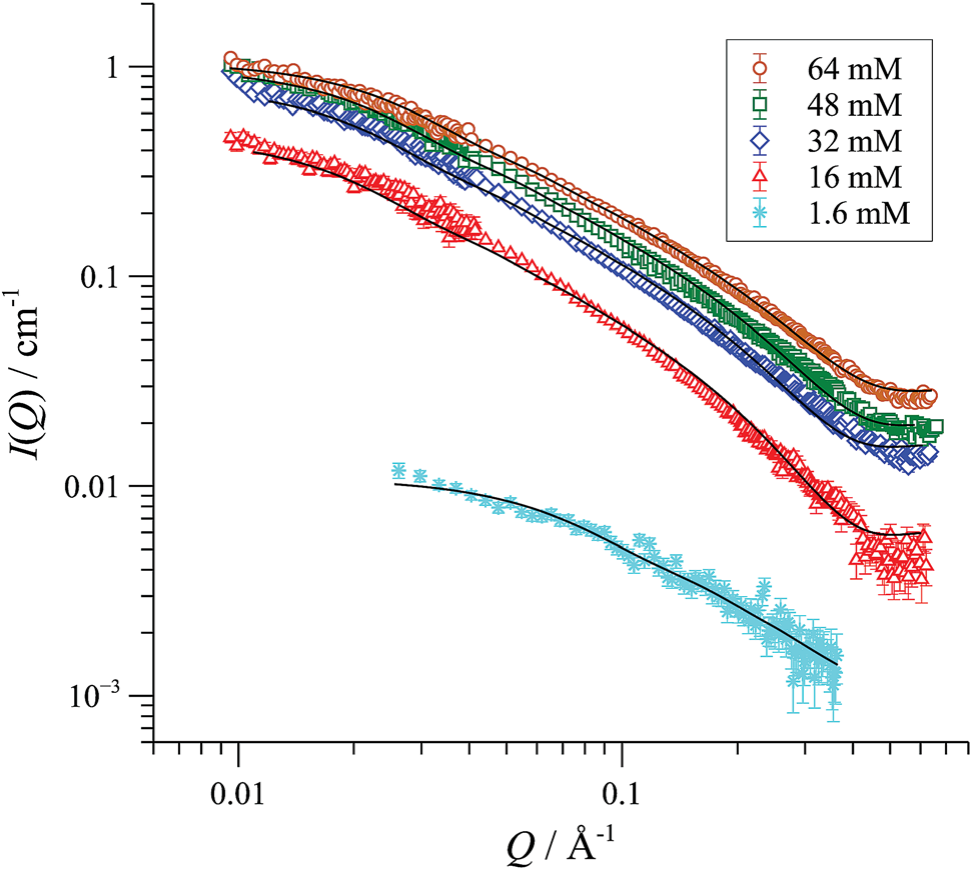
SANS profiles of 1.6 to 64 mM (0.25-10.0 wt/vol%) {[(*R*,*R*)-**1**][BArF]}_n_ in THF-D_8_, 25 °C; *I*(*Q*) = scattered intensity, *Q/*Å^–1^ = momentum transfer = (4π/λ)sin(θ/2); where scattering angle = θ and neutron wavelength = λ. The fitted curves correspond to a cylindrical particle form factor. The sample with 1.6 mM [Pd]_tot_ has form factor fitted to radius ≤ 11 Å and length ≤ 50 Å. All other samples are fitted to radius 8-9 Å and length 150-200 Å.

We began with SANS analysis^32^ of enantiopure [(*R*,*R*)-**1**][BAr_F_] in THF-D_8_ at 25 °C, with [Pd]_tot_ concentrations 16 to 64 mM, well above the oligomerisation threshold. The combined data sets,^33^ presented here as plots of scattered intensity (*I*(*Q*), *y*-axis) *versus* momentum transfer (*Q*, *x*-axis) were analysed with a range of different standard models for possible aggregate form factors, corresponding to various simple shapes,^19^ Fig. 2.^34^ This clearly identified the particles as cylindrical or rod-like, and Guinier analyses (see ESI, Fig. S69†) established the radii (8-9 Å) and lengths (150-200 Å) as essentially invariant across the range of [Pd]_tot_ explored. In other words, the number density of aggregates changes in response to [Pd]_tot_, but not their average dimensions,^35^ clearly indicative of a set of factors that tightly control the particle scale.

We have previously used ^31^P NMR spectroscopy to analyse the constitution of the solution-phase (*i.e.*, lower-order) oligomers generated from various complexes of type [**1**][BAr_4_] in CD_2_Cl_2_.^10,12^ Using PPCOSY in combination with pairs of isotopically-differentiated ligands ([D_n_]-**2**), we were able to determine that the oligomers are: i) non-chelated species (*i.e.* each of the ligands (**2**) in the oligomer are coordinated to two different Pd centres); ii) present in predominantly homochiral form (*i.e.* [(*R*,*R*)-**1**]^+^ and [(*S*,*S*)-**1**]^+^ oligomerise independently), and iii) contain no ‘free’ (*i.e.* not Pd-coordinated) P-centres in the ligand (**2**). Although a cyclic oligomer structure (Scheme 2) is fully consistent with these features, we were unable to determine the number (*n*) of ring-opened monomer units incorporated within the cyclo-oligomer ([**1**][BAr_4_])_*n*_.

As [Pd]_tot_ in THF or CH_2_Cl_2_ solutions of complexes of type [**1**][BAr_4_] is increased, the ^31^P NMR bandshape of the signals arising from the cyclo-oligomer do not change in appearance, but the samples do become increasingly turbid. This behaviour suggests that in response to an increase in [Pd]_tot_, cyclo-oligomers ([**1**][BAr_4_])_*n*_ do not incorporate more monomer (*n*), but instead aggregate to form larger particles, {([**1**][BAr_4_])_*n*_}_*m*_, containing ‘*m*’ cyclo-oligomers. It is these high nuclearity particles that are detected by SANS.^12^

**Fig. 3:**
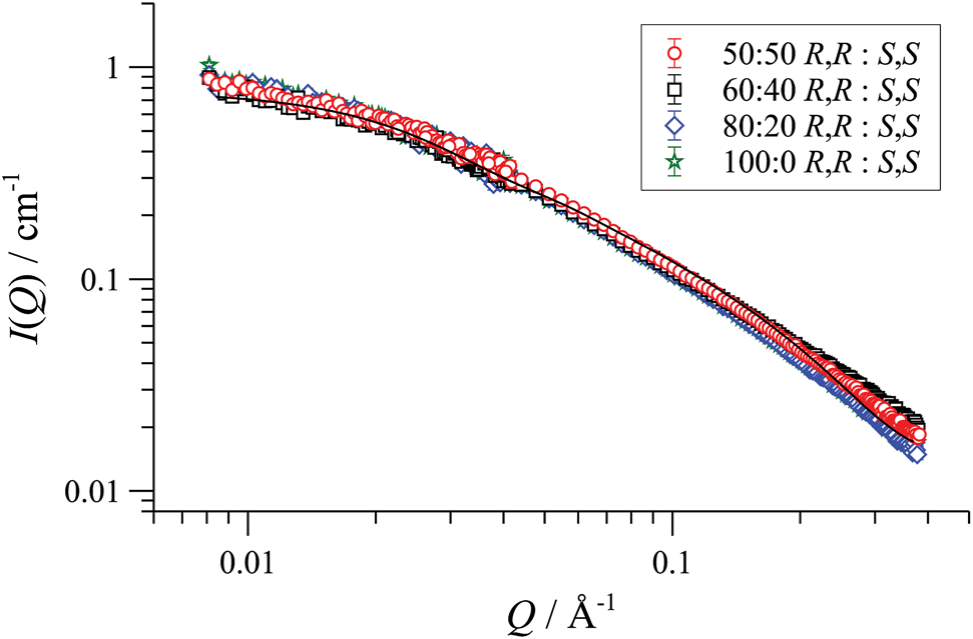
Fitted SANS profiles and cylinder form factor fits for 32 mM enantio-pure, racemic and scalemic samples of [**1**][BAr_F_] in THF-D_8_, 25 °C.

In racemic or scalemic samples of [**1**][BAr_F_], the homochiral cyclo-oligomers, ([**1**][BAr_F_])_*n*_, could aggregate in three general forms: discrete homochiral, ordered heterochiral (*e.g.*, alternating or co-block), or statistically distributed. SANS data of mixtures of [(*R*,*R*)-**1**][BAr_F_] and [(*S*,*S*)-**1**][BAr_F_] representing enantiopure, scalemic, and racemic samples, was uniform across the series, within experimental error, Fig. 3. The absence of a change in particle number density^36^ is consistent with a statistical cyclo-oligomer distribution in which there is no significant impact of homo- or hetero-chirality on the particle shape or size.

**Fig. 4:**
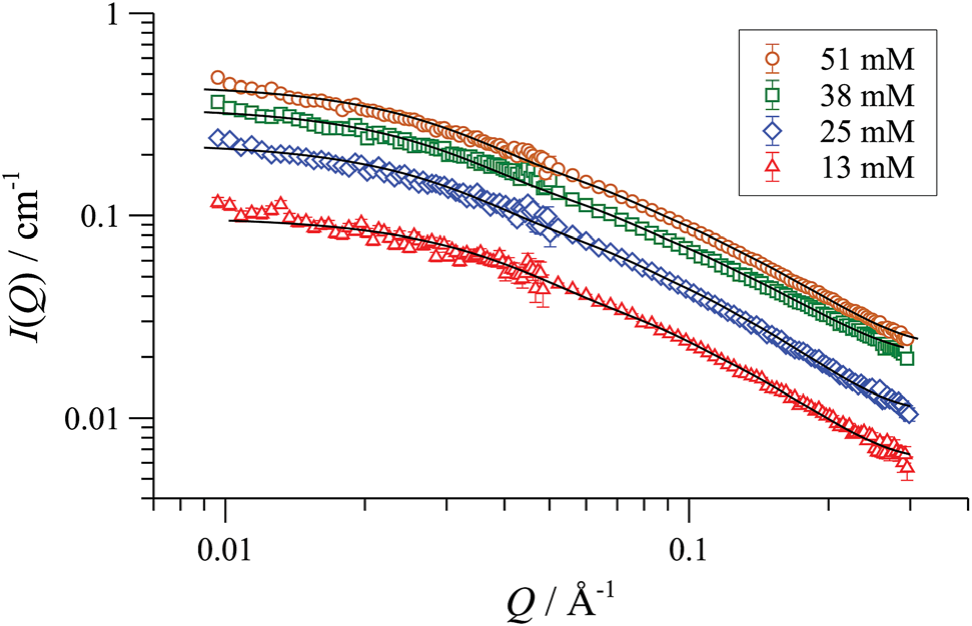
SANS data and cylinder form factor fits for pseudo racemic complex ([(*S*,*S*)-[D_47_]-**1**][BAr_F_] + [(*R*,*R*)-[D_0_]-**1**][BAr_F_]) in THF-D_8_ at 25 °C, at [Pd]_tot_ ranging from 13 to 51 mM.

SANS data of the pseudo racemate [(*S*,*S*)-[D_47_]-**1**][BAr_F_] + [(*R*,*R*)-[D_0_]-**1**][BAr_F_]) in H_8_-THF, and in D_8_-THF, Fig. 4, and enantiomerically pure [(*S*,*S*)-[D_47_]-**1**][BAr_F_] in D_0_-THF, show that the fully and partially deuterated systems retain the concentration-independent, cylindrical aggregate shape. A major difference is, however, observed in the dimensions: [D_47_]-[**1**][BAr_F_] forms shorter (130 Å), slightly wider (10 Å radius) cylinders than [D_0_]-[**1**][BAr_F_]. The pseudo racemic mixture measures as an average of its precursors (9-10 Å radius, 150 Å length), again consistent with a statistical distribution of cyclo-oligomers in the aggregate.^37^

### Molecular mechanics (MM) and dynamics (MD)

Computational modelling was employed to probe the factors that control the aggregation phenomena, and to estimate the size (*n*) and number of cyclo-oligomers in the particle (*m*).^38^ The number of atoms in the aggregates (>2,000, *vide infra*) means that density functional theory (DFT) calculations of their structures would demand currently unattainable computational resources and inordinate simulation times. On the other hand, molecular mechanics (MM) can provide a good approximation in just a small fraction of the computational time required by DFT. Although MM in general calculates only a steric energy, not the full free energy, and ignores bond dissociation energies, accurate results can be obtained from MM3 calculations provided that comparisons are isodesmic and, more importantly, isoparametric.^39^ This requirement is fulfilled for all comparisons of ring oligomers and their non-covalent aggregates. We began by confirming that the structure of the 84-atom monomeric cationic P,P-chelate [**1**]^+^, optimised at MM3 level of theory with dielectric constant *ε* = 9.0, was almost identical to that obtained using DFT (B3LYP-D3) in a polarisable continuum model for dichloromethane.^38^ Further calculations involving ion-pairs, oligomers and aggregates were then performed using MM3 to provide analysis of the energies involved, albeit at a coarse-grained level of detail.

**Fig. 5:**
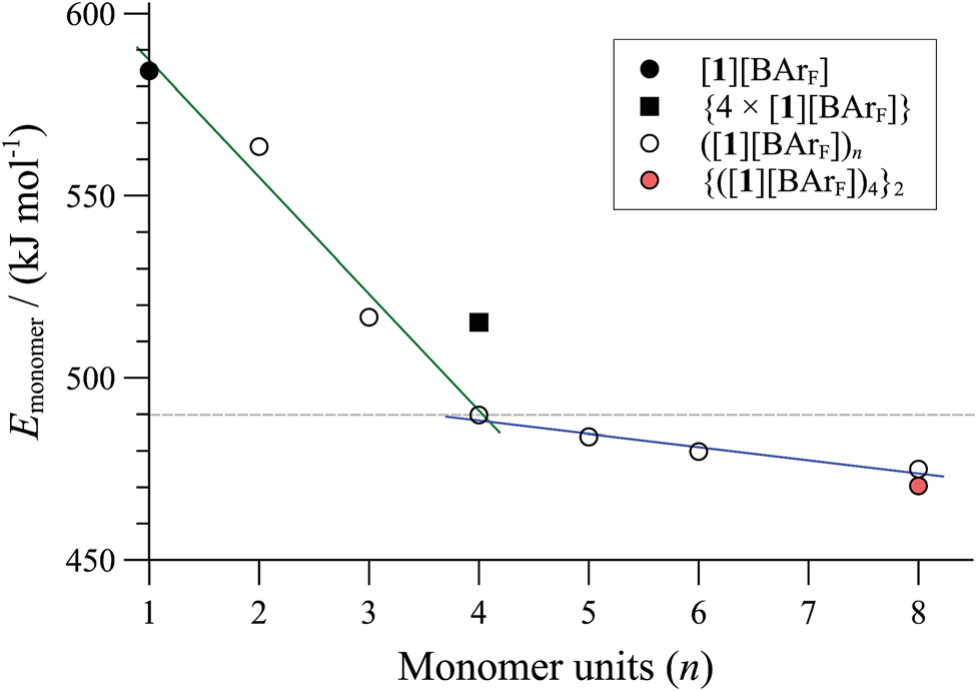
MM3 energies (*ε* = 9.0) of cyclo-oligomeric complexes ([**1**][BAr_F_])_*n*_ as a function of monomers incorporated (*n*, *x*-axis). {4 × ([**1**][BAr_F_])} is a pre-orientated assembly of four monomeric P,P-chelates; {([**1**][BAr_F_])_4_}_2_ is an aggregate formed from two tetrameric cyclo-oligomers (see *m* = 2 in Fig. 7).

Comparison of the MM3 optimised energies for monomeric P,P-chelate [**1**][BArF] with a series of homochiral cyclo- oligomers, ([**1**][BArF])_*n*_, normalised by the number of Pd atoms (*n*, the *x*-axis in Fig. 5) confirmed cyclo-oligomerisation to be exothermic, and probably also exergonic up to tetramers (*n* = 4). The cyclic dimer (*n* = 2) still suffers from ring strain, and a more substantial stability is afforded by trimerisation (*n* = 3) then tetramerisation^40^ (*n* = 4).

**Fig. 6:**
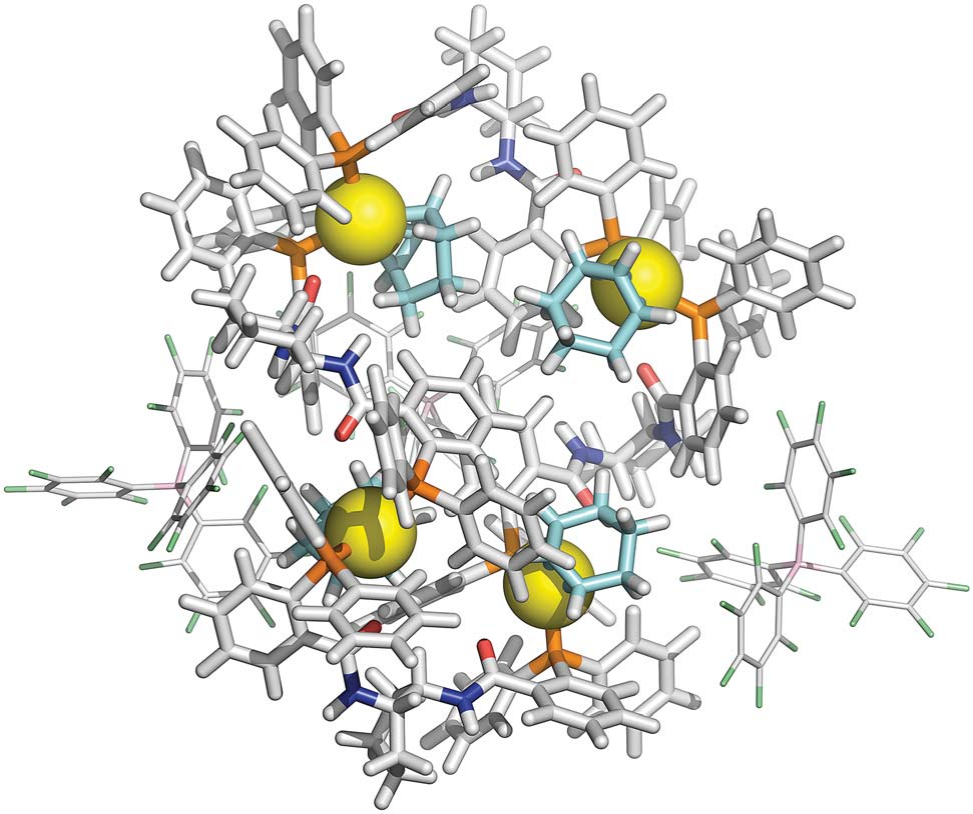
MM3 structure of tetranuclear cyclo-oligomer ([**1**][BAr_F_])_4_. For clarity, only two of the four BAr_F_ anions are shown. Colour coding: Pd, yellow; P, orange; O, red; N, dark blue; C, light grey; B, pink; F, green; *η* ^3^-C_6_H_9_, light blue.

**Fig. 7:**
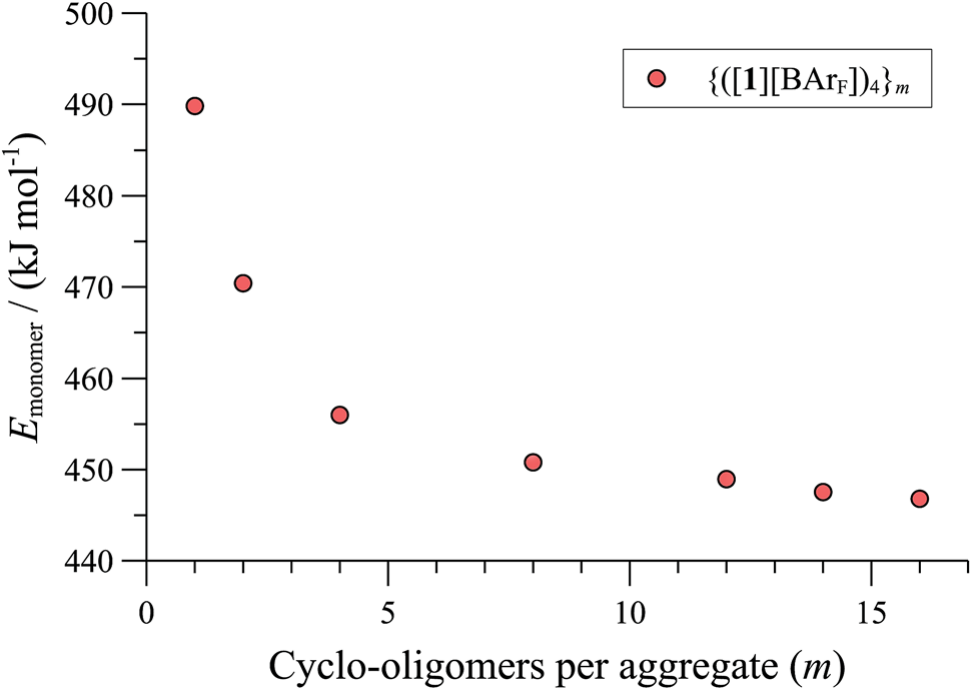
MM3 energies (*ε* = 9.0) of {([**1**][BAr_F_])_4_}_***m***_ (normalised per monomer), as a function of number (*m*) of cyclo-oligomeric tetramers aggregated.

Further increase in oligomer ring size (*n* = 5, 6, 8) yields a modest reduction in the system energy but generates species with significantly higher radii than the 8-9 Å cylinder radius detected by SANS. In summary, the tetranuclear species (*n* = 4), Fig. 6, appears to be the dominant, if not exclusive, cyclo-oligomer, and aggregation of these cyclic tetramers ([**1**][BAr_F_])_4_ was thus probed by MM3 as a process to generate the cylindrical particles, Fig. 7.

Positively charged rod-like structures with dissociated or removed anions were estimated by MM3 to be very much higher in energy than those where the anions were closely associated with the cationic cyclo-oligomeric building blocks. Anion interactions were thus explored more deeply, and although BAr_F_ is considered a weakly coordinating anion,^41^ the charge delocalisation over its surface reduces repulsive interactions with other BAr_F_ anions, and the presence of fluorine makes it significantly lipophilic. Indeed, the calculations indicated a favourable interleaving of the BAr_F_ anions in sandwich layers^42^ between cationic cyclo-oligomers. The estimated formation energies, (Δ*E*, Fig. 7) of such species {([**1**][BAr_F_])_4_}_*m*_ as a function of ‘*m*’ indicated that columnar aggregates are readily attainable, with the growing entropic cost (*T*Δ*S*) placing limits on the aggregate length.^34,40^

**Fig. 8:**
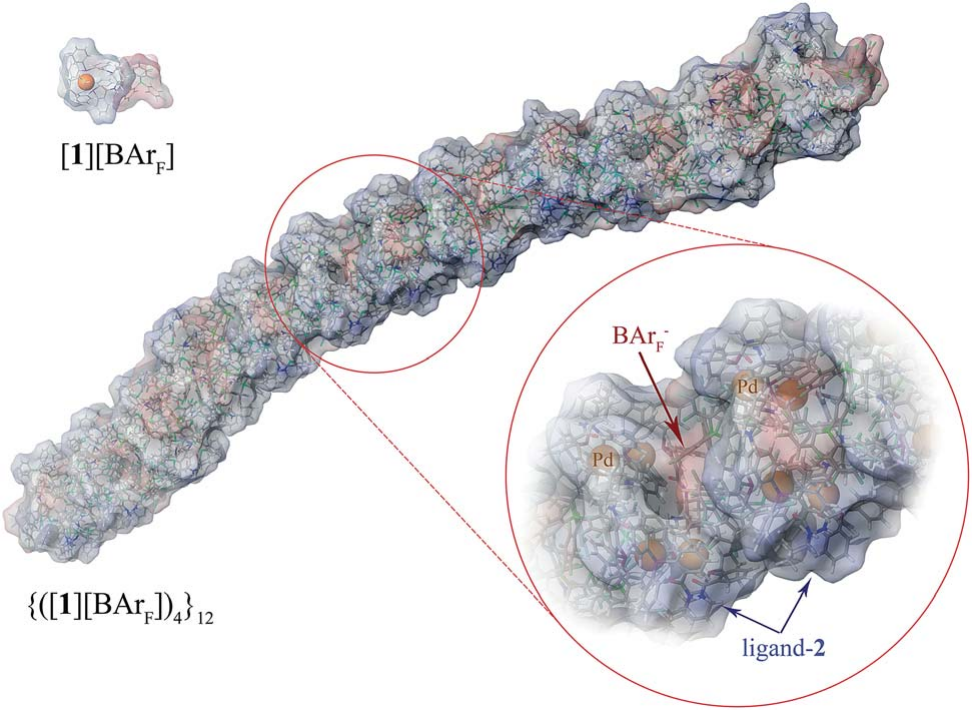
MM3 structure of {([**1**][BAr_F_])_4_}_**12**_. The monomer [**1**][BAr_F_] (top left), with palladium coloured orange, is shown for scale.

These conclusions were further probed by molecular-dynamics (MD) simulations in which the MM3-minimised structures {([**1**][BAr_F_])_4_}_*m*_ were computationally excited (300-700 K) over short periods (300 ps) to test the *relative* structural integrity of the aggregate as a function of ‘*m*’. In the low dielectric constant medium used for the model, most systems (*m* = 4 to 16) did not undergo any significant changes in their tertiary structure at 300 K. As the energy input was increased the aggregate models exhibited varying degrees of structural deformation, undergoing rapid fragmentation at the highest energies. The most significant observations were made at intermediate energies (500-550 K): aggregates with *m* = 10-14 (*e.g.*, Fig. 8, *m* = 12) retained a cylinder shape, albeit mildly distorted, over the full 300 ps simulation time, whereas higher or lower order aggregates significantly deformed, in some cases losing one or more BAr_F_ anions. The average dimensions of the MM3 aggregates with *m* = 10-14 (radius 8-9 Å and length 150-200 Å) are consistent with the particle dimensions determined by SANS, Fig. 2 and 3.

### The effect of counter-ion and solvent on shape and extent of aggregation

**Fig. 9:**
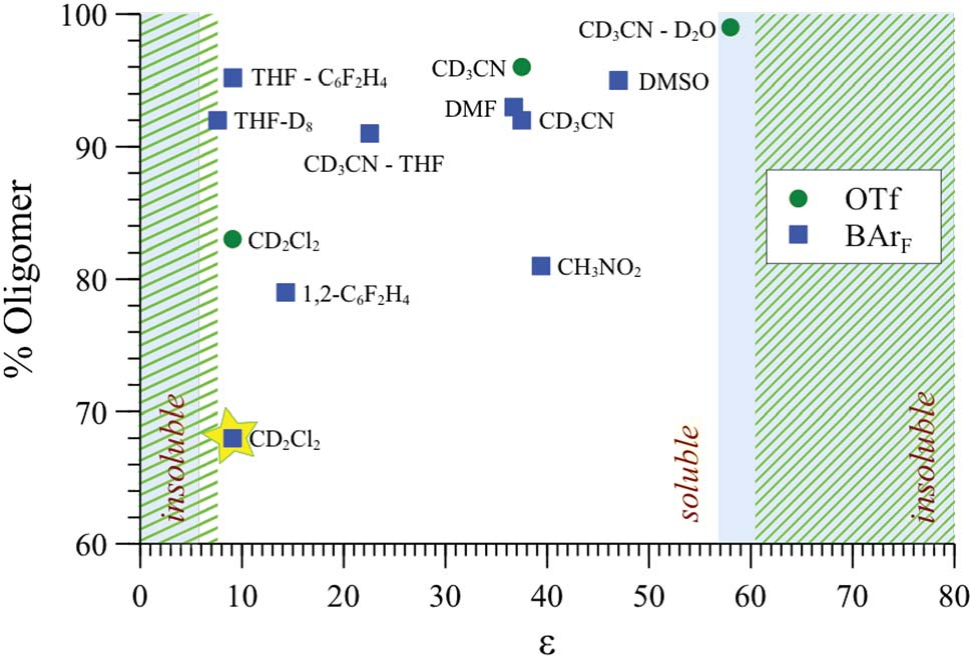
Oligomerisation (^31^P{^1^H} NMR, %) of [**1**][BAr_F_] and [**1**][OTf] at [Pd]_tot_ = 15 mM in solvents of various dielectric constant (*ε*_*r*_); overlaid areas in green (OTf) and blue (BAr_F_) indicate regions where the complexes are insoluble.

The effect of solvent type on aggregation was probed by MM, ^31^P{^1^H} NMR (Fig. 9) and SANS, also comparing [**1**][BAr_F_] with [**1**][OTf] to explore the impact of counter-ion. The aggregation mode for [**1**][BAr_F_] determined by MM3, *e.g.*, Fig. 8, involves multiple close-range electrostatic interactions that reduce the overall energy of the system. It is therefore not surprising that the solvent dielectric constant (*ε*_*r*_) was found to modulate aggregation,^43^ and thus also solubility, with precipitation being the ultimate consequence of strong aggregation.

As indicated in Fig. 9, both [**1**][OTf] and [**1**][BAr_F_] readily oligomerise and aggregate in all of the solvents that were explored, becoming essentially insoluble at the extremes of *ε*_*r*_, (*e.g.*, in alkanes, most ethers, chloroform, aromatic hydrocarbons, and at the opposite end of the scale, in water). The lipophilicity and charge-density of the anion also affects the solubility: [**1**][BAr_F_] (but not [**1**][OTf]) readily dissolves in THF, and at the opposite end of the *ε*_*r*_ scale, [**1**][OTf] (but not [**1**][BAr_F_]) is soluble in aqueous-organic mixtures.

**Fig. 10:**
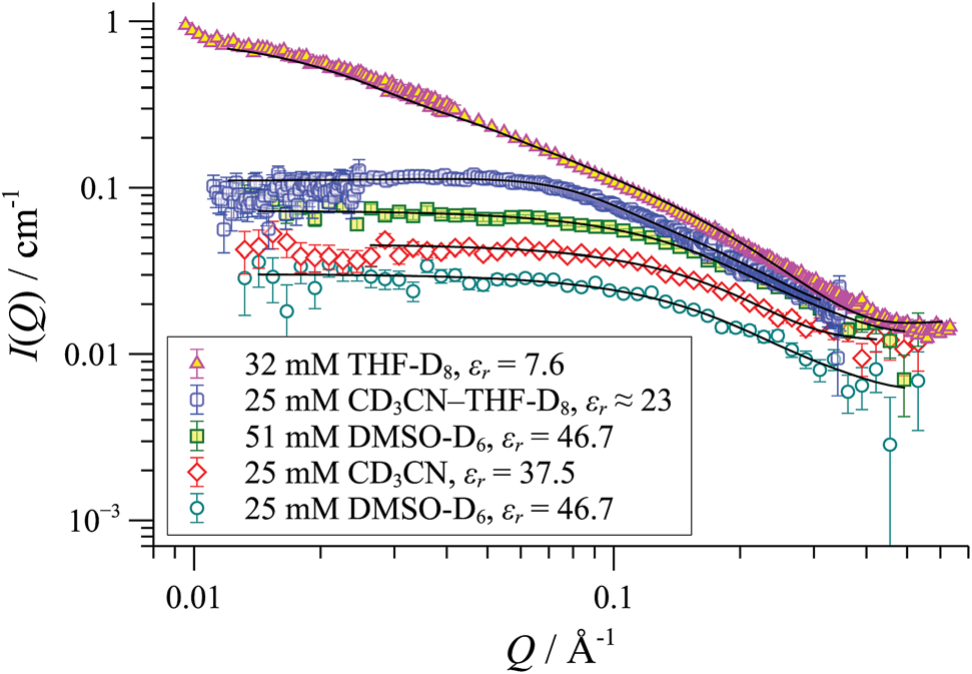
SANS data and cylinder form factor fits for [**1**][BAr_F_] in various solvents at 25 °C.

SANS was employed to explore how the macromolecular composition of aggregates {([**1**][X])_*n*_}_*m*_ is affected by solvation. Although [**1**][BAr_F_] is not soluble in organic-aqueous mixtures, SANS data were attainable in polar aprotic solvents (*e.g.*, MeCN, *ε*_*r*_ = 37.5; and DMSO, *ε*_*r*_ = 47). This confirmed that cylindrical aggregates were still formed, but were significantly shorter than those in THF, Fig. 10. Medium length cylinders were detected in a 50 : 50 mixture of THF and acetonitrile, consistent with the intermediate solvent polarity (*ε*_*r*_ ≈ 23). In all cases, the cylinders were of radius 8-10 Å, strongly suggesting the prevalence of the tetranuclear cyclo-oligomer building blocks, with the solvent modulating only the aggregation number ‘*m*’: {([**1**][BAr_F_])_4_}_*m*_.

The [**1**][OTf] aggregates behaved differently. Although, cylinders of radius 8-10 Å were again detected in all cases, indicative of {([**1**][OTf])_4_}_*m*_ aggregates, the flexibility, lengths and charge distribution in the particles were very different to those formed from [**1**][BAr_F_].

**Fig. 11:**
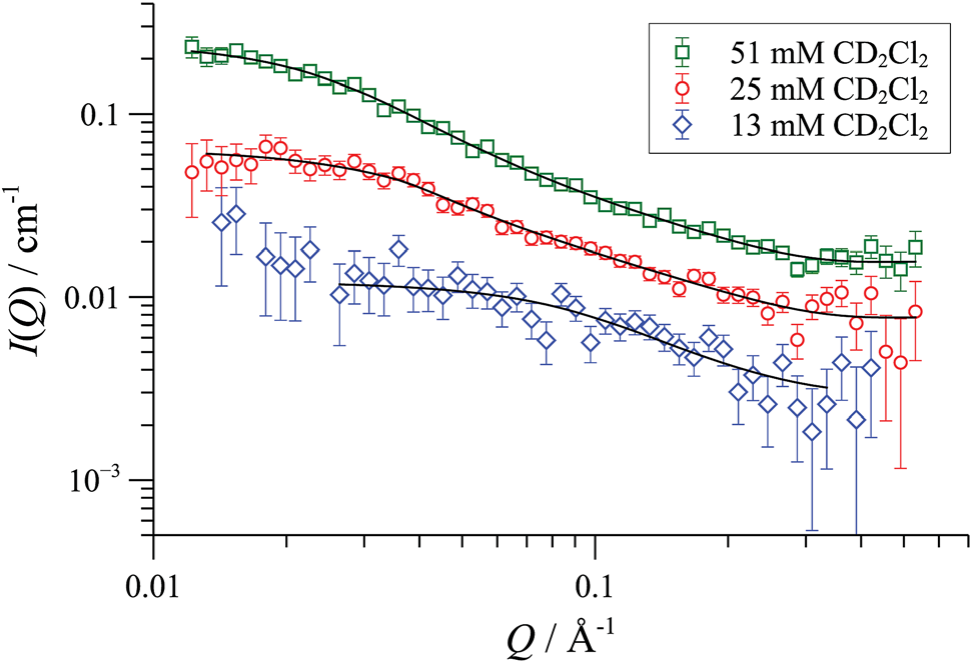
SANS data and flexible cylinder form factor fits for [**1**][OTf] in CD_2_Cl_2_ at 25 °C; fits are for radius 8-9 Å and lengths: 78 Å (13 mM); 156 Å (25 mM) and 332 Å (50 mM).

**Fig. 12:**
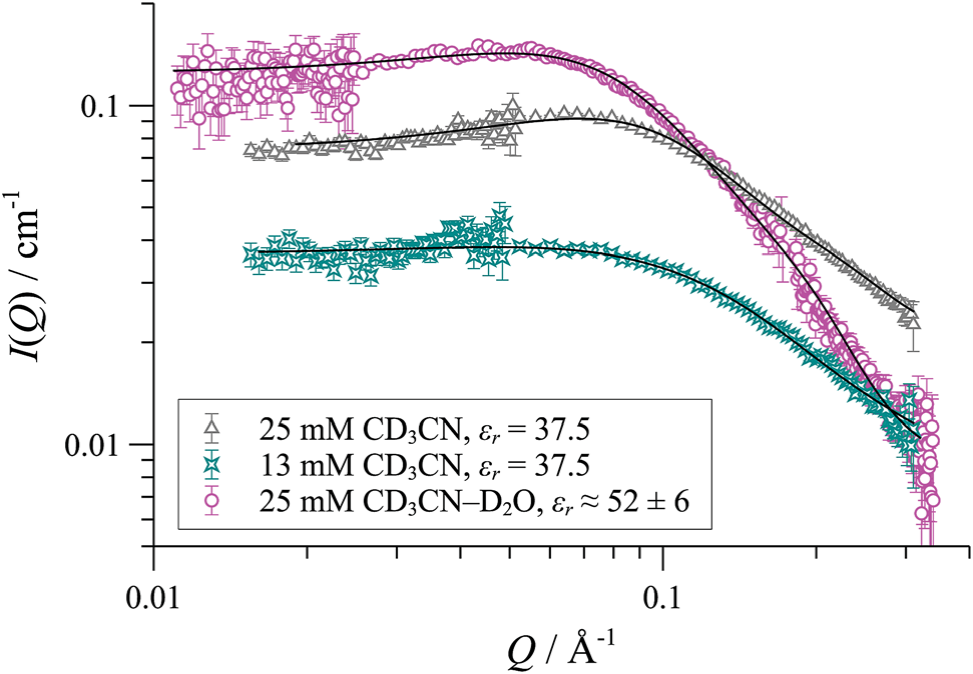
SANS data and cylinder form factor fits (including an effective Hayter-Penfold structure factor S(Q) to account for charged particles) for [**1**][OTf] in CD_3_CN at 25 °C.

In CD_2_Cl_2_, [**1**][OTf] forms cylindrical aggregates (up to 160 Å; Fig. 11) apparently with a degree of flexibility, a phenomenon that can be attributed to the small and interactive triflate anion being less able to rigidify the structures than the larger and more lipophilic BAr_F_ anion. The anion effect became even more pronounced in media of higher dielectric constant. In acetonitrile-based solvent mixtures (*ε*_*r*_ = 47-58) the SANS data indicated an additional minor structure factor contribution (S(Q)), consistent with weakly charged particles, Fig. 12. Weak repulsive interactions might arise from solvation-induced ion-pair separation of triflate from the cationic Pd(ii) oligomeric cores. The increased cationic repulsion between the cyclo-oligomers appears to result in much shorter cylinders, just 30 Å in length, with misleadingly simple ^31^P NMR spectra.^44^ Similar conclusions were drawn from MD simulations with the medium set at *ε*_*r*_ = 35: only the shortest aggregates {([**1**][OTf])_4_}_2-4_ were structurally stable at elevated energies (500 K; 300 ps). All higher aggregates (*m* > 4) underwent rapid fragmentation.

Finally, to probe the relevance of the higher aggregates to asymmetric alkylation (Scheme 1) SANS data were acquired on reaction mixtures in which [**1**][BAR_F_] was employed as a pre-catalyst (10 mol%) for addition of tetrabutylamonium dimethylmalonate to cyclohexenylacetate in THF. While the effects of substrate background scattering, varying acquisition times and shorter Q-range slightly affected the data quality, it remained clear that the dominant structures in solution, for the whole duration of the catalytic process, were large cylinders. This result is consistent with previous conclusions that, in THF, the catalytic turnover proceeds *via* a small pool of highly-active monomeric catalyst species, in competition with cyclo-oligomers and aggregates.^10,12^

## Conclusions

Since the initial report that [**1**]^+^ readily oligomerises,^13^ and that this is a largely undesirable property of an otherwise highly efficient catalyst, *e.g.*, Scheme 1, there has been limited understanding of the oligomer structures.^10,12^ We have now identified, through NMR spectroscopy, SANS, and MM/MD simulations of [[D_n_]-**1**][BAr_F_] (*n* = 0, 47),^45^ that monomeric cations [**1**]^+^ undergo chelate-opening to form a tetranuclear cyclo-oligomer ([**1**][BAr_F_])_4_; this being thermodynamically favoured over higher and lower nuclearity species. A second-stage process involving columnar-like interactions between the cyclo-oligomers then forms aggregates {([**1**][BAr_F_])_4_}_*m*_. In THF these aggregates comprise alternating layers of cyclo-oligomer and interleaved BAr_F_ anions in the form of cylindrical particles (*m* = 10-14; radius 8-9 Å; length 150-200 Å) containing up to 56 Pd(*η* ^3^-C_6_H_9_) centres, ligands (**2**) and BAr_F_ anions.

The identity of the counter-anion has a pronounced effect on the proportion of oligomer generated from the monomer. Bulky, weakly-coordinating anions,^46^ reduce the extent of oligomerisation, particularly in low polarity solvents that cannot effectively stabilise charged particles. Here the role of the bulky and relatively lipophilic anions is to solvate the monomer [**1**]^+^. Smaller harder, less lipophilic anions are less able to solvate the monomer, and have the indirect effect of shifting the equilibrium towards the oligomer; an undesirable feature for catalysis. The diminutive size of the anion also results in greater flexibility of the resulting columnar aggregates, which are more ionic in nature, reducing their solubility in less polar solvents.

**Fig. 13:**
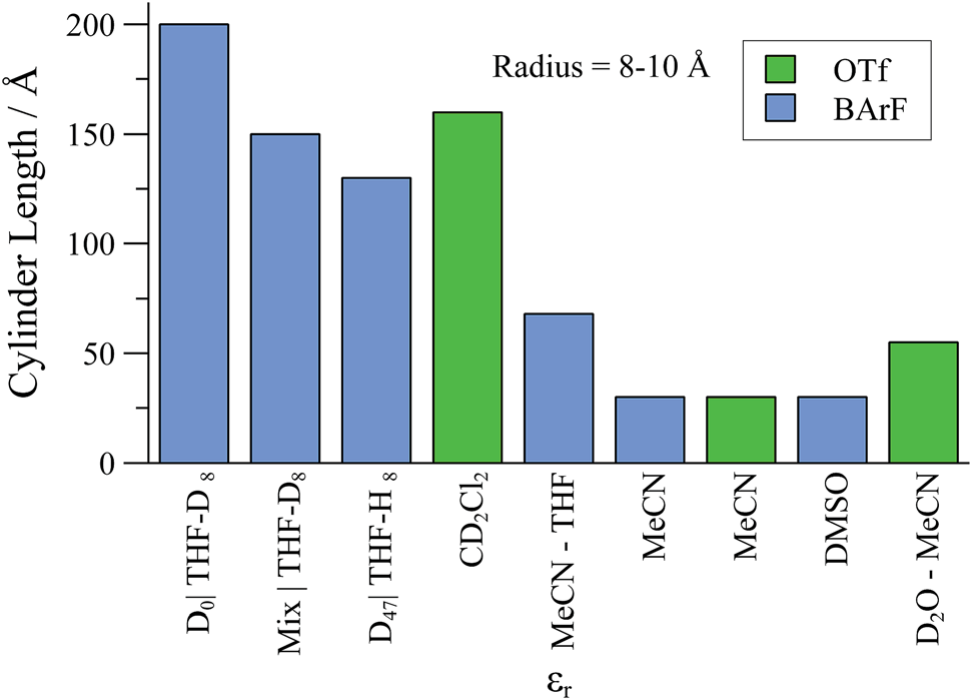
Summary of upper-range aggregate cylinder lengths of [**1**][BArF] and [**1**][OTf] samples as determined by SANS experiments, in selected solvents., with [Pd]_tot_ = 25 mM.

Overall, although the solvent polarity, counter-anion (X), and net concentration ([Pd]_tot_) all affect the degree of oligomerisation and aggregation (Fig. 13) of monomer [**1**][X], the solvent perhaps offers the greatest degree of scope for optimisation under the conditions of catalysis. In this regard, CH_2_Cl_2_ is favourable: solutions can be virtually free of oligomer at ambient temperature, provided [Pd]_tot_ ≤ 4 mM. Intriguingly, SANS studies of ionic and non-ionic surfactants^47^ have revealed specific solvent combinations that can lead to “dead zones” where aggregation is suppressed, even for concentrated solutions. If such “dead zone” solvent combinations can be found for complexes of type [**1**][X], this may be highly advantageous for improving catalytic productivity whilst maintaining selectivity.
