# Supplementary material for: Pd-η 3-C6H9 complexes of the Trost modular ligand: high nuclearity columnar aggregation controlled by concentration, solvent and counterion
Source: Chem Sci. 2015 Jul 15;6(10):5793–801. doi: 10.1039/c5sc01181g (PMC5520773; doi:10.1039/c5sc01181g)
Supplement: Supplementary file 1 [file SC-006-C5SC01181G-s001.pdf]

Electronic Supplementary Material (ESI) for Chemical Science.
This journal is © The Royal Society of Chemistry 2015

Supporting Information

S.1 GENERAL EXPERIMENTAL DETAILS 3

S.2 SYNTHESIS 4

S.2.1 Synthesis of 1,2-diaminocyclohexane-D_{10}, [D_{10}]-15 ..... *4*

Cyclohexene-D_{10}, [D_{10}]-5 4

Cyclohexene oxide D_{10}, [D_{10}]-11 5

Trans-2-((*S*-1-phenylethyl)amino)cyclohexanol-D_{10}, [D_{10}]-(*S*)-12 6

Trans-2-(((*R*)-1-phenylethyl)amino)cyclohexanol-D_{10}, [D_{10}]-(*R*)-12 7

N-(*S*-α-phenylethyl)cyclohexene-D_{10} aziridine, [D_{10}]-(*S*)-13 8

N-(*R*-α-phenylethyl)cyclohexene-D_{10} aziridine, [D_{10}]-(*R*)-13 9

S,S-2-azido-*N*-(*S*-1-phenylethyl)-D_{10}-cyclohexanamine, [D_{10}]-(*S*,S,*S'*)-14 10

R,R-2-azido-*N*-(*R*-1-phenylethyl)-D_{10}-cyclohexanamine, [D_{10}]-(*R,R,R'*)-14 11

S.2.2 Synthesis of D_{14}-2-(diphenylphosphino)benzoic acid, [D_{14}]-10 ..... *13*

Chlorodiphenylphosphine-D_{10}, [D_{10}]-8 13

Tert-butyl benzoate-D_5, [D_5]-6 14

$(TMP)_2MgCl \cdot 2LiCl$ 15

Tert-butyl 2-iodobenzoate-D_4, [D_4]-7 16

Tert-butyl D_{14}-2-(diphenylphosphino)benzoate, [D_{14}]-9 17

D_{14}-2-(diphenylphosphino)benzoic acid, [D_{14}]-10 18

S.2.3 Amide bond coupling *19*

D_{38}-*S,S*-DACH-phenyl Trost Ligand, [D_{38}]-(*S,S*)-2 19

S.2.4 Synthesis of palladium complexes, 1 ..... *20*

Bis(η^3-cyclohexenylpalladium(II)) dichloride, 16 20

Bis(η^3-D_9-cyclohexenylpalladium(II)) dichloride, [D_{18}]-16 22

[Bis-acetonitrile(η^3-cyclohexenyl)palladium(II)] tetrakis(pentafluorophenyl)borate, 17-BAr_F 23

[Bis-acetonitrile(η^3-D_9-cyclohexenyl)palladium(II)] tetrakis(pentafluorophenyl)borate, [D_9]-17-BAr_F 24

[Bis-acetonitrile(η^3-cyclohexenyl)palladium(II)] trifluoromethanesulfonate, 17-OTf 25

[(η^3-Cyclohexenyl)palladium(II){(1*R*,2*R)*-1,2-*N*-*N'*-bis[2'-(diphenylphosphino)benzoyl]
diaminocyclohexane}] tetrakis(pentafluorophenyl)borate, (*R,R*)-1-BAr_F 26

[(η^3-Cyclohexenyl)palladium(II){(1*S*,2S*)*-1,2-*N*-*N'*-bis[2'-(diphenylphosphino)benzoyl]
diaminocyclohexane}] tetrakis(pentafluorophenyl)borate, (*S,S*)-1-BAr_F 27

[(η^3-D_9-Cyclohexenyl)palladium(II){(1*S*,2*S)*-1,2-*N*-*N'*-bis[D_{14}-2'-(diphenylphosphino)
benzoyl]diaminocyclohexane-D_{10}}] tetrakis(pentafluorophenyl)borate, [D_{47}]-(*S*,S)-1-BAr_F 28

[(η^3-Cyclohexenyl)palladium(II){(1*S*,2*S)*-1,2-*N*-*N'*-bis[2'-(diphenylphosphino)benzoyl]
diaminocyclohexane}] trifluoromethylsulfonate, (*S,S*)-1-OTf 29

S.2.5 Synthesis of other compounds *30*

[(η^3-Cyclohexenyl)palladium(II){1,3-(diphenylphosphino)propane}] tetrakis(pentafluorophenyl)borate,
18-BAr_F 30

Cyclohex-2-en-1-yl acetate, 19 ... 31
S.3 NMR ... 32
S.4 SANS ... 36
S.4.1 SANS Justification ... *36*
S.4.2 Kinetic experiments ... *39*
S.5 MM3 ... 42
S.6 APPENDICES ... 44
S.6.1 NMR spectra ... *44*
S.6.2 SANS data ... *77*
S.7 REFERENCES ... 83

S.1 General experimental details

All reactions were carried out under ambient conditions unless otherwise stated in the experimental. Chemicals were purchased from Aldrich, Alfa Aesar, Fluka, Cambridge Isotope Laboratories, Chemos, Johnson Matthey, and Strem and used without further purification. The reactions were performed in oven-dried glassware under an atmosphere of nitrogen. For moisture-sensitive reactions the solvents were dried on a column packed with anhydrous alumina using equipment from Anhydrous Engineering, and deoxygenated three times following a freeze-pump-thaw procedure. Unless otherwise stated, the reactions were monitored by NMR and thin layer chromatography using glass-backed silica-gel gel 60 (F_{254}) plates. The plates were inspected under 254 nm UV light. 60 Å silica-gel gel (Merck) was used for flash column chromatography.

Instrumentation

NMR spectra were recorded on Jeol Lambda 300 (300 MHz), Jeol JNM-ECP400 (400 MHz), Varian 400-MR (400 MHz) and Varian VNMRS500a (500 MHz) at 25 ˚C. The samples were prepared in 5 mm Norell 502-7 and S500 NMR tubes; air-sensitive samples were prepared in NMR tubes fitted with a Young's valve under an atmosphere of nitrogen. Chemical shifts are reported in ppm on the *δ* scale. ^{1}H and ^{13}C chemical shifts are standardized against TMS, where δ_{TMS} = 0 ppm. Coupling constants (*J*) are measured to the nearest 0.1 Hz. The data was processed with either ACD Labs or MestreNova NMR software.

IR spectra were recorded on a Perkin-Elmer Spectrum One FT-IR spectrometer using an ATR sample accessory.

ESI^{+} **mass spectra** were recorded on Bruker Daltonics Apex IV spectrometer. EI mass spectra were recorded on *VG Autospec* instrument fitted with an EBE magnetic sector analyzer.

Melting points were measured in 1 mm capillary tubes using a *Gallenkamp Melting Point Apparatus*.

S.2 Synthesis

S.2.1 Synthesis of 1,2-diaminocyclohexane-D_{10}, [D_{10}]-15

Cyclohexene-D_{10}, [D_{10}]-5

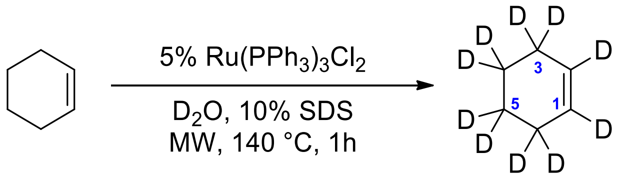

5 [D_{10}]-**5**

Four batches containing cyclohexene **5** (657 mg, 0.81 mL, 8.00 mmol, 1.0 equiv.), sodium dodecyl sulphate (SDS) (231 mg, 0.80 mmol, 0.1 equiv.) and *tris*-(triphenylphosphine)ruthenium(II) dichloride (384 mg, 0.4 mmol, 5 mol %) were mixed with D_2O (12.0 g, 10.9 mL), each in a 20 mL vials fitted with a magnetic stirrer and were sealed. The reactions were run in a microwave reactor at 140 ˚C for 60 min. The reactions were allowed to cool to room temperature. The reaction mixtures were combined, and the phases were separated, before the aqueous layer was extracted with *n*-pentane (50 mL), and the organic fractions were combined and dried over $MgSO_4$. The mixture was distilled at 42 ˚C. Cyclohexene-D_{10} [D_{10}]-**5** (1.375 g, co-isolated with residual *n*-pentane, ~9:5, estimated 40 % yield based on NMR assay) was collected at 90 ˚C. [D_{10}]-**5** was used in the next step without additional purification. **^{2}H NMR** (77 MHz, unlocked) δ 5.73 (s, 2D, C(1,2)***D***), 1.96 (s, 4D, C(3,6)***D_2***) and 1.58 (s, 4D, C(4,5)***D_2***). These data are consistent with literature.[1]

Cyclohexene oxide D_{10}, [D_{10}]-11

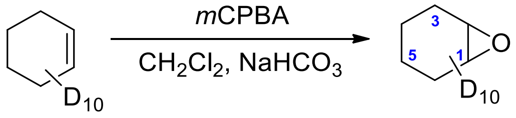

[D_{10}]-**5** [D_{10}]-**11**

Sodium hydrogen carbonate (6.44 g, 76.7 mmol, 3.4 equiv.) was heated in a dry 2-neck flask under vacuum for 5 min. The flask was cooled and refilled with nitrogen before it was charged with cyclohexene-D_{10} [D_{10}]-**5** (2.06 g, 22.3 mmol, 1.00 equiv.), and anhydrous CH_2Cl_2 (80 mL) was added *via* syringe. The reaction mixture was then cooled in an ice bath. After 15 min excess *m*CPBA (5.77 g, ≤77%, 25.7 mmol, 1.15 equiv.) in CH_2Cl_2 (60 mL) was added *via* a dropping funnel over 24 min. After stirring for 16 hours the reaction was cooled in an ice bath and carefully quenched with saturated $Na_2S_2O_3$ aq. until no peroxides could be detected with starch-iodine indicator paper. Distilled water (100 mL) was added, the mixture was extracted with diethyl ether (200 mL), and the aqueous fraction was further extracted with Et_2O (2 × 150 mL). The organic fractions were combined and washed with sat. $NaHCO_3$ aq. (100 mL), brine (100 mL) and then dried over $MgSO_4$ and concentrated *in vacuo* at 0 ºC to give the desire product [D_{10}]-**11** (1.400 g, 12.9 mmol, 58%) as clear colourless oil. [D_{10}]-**11** was used in the next step without further purification. **^{2}H NMR** (77 MHz, unlocked) δ 3.13 (s, 2D, C(1,2)***D***), 1.93 (s, 2D), 1.80 (s, 2D), 1.40 (s, 2D), 1.21 (s, 2D); **MS (EI)** *m/z* 101.07 (4%), 102.10 (4%), 103.12 (9%), 104.13 (33%) $[C_6H_4D_6O]^+$, 105.14 (73%) $[C_6H_3D_7O]^+$, 106.15 (96%) $[C_6H_2D_8O]^+$, 107.16 (100%) $[C_6HD_9O]^+$, 108.16 (51%) $[C_6D_{10}O]^+$. These data are consistent with the literature.[2]

***Trans*-2-((*S*-1-phenylethyl)amino)cyclohexanol-D_{10}, [D_{10}]-(*S*)-12**

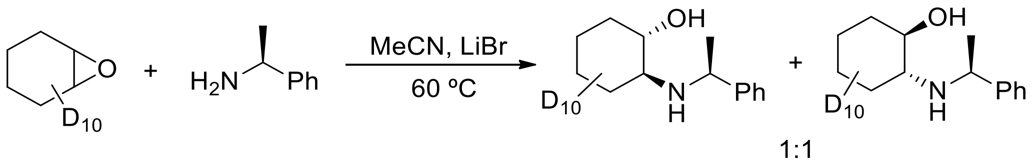

[D_{10}]-**12** [D_{10}]-(*S*)-**12**

LiBr (1.20 g, 13.8 mmol) was pre-dried in a Schlenk tube fitted with a magnetic stirrer under vacuum while heating. The Schlenk was cooled to room temperature and refilled with nitrogen, before cyclohexene oxide-D_{10} [D_{10}]-**11** (1.38 g, 12.8 mmol), anhydrous acetonitrile (5 mL) and *S*-1-phenylethylamine (1.48 g, 9.90 mmol) were added and the reaction was heated to 60 °C overnight. The reaction was confirmed to be complete by TLC analysis (Et_2O – Et_3N 40:1). The reaction mixture was concentrated *in vacuo*, then purified on silica-gel eluting with diethyl ether – triethyl amine 99:1 to give *trans*-2-(*S*-1-phenylethylamino)cyclohexanol [D_{10}]-(*S*)-**12** (2.17 g, 9.47 mmol, 74 %, mixture of *R,R,S* and *S,S,S* diastereoisomers, 98% D incorporation) as a clear colourless oil. [D_{10}]-(*R,R,S*)-**12** **^{1}H NMR** (400 MHz, $CDCl_3$) δ 7.36-7.30 (m, 4H, Ar-C(2',3',5',6')***H***), 7.29-7.22 (m, 1H, Ar-C(4')***H***), 3.92 (q, J = 6.5 Hz, 1H, -C***H***-CH_3), 3.59 (br. s, 1H, N***H***), 1.34 (d, J = 6.5 Hz, 3H, -C***H₃***) and 1.25 (br. s, 1H, O***H***); **^{13}C NMR** (126 MHz, $CDCl_3$) δ 146.75 (s, 1C, Ar-***C***(1')), 128.48 (s, 2C, Ar-***C***(3',5')H), 127.05 (s, 1C, Ar-***C***(4')H), 126.41 (s, 2C, Ar-***C***(2',6')H), 55.21 (s, 1C, -***C***H-CH_3) and 23.46 (s, 1C, -***C***H_3); **^{2}H NMR** (77 MHz, unlocked) δ 3.12 (br. s, 1D, C***D***-OH) and 2.58-0.15 (complex m, 9D, 9 × C***D***); **IR** 3344 (br., O-H), 3027 (w), 2968 (w), 2203 (m, C-D), 2105 (m, C-D), 1602 (w), 1493 (m), 1452 (m), 1369 (m), 1205 (m), 1184 (m), 1123(m), 1103 (m), 1017 (w), 951 (w), 758 (s) and 698 (s) cm^{-1}; **MS (ESI$^+$)** *m/z* 229.23 (10%) [D_9-M+H]$^+$ and 230.23 (100%) [D_{10}-M+H]$^+$. These data are consistent with that expected on the basis of data reported for the unlabelled compound.[3,4]

***Trans*-2-(((*R*)-1-phenylethyl)amino)cyclohexanol-D_{10}, [D_{10}]-(*R*)-12**

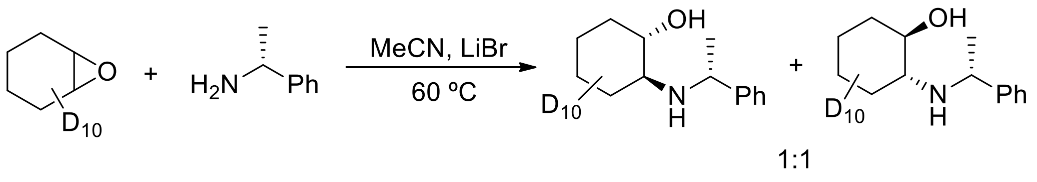

[D_{10}]-**11** [D_{10}]-(*R*)-**12**

LiBr (1.03 g, 11.9 mmol) was pre-dried in a Schlenk tube fitted with a magnetic stirrer under vacuum while heating. The Schlenk was cooled to room temperature and refilled with nitrogen, before cyclohexene oxide-D_{10} [D_{10}]-**11** (1.10 g, 10.2 mmol), anhydrous acetonitrile (4 mL) and *R*-1-phenylethylamine (1.20 g, 9.90 mmol) were added and the reaction was heated to 65 °C overnight. The reaction was confirmed to be complete by TLC analysis (Et_2O – Et_3N 40:1). The reaction mixture was concentrated *in vacuo*, then purified on silica-gel eluting with diethyl ether – triethyl amine 99:1 to give *trans*-2-(*R*-1-phenylethylamino)cyclohexanol [D_{10}]-(*R*)-**12** (1.25 g, 5.91 mmol, 59 %, mixture of *R,R,R* and *S,S,R* diastereoisomers, 90% D incorporation) as a clear colourless oil. [D_{10}]-(*S,S,R*)-**12** **^{1}H NMR** (400 MHz, $CDCl_3$) δ 7.37-7.30 (m, 4H, Ar-C(2',3',5',6')***H***), 7.29-7.22 (m, 1H, Ar-C(4')***H***), 3.92 (q, J = 6.4 Hz, 1H, -C***H***-CH_3), 3.59 (br. s, 1H, N***H***), 1.34 (d, J = 6.5 Hz, 3H, -C***H₃***) and 1.25 (br. s, 1H, O***H***); **^{13}C NMR** (126 MHz, $CDCl_3$) δ 146.77 (s, 1C, Ar-***C***(1')), 128.50 (s, 2C, Ar-***C***(3',5')H), 127.08 (s, 1C, Ar-***C***(4')H), 126.44 (s, 2C, Ar-***C***(2',6')H), 55.23 (s, 1C, -***C***H-CH_3) and 23.49 (s, 1C, -***C***H_3); **^{2}H NMR** (77 MHz, unlocked) δ 3.14 (br. s, 1D, C***D***-OH) and 2.45-0.31 (complex m, 9D, 9 × C***D***); **IR** 3397 (br., O-H), 3027 (w), 2967 (w), 2922 (w), 2203 (m, C-D), 2105 (m, C-D), 1602 (w), 1493 (m), 1452 (m), 1369 (m), 1323 (w), 1204 (m), 1183 (m), 1104 (m), 1019 (w), 952 (w), 759 (s) and 698 (s) cm^{-1}; **MS (ESI$^+$)** *m/z* 227.21 (12%) $[D_7\text{-}M+H]^+$, 228.22 (33%) $[D_8\text{-}M+H]^+$, 229.23 (85%) $[D_9\text{-}M+H]^+$ and 230.23 (100%) $[D_{10}\text{-}M+H]^+$. These data are consistent with that expected on the basis of data reported for the unlabelled compound.[3,4]

N-(S-α-phenylethyl)cyclohexene-D_{10} aziridine, [D_{10}]-(S)-13

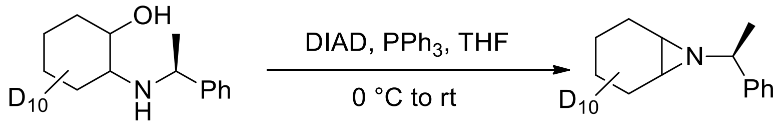

[D_{10}]-(*S*)-**12** [D_{10}]-(*S*)-**13**

A solution of *trans*-2-(((*S*)-1-phenylethyl)amino)cyclohexanol-D_{10} [D_{10}]-(*S*)-**12** (1.94 g, 8.45 mmol) in anhydrous deoxygenated THF (25 mL) was added to a dry Schlenk tube with triphenylphosphine (2.67 g, 10.1 mmol) under an atmosphere of nitrogen. The mixture was cooled in an ice bath and DIAD (2.0 mL, 2.0 g, 10 mmol) was added dropwise *via* syringe, and was left to warm to room temperature. After 2 days, a small aliquot of DIAD and PPh_3 was added to drive the reaction to the completion. The volatile organic compounds were removed *in vacuo* by azeotroping the mixture with toluene. The orange gum was extracted three times with *n*-pentane (3 × 30 mL) by sonication and mechanical stirring. The solid was filtered off and the filtrate was concentrated *in vacuo* to give crude [D_{10}]-(*S*)-**13** as yellow oil. The residue [D_{10}]-(*S*)-**13** was purified on silica-gel (*n*-hexane – ethyl acetate 100:0 to 96:4) to give an almost colourless clear oil [D_{10}]-(*S*)-**13** (1.10 g, 5.21 mmol, 62%, 98% D incorporation). **^{1}H NMR** (400 MHz, $CHCl_3$) δ 7.44-7.39 (m, 2H, Ar-C(2’,6’)***H***), 7.36-7.30 (m, 2H, Ar-C(3’,5’)***H***), 7.26-7.21 (m, 1H, Ar-C(4’)***H***), 2.46 (d, J = 6.6 Hz, 1H, -C***H***-CH_3) and 1.39 (d, J = 6.6 Hz, 3H, -C***H$_3$***); **^{13}C NMR** (101 MHz, $CDCl_3$) δ 145.70 (s, 1C, Ar-***C***(1’)H), 128.05 (s, 2C, Ar-***C***(3’,5’)H), 126.63 (s, 2C, Ar-***C***(2’,6’)H), 126.43 (s, 1C, Ar-***C***(4’)H), 69.69 (s, 1C, -***C***H-CH_3) and 23.58 (s, 1C, -***C***H_3); **^{2}H NMR** (77 MHz, unlocked) δ 1.85 (br. s, 2D), 1.66 (br. s, 3D), 1.50 (br. s, 1D), 1.41 (br. s, 2D), 1.18 (br. s, 2D); **IR** 2966 (w), 2820 (w), 2210 (m, C-D), 2097 (m, C-D), 1492 (m), 1448 (m), 1365 (m), 1347 (m), 1136 (m), 993 (m), 961 (m), 757 (m), 698 (s) and 667 (m) cm^{-1}; **MS (ESI$^+$)** *m/z* 212.22 $[M+H]^+$. These data are consistent with that expected on the basis of data reported for the unlabelled compound.[5,6]

***N*-(*R*-α-phenylethyl)cyclohexene-D_{10} aziridine, [D_{10}]-(*R*)-13**

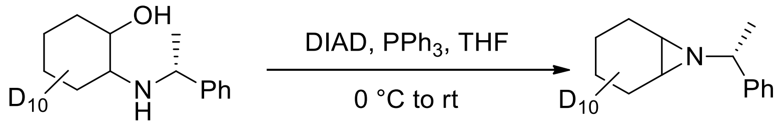

[D_{10}]-(*R*)-**12** [D_{10}]-(*R*)-**13**

A solution of *trans*-2-(((*R*)-1-phenylethyl)amino)cyclohexanol-D_{10} [D_{10}]-(*R*)-**12** (1.24 g, 5.41 mmol) in anhydrous deoxygenated THF (10 mL + 5 mL wash) was added to a dry Schlenk tube with triphenylphosphine (1.70 g, 6.48 mmol) under an atmosphere of nitrogen. The mixture was cooled in an ice bath and DIAD (1.3 mL, 1.3 g, 6.5 mmol) was added dropwise *via* syringe, and was left to warm to room temperature overnight. The volatile organic compounds were removed *in vacuo* by azeotroping the mixture with toluene. The orange gum was extracted three times with *n*-pentane (3 × 30 mL) by sonication and mechanic stirring. The solid was filtered off and the filtrate was concentrated *in vacuo* to give crude [D_{10}]-(*R*)-**13** as orange oil. The residue [D_{10}]-(*R*)-**13** was purified on silica-gel (*n*-hexane – ethyl acetate 100:0 to 96:4) to give colourless clear oil [D_{10}]-(*R*)-**13** (588 mg, 2.78 mmol, 52%, 88% D incorporation). **^{1}H NMR** (301 MHz, $CDCl_3$) δ 7.43-7.37 (m, 2H, Ar-C(2,6)***H***), 7.36-7.28 (m, 2H, Ar-C(3',5')***H***), 7.27-7.19 (m, 1H, Ar-C(4')***H***), 2.45 (q, J = 6.3 Hz, 1H, -C***H***-CH_3) and 1.38 (dd, J = 6.6, 1.5 Hz, 3H, -C***H₃***); **^{13}C NMR** (101 MHz, $CDCl_3$) δ 128.06 (s, 2C, Ar-***C***(3',5')H), 126.63 (s, 2C, Ar-***C***(2',6')H), 126.43 (s, 1C, Ar-***C***(4')H), 69.69 (s, 1C, -***C***H-CH_3) and 23.57 (s, 1C, -***C***H_3); **^{2}H NMR** (46 MHz, unlocked) δ 1.88 (br. s, 2D), 1.74 (br. s, 1D), 1.64 (br. s, 2D), 1.51 (br. s, 1D), 1.43 (br. s, 2D) and 1.18 (br. s, 2D). These data are consistent with that expected on the basis of data reported for the unlabelled compound.[5,6]

***S,S*-2-azido-*N*-(*S*-1-phenylethyl)-D_{10}-cyclohexanamine, [D_{10}]-(*S,S,S'*)-14**

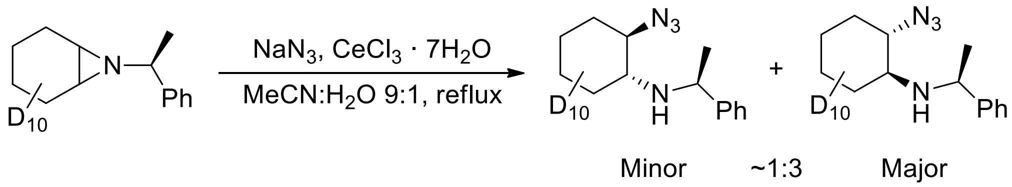

[D_{10}]-(*S*)-**13** [D_{10}]-(*R,R,S'*)-**14** [D_{10}]-(*S,S,S'*)-**14**

N-(*S*-α-phenylethyl)-D_{10}-cyclohexene aziridine **[D_{10}]-(*S*)-13** (1.030 g, 4.874 mmol, 1.0 equiv.), $CeCl_3 \cdot 7H_2O$ (907 mg, 2.44 mmol, 0.50 equiv.) and sodium azide (306 mg, 4.73 mmol, 0.97 equiv.) were dissolved in acetonitrile water (9 : 1) mixture (10 mL) in a round bottomed flask fitted with a magnetic follower and a reflux condenser. The reaction was heated to 90 °C for 80 min. The reaction was allowed to cool to room temperature, and was confirmed to be complete by TLC analysis (*n*-pentane – ethyl acetate 30:1). The reaction mixture was diluted with distilled water (70 mL), and extracted with dichloromethane (3 × 50 mL). The organic fractions were combined, washed with distilled water (20 mL), dried over $MgSO_4$ and concentrated *in vacuo* to crude oil [D_{10}]-(*S'*)-**14**. The oil [D_{10}]-(*S'*)-**14** was purified and diastereomers separated on deactivated silica-gel (*n*-hexane – ethyl acetate 30:1 with 2.5% triethylamine) eluting with *n*-hexane – ethyl acetate 30:1 to give [D_{10}]-(*S,S,S'*)-**14** (749 mg, 2.94 mmol, 64%) as clear colourless oil. **^{1}H NMR** (400 MHz, $CDCl_3$) δ 7.29–7.12 (m, 5H, Ar-***H***), 3.86 (q, $^3J_{HH}$ = 6.6 Hz, 1H, -C***H***-CH_3), 1.37 (br. s, 1H, N-***H***), 1.29 (d, $^3J_{HH}$= 6.6 Hz, 3H, -C***H₃***); **^{2}H NMR** (77 MHz, unlocked) *δ* 3.10 (s, 1D), 2.45 (s, 1D), 2.04 (s, 1D), 1.77 (s, 1D), 1.73 (s, 1D), 1.59 (s, 1D), 1.38 (s, 1D), 1.26 (s, 1D), 1.15 (s, 1D) and 0.97 (s, 1D); **^{13}C NMR** (101 MHz, $CDCl_3$) *δ* 146.94 (s, 1C, Ar-***C***(1')-), 128.35 (s, 2C, Ar-***C***(3',5')H), 126.74 (s, 1C, Ar-***C***(4')H), 126.46 (s, 2C, Ar-***C***(2',6')H), 56.58 (s, 1C, -***C***H-CH_3) and 24.29 (s, 1C, -***C***H_3); **IR** 2962 (w), 2206 (w), 2093 (s, -N_3), 1492 (w), 1465 (m), 1451 (m), 1369 (w), 1351 (w), 1271 (m), 1255 (m), 1199 (w), 1180 (w), 1129 (m), 1067 (w), 1031 (m), 1020 (w), 1004 (m), 760 (s) and 698 (s) cm^{-1}; **MS (ESI$^+$)** *m/z* 254.23 (10%) $[D_9\text{-}M+H]^+$, 255.24 (100%) $[D_{10}\text{-}M+H]^+$ and 277.22 $[D_{10}\text{-}M+Na]^+$. These data are consistent with that expected on the basis of data reported for the unlabelled compound.[5, 7]

***R,R*-2-azido-*N*-(*R*-1-phenylethyl)-D_{10}-cyclohexanamine, [D_{10}]-(*R,R,R'*)-14**

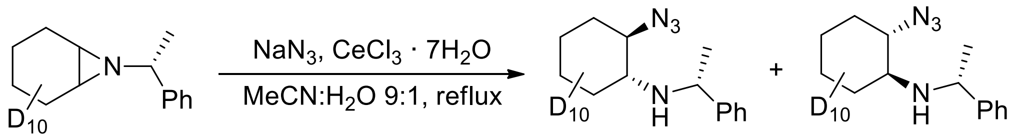

Major ~3:1 Minor

[D_{10}]-(*R*)-**13** [D_{10}]-(*R,R,R'*)-**14** [D_{10}]-(*S,S,R'*)-**14**

N-(*R*-α-phenylethyl)-D_{10}-cyclohexene aziridine [D_{10}]-(*R*)-**13** (566 mg, 2.68 mmol, 1.0 equiv.), $CeCl_3 \cdot 7H_2O$ (500 mg, 1.34 mmol, 0.50 equiv.) and sodium azide (171 mg, 2.60 mmol, 0.97 equiv.) were dissolved in acetonitrile water (9 : 1) mixture (5 mL) in a round bottomed flask fitted with a magnetic follower and a reflux condenser. The reaction was heated to 90 °C for 90 min. The reaction was allowed to cool to room temperature and was diluted with distilled water (30 mL), and extracted with dichloromethane (70 and 2 × 30 mL). The organic fractions were combined, washed with distilled water (20 mL), dried over $MgSO_4$ and concentrated *in vacuo* to crude oil [D_{10}]-(*R'*)-**14**. The oil [D_{10}]-(*R'*)-**14** was purified and diastereomers separated on deactivated silica-gel (*n*-pentane – ethyl acetate 30:1 with 2.5% triethylamine) eluting with *n*-pentane – ethyl acetate 30:1 to 4:1 to give [D_{10}]-(*R,R,R'*)-**14** (361 mg, 1.42 mmol, 53%) as clear colourless oil, [D_{10}]-(*S,S,R'*)-**14** (83 mg, 0.33 mmol, 12%) and mixed fractions (126 mg, 0.496 mmol, 19%). [D_{10}]-(*R,R,R'*)-**14**: **^{1}H** and **^{2}H NMR** data are identical to [D_{10}]-(*S*,S,*S'*)-**46**; **CHN** found: C, 66.176; H, 8.029; N, 21.731. [D_{10}]-(*S,S,R'*)-**14**: **^{2}H NMR** (77 MHz, unlocked) δ 3.10 (s, 1D), 2.28 (s, 1D), 2.13 (s, 1D), 2.05 (s, 1D), 1.76 (s, 1D), 1.68 (s, 1D), 1.34 (s, 2D), 1.13 (s, 1D) and 1.10 (s, 1D). These data are consistent with that expected on the basis of data reported for the unlabelled compounds.[5,7]

(1*S*,2*S*)-diaminocyclohexane-[D_{10}], [D_{10}]-(*S*,*S*)-15

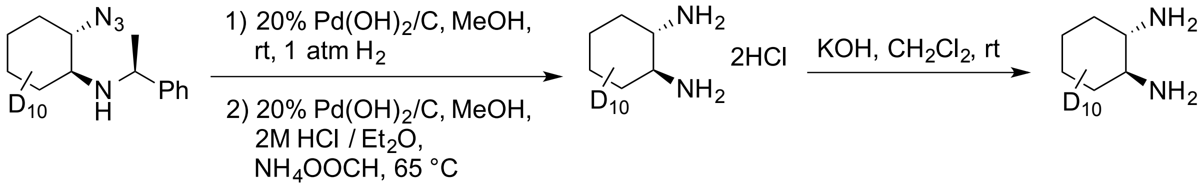

[D_{10}]-(*S*,*S*,*S'*)-**14**

[D_{10}]-(*S*,*S*)-**15**

S,*S*-2-azido-*N*-(*S*-1-phenylethyl)-D_{10}-cyclohexanamine [D_{10}]-(*S*,*S*,*S'*)-**14** (370 mg, 1.46 mmol, 1.00 equiv.) and $Pd(OH)_2$/C (90 mg, 20% loading) were mixed in anhydrous deoxygenated methanol (4 mL). The reaction mixture was purged with hydrogen three times and stirred at 1.0 bar (balloon) hydrogen atmosphere for 20 h at room temperature. The reaction mixture was purged with nitrogen, before HCl in diethyl ether (0.8 mL, 2M) and ammonium formate (282 mg, 4.48 mmol, 3.07 equiv.) were added. The reaction was slowly heated to 76 °C for 150 min. The reaction mixture was cooled to room temperature, acidified with excess HCl in Et_2O (2-3 mL, 2M), diluted with anhydrous methanol (~5 mL) and filtered through a cotton plug. The solvent was removed *in vacuo* to give a white solid. [65 mg sample was removed.] The transfer hydrogenation step was repeated with extra $Pd(OH)_2$/C (60 mg, 20% loading) and ammonium formate (150 mg) in methanol (5 mL) to ensure complete conversion.

The crude salt was mixed with freshly ground KOH (212 mg) in CH_2Cl_2 (4 mL) and stirred for 4 h at room temperature under atmosphere of nitrogen. The reaction mixture was filtered through a cannula and concentrated under reduced pressure (1 torr, rt). The residue was re-dissolved in dichloromethane and concentrated *in vacuo* to an oil [D_{10}]-(*S*,*S*)-**15** (0.252 mmol according to NMR assay, 17%) and was stored under nitrogen atmosphere to prevent degradation. **^{2}H NMR** (77 MHz, unlocked) δ 2.37 (s, 2D, 2 × C***D***-NH_2), 1.89 (s, 2D, -C$\boldsymbol{D_2}$-), 1.70 (s, 2D, -C$\boldsymbol{D_2}$-), 1.25 (s, 2D, -C$\boldsymbol{D_2}$-) and 1.18 (s, 2D, -C$\boldsymbol{D_2}$-); **MS (EI)** *m/z* 120.1 (100%) $[D_6\text{-M}]^+$, 121.1 (48%) $[D_7\text{-M}]^+$, 122.1 (17%) $[D_8\text{-M}]^+$, 123.2 (11%) $[D_9\text{-M}]^+$ and 124.2 (7%) $[D_{10}\text{-M}]^+$. These data are consistent with the literature values of the unlabelled compound.[8]

S.2.2 Synthesis of D_{14}-2-(diphenylphosphino)benzoic acid, [D_{14}]-10

Chlorodiphenylphosphine-D_{10}, [D_{10}]-8

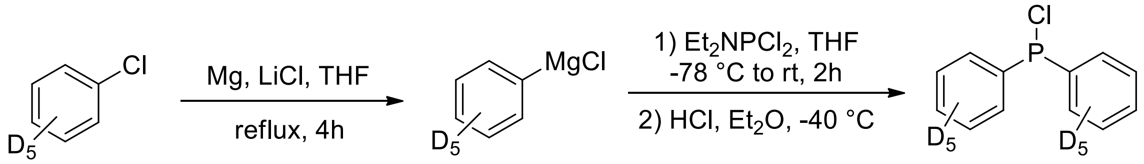

[D_5]-**4** [D_{10}]-**8**

Magnesium (0.975 g, 40.1 mmol) and lithium chloride (1.05 g, 24.8 mmol) were transferred to a dry 3-neck flask fitted with a reflux condenser under and then heated with a hot-air gun for 5 min *in vacuo*. The flask was allowed to cool and refilled with nitrogen. Chlorobenzene-D_5 [D_5]-**4** (1.81 g, 15.4 mmol, 2.13 equiv.) and deoxygenated anhydrous THF (2 mL) were added *via* syringe. The reaction was heated to 110 ˚C; after 1 hr further THF (4 mL) was added and heating was continued for another 1 hr. The reaction mixture was cooled in ice bath and diluted with THF (4 mL). The Grignard was transferred *via* cannula to a filtered solution of diethylphosphoramidous dichloride (1.05 mL, 1.26 g, 7.22 mmol, 1.00 equiv.) in THF (3 mL) at -78 ˚C under nitrogen atmosphere whilst stirring. The Grignard reagent was washed with THF (2 × 2 mL). The reaction was allowed to warm up to room temperature overnight and analysed by NMR (^{31}P NMR (122 MHz, unlocked) δ 62.24 (s, 1P, *N*,*N*-diethyl-1,1-diphenylphosphinamine-D_{10})). The reaction was cooled to -78 ˚C and HCl in diethyl ether (2.0 M, 11 mL, 21 mmol, 2.9 equiv.) was added drop-wise *via* syringe, during which heavy precipitation was observed. After 5 min the reaction was allowed to warm up to around -40 °C, and filtered while cold *via* cannula fitted with a filter paper into a dry Schlenk tube under nitrogen atmosphere. The orange-coloured filtrate was concentrated *in vacuo* at +25 °C to crude orange-brown oil [D_{10}]-**8**. The oily residue [D_{10}]-**8** was purified by distillation at 160-180°C, 1 torr to give colourless viscous oil with pungent odour [D_{10}]-**8** (0.483 g, 2.09 mmol, 29%). **^{31}P NMR** (122 MHz, unlocked) δ 80.38 (s, 1P). These data are consistent with the literature.[9]

***Tert*-butyl benzoate-D_5, [D_5]-6**

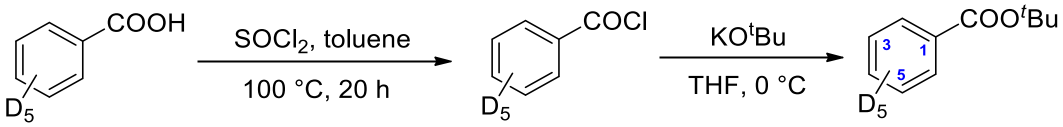

[D_5]-**3** [D_5]-**6**

Benzoic acid-D_5 [D_5]-**3** (2.507 g, 19.67 mmol, 1.0 equiv.) was suspended in anhydrous toluene (5 mL) in a dry 2-neck flask fitted with a reflux condenser. Thionyl chloride (5.5 mL, 9.01 g, 75.8 mmol, 3.85 equiv.) was added *via* syringe and the reaction mixture was heated at 100 ˚C for 20 h. The reaction was cooled down and concentrated *in vacuo*, re-dissolved in toluene (5 mL) and the volatile organic compounds were removed *in vacuo*. Acyl chloride solution was diluted with anhydrous THF (10 mL) and cooled in an ice bath under the atmosphere of nitrogen. A solution of potassium *tert*-butoxide (4.00 g, 35.6 mmol, 2.10 equiv.) in anhydrous THF (10 mL) was added *via* cannula whilst stirring; a precipitation of potassium chloride was observed. After 4 h the reaction was quenched with aqueous saturated $NaHCO_3$, and diluted with ethyl acetate. The layers were separated and the aqueous fraction was extracted with ethyl acetate. The organic fractions were combined and washed with brine (10 mL), dried over $MgSO_4$ and concentrated *in vacuo* at 10 ºC to crude yellow oil [D_5]-**6,** that was purified by Kugelrhohr distillation (148 ˚C at 3.5 torr) to give a colourless oil [D_5]-**6** (2.997 g, 16.5 mmol, 84%). **^{1}H NMR** (400 MHz, $CDCl_3$): δ 1.61 (s, 9H, 3 × C***H$_3$***); **^{2}H NMR** (77 MHz, unlocked) δ 8.00 (s, 2D, C(2,6)***D***), 7.53 (s, 1D, C(4)***D***) and 7.42 (s, 2D, C(3,5)***D***); **^{13}C NMR** (100 MHz, $CDCl_3$): δ 165.8 (***C***=O), 131.9 (1:1:1 t, $^1J_{C\text{-}D}$ = 24.9 Hz, ***C***(4)D), 131.9 (s, ***C***(1)), 129.0 (1:1:1 t, $^1J_{C\text{-}D}$ = 24.5 Hz, ***C***(2,6)D), 127.6 (1:1:1 t, $^1J_{C\text{-}D}$ = 24.9 Hz, ***C***(3,5)D), 80.9 (s, -***C***(CH_3)$_3$) and 28.2 (s, 3 × ***C***H_3); **MS (ESI$^+$)** *m/z* 206.12 [M+Na]$^+$. These data are consistent with that expected on the basis of data reported for the unlabelled compound.[10]

(TMP)$_2$MgCl · 2LiCl

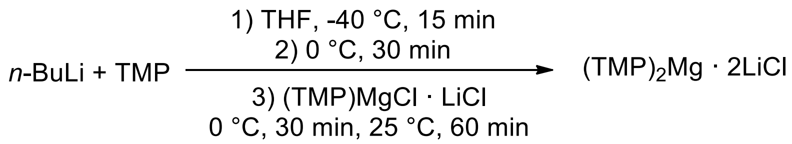

Anhydrous deoxygenated THF (12 mL) was added to a dry Schlenk tube fitted with a magnetic stirrer under the atmosphere of nitrogen, and then cooled to -40 ˚C. *n*-BuLi (2.34 M, 5.1 mL, 12.0 mmol, 1.0 equiv.) was added immediately *via* gas-tight syringe. The mixture was stirred for 30 min at this temperature before freshly distilled 2,2,6,6-tetramethylpiperidine (1.70 g, 12.0 mmol, 1.0 equiv.) was added *via* syringe. After 10 min the reaction was transferred to an ice bath, and stirred for another 50 min. TMPMgCl · LiCl in THF (0.85 M, 14.1 mL, 12.0 mmol) was added *via* syringe. The resulting clear green-brown reaction mixture was stirred for 30 min, and then another 60 min at 25 ˚C. The cloudy pink-brown suspension was concentrated *in vacuo* to light brown solid that was re-dissolved in anhydrous deoxygenated THF (16 mL) and stirred for 10 min at 25 ˚C. The clear, red-brown (TMP)$_2$MgCl · 2LiCl solution was titrated with benzoic acid and (4-phenylazo)diphenylamine indicator in THF. (1.14 N concentration reading was obtained.)

***Tert*-butyl 2-iodobenzoate-D_4, [D_4]-7**

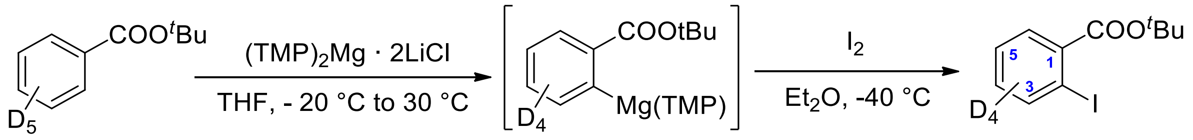

[D_5]-**6** [D_4]-**7**

Tert-butyl benzoate-D_5 [D_5]-**6** (1.47 g, 8.01 mmol, 1.0 equiv.) was dried over $MgSO_4$, dissolved in THF (10 mL) under atmosphere of nitrogen and cooled in ice bath. $(TMP)_2MgCl \cdot 2LiCl$ (0.57 N, 31 mL) was added *via* syringe over 6 min. The reaction was slowly heated up to +26 °C over 2 days (80% conv. by NMR). The mixture was cooled to -30 ˚C and a solution of iodine (4.52 g, 17.8 mmol) in diethyl ether was carefully added *via* cannula while maintaining stirring. After 2 h the reaction was then allowed to warm up to room temperature and 50 min later the excess iodine was quenched with saturated aqueous $Na_2S_2O_3$. The organic fraction was separated and washed twice with dilute aqueous $Na_2S_2O_3$, dilute aqueous HCl (2 ×), aqueous $NaHCO_3$ (2 ×), distilled water and brine. The orange coloured solution was dried over $MgSO_4$ and concentrated *in vacuo* to crude brown oil [D_4]-**7**. The material [D_4]-**53** was purified on silica-gel eluting with *n*-pentane – diethyl ether 25:1 to give [D_4]-**7** (1.22 g, 3.96 mmol, 49%) as clear light yellow oil. **^{1}H NMR** (400 MHz, $CDCl_3$) δ 1.63 (s, 9H, 3 × C***H$_3$***); **^{2}H NMR** (46 MHz, unlocked) *δ* 7.92 (s, 1D, ***C***(6)D), 7.67 (s, 1D, ***C***(3)D), 7.36 (s, 1D, ***C***(4)D) and 7.09 (s, 1D, ***C***(5)D); **^{13}C NMR** (101 MHz, $CDCl_3$) *δ* 166.15 (s, 1C, ***C***=O), 82.62 (s, 1C, -***C***$(CH_3)_3$), 28.13 (s, 3C, 3 × ***C***H_3); **IR** 2278 (w), 2273 (w, C-D), 1720 (s, C=O), 1556 (w), 1531 (w), 1477 (w), 1456 (w), 1393 (w), 1367 (s), 1323 (m), 1475 (m), 1244 (s), 1156 (s), 1078 (s), 1035 (w), 990 (m), 847 (s) and 746 (w) cm^{-1}; **MS (ESI$^+$)** *m/z* 311.12 (100%) and 331.01 (5%) $[M+Na]^+$. These data are consistent with that expected on the basis of data reported for the unlabelled compound.[11]

Tert-butyl D_{14}-2-(diphenylphosphino)benzoate, [D_{14}]-9

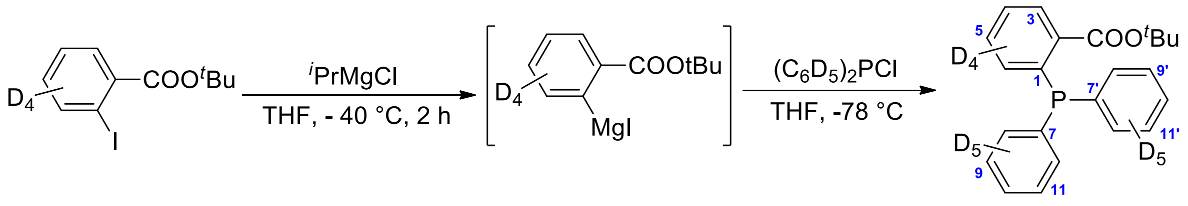

[D_4]-**7** [D_{14}]-**9**

Tert-butyl 2-iodobenzoate-D_4 [D_4]-**7** (1.22 g, 3.96 mmol, 1.00 equiv.) was dissolved in deoxygenated THF (10 mL) in a dry Schlenk tube fitted with magnetic stirrer under an atmosphere of nitrogen. The mixture was cooled to -50 ˚C and *iso*-propylmganessium chloride in THF (1.44 M, 2.9 mL, 4.2 mmol, 1.05 equiv.) was added drop-wise over 5 min. The reaction was stirred at that temperature for 100 min, and then cooled to -78 ˚C. (C_6D_5)PCl [D_{10}]-**8** (1.05 g, 4.57 mmol, 1.15 equiv.) solution in deoxygenated THF (5 mL) was added drop-wise *via* syringe over 10 min. The reaction was left to slowly warm up to room temperature. The reaction was confirmed to be complete by ^{31}P NMR. The reaction was quenched with deoxygenated aqueous saturated ammonium chloride (7 mL). The mixture was diluted with deoxygenated distilled water (5 mL) and extracted with deoxygenated diethyl ether (3 × 10-15 mL) and transferred to a dry Schlenk *via* cannula. The organic extracts were combined and dried over $MgSO_4$, then concentrated *in vacuo*. Crude [D_{14}]-**9** was triturated with deoxygenated hexane (2 × 15mL), and the white solid [D_{14}]-**9** was crystallized from a minimum amount of hot anhydrous deoxygenated ethanol to yield [D_{14}]-**9** (1.013 mg, 2.69 mmol, 68%) as white crystals. **^{1}H NMR** (500 MHz,) δ 1.37 (s, 9H, (C***H***$_3$)$_3$); **^{31}P NMR** (121 MHz, unlocked) δ -5.05 (s, 1P); **^{13}C NMR** (126 MHz, $CDCl_3$) δ 166.60 (s, 1C, ***C***=O), 139.27 (d, $^1J_{CP}$ = 25.9 Hz, 1C, P-***C***(1)), 138.05 (d, $^1J_{CP}$ = 12.2 Hz, 2C, P-***C***(7,7')), 136.46 (d, $^2J_{CP}$ = 18.3 Hz, 1C, ***C***(1)-C(=O)), 134.05-133.06 (m, Ar-***C***D), 128.33-127.39 (m, Ar-***C***D), 82.03 (s, 1C, -***C***(CH_3)$_3$), 27.86 (s, 3C, 3 × ***C***H_3); **^{2}H NMR** (46 MHz, unlocked) δ 7.85 (br. s, Ar-D), 7.23 (br. s, Ar-D) and 6.79 (br. s, Ar-D); ; **IR** 2984 (w), 2273 (w, C-D), 1694 (s, C=O), 1552 (w), 1474 (w), 1454 (w), 1364 (m), 1333 (m), 1307 (w), 1280 (m), 1253 (s), 1154 (s), 1076 (s), 1033 (w), 956 (w), 832 (s), 743 (m) and 719 (m) cm^{-1}; **MS (ESI$^+$)** *m/z* 321.18 [M-^{t}Bu+H_2]$^+$ and 377.24 [M+H]$^+$. These data are consistent with that expected on the basis of data reported for the unlabelled compound.[12]

D_{14}-2-(diphenylphosphino)benzoic acid, [D_{14}]-10

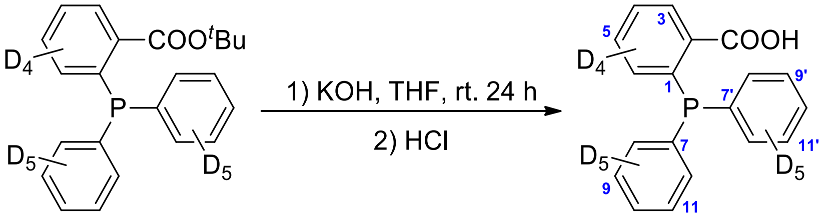

[D_{14}]-**9** [D_{14}]-**10**

Tert-butyl D_{14}-2-(diphenylphosphino)benzoate, [D_{14}]-**9** (1.010 g, 2.685 mmol) and large excess of freshly ground potassium hydroxide (1.10 g, 19.6 mmol) were mixed with laboratory grade deoxygenated THF (20 mL) under an atmosphere of nitrogen. The reaction was stirred at room temperature overnight, when thick precipitate formed. The volatile organic compounds were removed *in vacuo*, and the solid residue was re-suspended in deoxygenated distilled water (40 mL) and deoxygenated Et_2O (30 mL) and then acidified to pH = 1 with HCl in Et_2O, before the organic layer was removed *via* cannula. The aqueous layer was extracted twice more with deoxygenated Et_2O (3 × 10 mL). The organic layers were combined and dried over $MgSO_4$, then concentrated *in vacuo* to a yellowish powder [D_{14}]-**10**. The material [D_{14}]-**10** was crystallised from a minimal amount of deoxygenated boiling methanol (5.5 mL). The crystals were washed with cool methanol (1.0 mL), crushed and dried *in vacuo* at 87 ˚C for 24 h to remove traces of solvent. [D_{14}]-**10** (431 mg, 1.34 mmol, 50%). **Mp** 160-164 ºC; **^{31}P NMR** (202 MHz, $CDCl_3$) δ -4.78 (s, 1P); **^{2}H NMR** (46 MHz, unlocked) δ 7.92 (br. s, 1D), 7.09 (br. s, 12D) and 6.75 (br. s, 1D); **^{13}C NMR** (126 MHz, $CDCl_3$) δ 170.6 (s, 1C, -***C***OOH), 142.1 (d, $^{1}J_{CP}$ = 27.8 Hz, 1C, P-***C***(1)), 137.6 (d, J_{CP} = 10.3 Hz, 2C, P-***C***(7,7')), 134.2-133.0 (m, Ar-***C***D), 133.0-132.0 (m, Ar-***C***D), 128.7-127.8 (m, Ar-***C***D); **IR** 2848 (m), 2586 (m) 2284 (m), 2272 (m), 2258 (m, C-D), 1684 (s, C=O), 1551 (w), 1528 (m), 1419 (s), 1359 (m), 1290 (s), 1254 (s), 1081 (m), 1006 (w), 927 (s), 832 (s), 823 (s) and 786 (w) cm^{-1}; **MS (ESI^{+})** *m/z* 303.16 $[M-OH]^{+}$, 321.17 $[MH]^{+}$, 343.16 $[M+Na]^{+}$ and 359.13 $[M+K]^{+}$; **CHN** found: C, 71.604; H, 5.017. These data are consistent with that expected on the basis of data reported for the unlabelled compound.[13,14]

S.2.3 Amide bond coupling

D_{38}-*S,S*-DACH-phenyl Trost Ligand, [D_{38}]-(*S,S*)-2

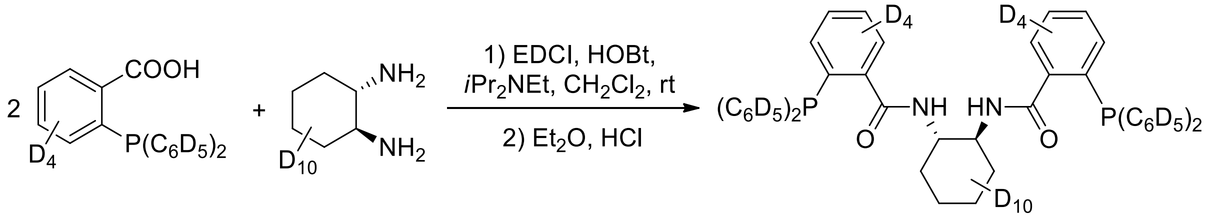

[D_{14}]-**10** [D_{10}]-(*S*,*S*)-**15** [D_{38}]-(*S*,*S*)-**2**

D_{14}-2-(diphenylphosphino)benzoic acid [D_{14}]-**10** (161 mg, 0.504 mmol, 2.0 equiv.), EDCI · HCl (145 mg, 0.756 mmol, 3.0 equiv.), HOBt · H_2O (125 mg, 0.817 mmol, 3.2 equiv.) and Hunig's base (0.20 mL, 1.2 mmol, 4.8 equiv.) were dissolved in anhydrous deoxygenated dichloromethane (5 mL) under an atmosphere of nitrogen at room temperature. After 45 min a solution of D_{10}-(*S,S*)-1,2-diaminocyclohexane [D_{10}]-(*S,S*)-**15** (31.2 mg, 0.252 mmol, 1.0 equiv, as determined by ^{2}H NMR assay) in anhydrous deoxygenated dichloromethane (2.4 mL) was added. The reaction was stirred for 23 h, before the reaction was diluted with deoxygenated diethyl ether (15 mL) and washed with HCl aq. (2 × 3 mL, 1.2 M), distilled water (3 × 3 mL), $NaHCO_3$ aq. (3 × 3 mL), water (3 mL) and brine (3 mL). The organic phase was then dried over $MgSO_4$ and concentrated *in vacuo.* The off-white solid was crystallised from boiling acetonitrile. Colourless cubic crystals were crushed and dried *in vacuo* at 50 °C for 3 h. White powder [D_{38}]-(*S,S*)-**2** (55 mg, 0.076 mmol, 30%). **^{31}P NMR** (121 MHz, unlocked) δ -10.17 (s, 2P); **^{2}H NMR** (46 MHz, unlocked) δ 7.23 (br. s, 28D, Ar-D), 3.64 (br. s, 2D, 2 × C***D***-NH-), 1.63 (br. s, 4D, 2 × -C***D₂***-) and 1.06 (br. s, 4D, 2 × -C***D₂***-); **^{13}C NMR** (126 MHz, CD_2Cl_2) δ 169.54 (s, 2C, 2 × ***C***=O), 141.32 (d, *J* = 24.4 Hz, Ar-***C***), 138.52 (d, *J* = 11.8 Hz, Ar-***C***), 137.38 (d, *J* = 22.1 Hz, Ar-***C***), 135.10–133.06 (m, Ar-***C***), 130.51–129.57 (m, Ar-***C***), 129.17–127.88 (m, Ar-***C***), 127.99–127.10 (m, Ar-***C***), 32.11–30.80 (m, Alk-***C***), 30.26 (s, Alk-***C***) and 24.78–23.47 (m, Alk-***C***); **MS (ESI$^+$)** *m/z* 727.49 (5%) [D_{36}-M+H]$^+$, 728.49 (30%) [D_{37}-M+H]$^+$, 729.50 (100%) [D_{38}-M+H]$^+$, 730.50 (44%) and 731.51 (10%). These data are consistent with that expected on the basis of data reported for the unlabelled compound.[15]

S.2.4 Synthesis of palladium complexes, 1

Bis(η^3-cyclohexenylpalladium(II)) dichloride, 16

Method A:

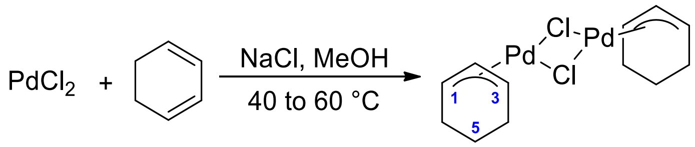

16

$PdCl_2$ (0.500 g, 2.82 mmol, 1.0 equiv.) and NaCl (0.330 g, 5.64 mmol, 2.0 equiv.) were dried under vacuum in a Schlenk tube. The vessel was refilled with nitrogen and then anhydrous, deoxygenated methanol (20 mL) was added. The Schlenk tube was fitted with a cold finger and the reaction was heated to 40 ˚C. 1,3-Cyclohexadiene (1.74 g, 2.07 mL, 21.7 mmol, 7.7 equiv.) was added and the reaction heated to 60 ˚C for 30 min. The reaction mixture became yellow-green and precipitation of greenish crystals was observed. The reaction was allowed to cool to room temperature and then poured into water (50 mL) and toluene (50 mL). The layers were separated and the aqueous phase was extracted with toluene (2 × 50 mL). The organic fractions were combined and washed with water (50 mL), saturated $NaHCO_3$ aq. (50 mL), brine (50 mL) and dried over $MgSO_4$. The mixture was concentrated *in vacuo* to yield crude yellow-green amorphous solid **16**. The crude product **16** was dissolved in CH_2Cl_2 (5 mL) and purified on a plug of silica-gel (70 mm × 70 mm, pre-mixed with *n*-hexane), washing with *n*-hexane (200 mL) followed by elution with CH_2Cl_2. The yellow fractions were combined and the solvent removed *in vacuo* to give a yellow glass-like solid (0.524 g, 83%), that was recrystallized by layering a solution of **16** in CH_2Cl_2 (7 mL) with *n*-pentane (55 mL) to yield the desired product **16** (0.431 g, 9.67 mmol, 69%) as yellow needle-like crystals. The product **16** was stored at -4 ºC. **^{1}H NMR** (400 MHz, $CDCl_3$): δ 5.49 (t, 1H, J = 6.4 Hz, C(2)***H***), 5.21 (ddd, 2H, J = 1.7, 6.3, 6.4 Hz, C(1,3)***H***), 1.91-1.70 (m, 5H, 5 × C***H***), 1.12-0.97 (m, 1H, C***H***); **^{1}H NMR** (400 MHz, CD_3CN) δ 5.52 (t, J = 6.4 Hz, 1H, C(2)***H***), 5.15 (dd, J = 6.1, 6.1 Hz, 2H, C(1,3)***H***), 1.90-1.78 (m, 2H, 2 × C***H***), 1.75-1.63 (m, 3H, 3 × C***H***), 1.09-0.94 (m, 1H, C***H***); **^{13}C NMR** (100 MHz, $CDCl_3$): δ 100.8 (s, ***C***(2)H), 77.9 (s, ***C***(1,3)H), 27.8 (s, ***C***(4,6)H_2) and 18.4 (s, ***C***(5)H_2). These data are consistent with the literature.[16, 17]

Method B:

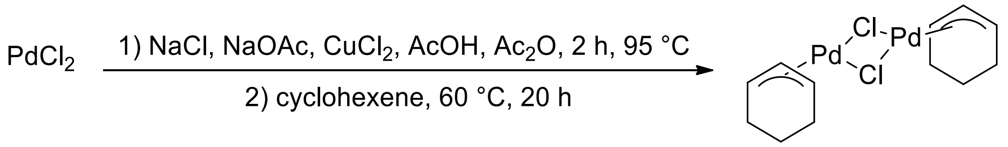

16

Glacial acetic acid (50 mL) was mixed with acetic anhydride (1 mL) and nitrogen was bubbled through the solution. Copper(II) chloride (1.12 g, 8.33 mmol, 7.31 equiv.) was dried under vacuum at 125 ˚C for 2 hours prior to use.

Anhydrous acetic acid (50 mL) was added to a mixture of sodium chloride (0.880 g, 15.1 mmol, 13.2 equiv.), palladium(II) chloride (203 mg, 1.14 mmol, 1.00 equiv.) and sodium acetate (1.20 g, 14.6 mmol, 12.8 equiv.) under an atmosphere of nitrogen. The mixture was heated to 95 ˚C overnight, before copper(II) chloride (1.12 g, 8.33 mmol, 7.31 equiv.) and heating was maintained for further 2 hours and the resulting deep green coloured reaction mixture was cooled to 60 ˚C. Cyclohexene **5** (0.97 g, 1.2 mL, 12 mmol, 10 equiv.) was added *via* syringe immediately, and the reaction was stirred overnight at 60 ˚C, then allowed to cool down to room temperature. The reaction mixture was exposed to air, diluted with distilled oxygenated water (100 mL) and extracted with dichloromethane (2 × 20 mL). Bright yellow clear organic fraction was filtered under air and washed with saturated $NaHCO_3$ aq. (70 mL) and brine (70 mL). The organic fraction was then dried over $MgSO_4$, filtered through a silica-gel plug pre-mixed with dichloromethane and concentrated *in vacuo.* The crude solid residue **16** was washed with *n*-hexane to yield the desired product **16** (163 mg, 0.351 mmol, 61%) as bright yellow solid.

Bis(η^3-D_9-cyclohexenylpalladium(II)) dichloride, [D_{18}]-16

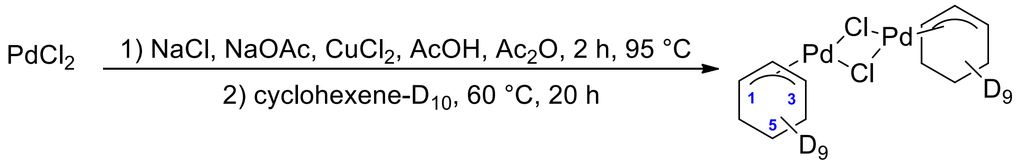

[D_{18}]-**16**

Glacial acetic acid (110 mL) was mixed with acetic anhydride (30 mL) and nitrogen was bubbled through the solution. Copper(II) chloride (1.502 g, 7.44 mmol) was dried under vacuum at 125 ˚C for 2 hours prior to use. Sodium acetate (1.560 g, 19.0 mmol) was dried under vacuum at room temperature for 2 hours. Cyclohexene-D_{10} [D_{10}]-**5** (estimated 0.88 g in *n*-pentane, 11 mmol) was pre-mixed with acetic acid (5 mL).

Dry sodium chloride (1.10 g, 18.8 mmol) and sodium acetate (1.56 g, 19.0 mmol) were added to a dry 2-neck round bottomed flask fitted with a reflux condenser and magnetic follower. The flask was evacuated under vacuum and refilled with nitrogen. Anhydrous acetic acid (40 mL) was added and the mixture heated to 90 ˚C. Copper(II) chloride (1.50 g, 7.44 mmol) in acetic acid (10 mL) was added. The resulting deep green solution was heated for 10 min and then palladium(II) chloride (260 mg, 1.47 mmol) was added and washed with acetic acid (20 mL). The heating was maintained for another 155 min, and then reduced to 60 ˚C. Excess of cyclohexene-D_{10} [D_{10}]-**5** in acetic acid was added *via* syringe, and the reaction was stirred for 11 hr 40 min, then allowed to cool down to room temperature. The reaction mixture was filtered under air. The yellow-green filtrate was poured into cool distilled water (100 mL) and extracted with toluene (4 × 15 mL). The bright yellow clear organic fraction was washed with distilled water (50 mL), followed by sat. $NaHCO_3$ aq. (2 × 50 mL) and brine (2 × 50 mL), dried over $MgSO_4$ and concentrated *in vacuo.* The residue [D_{18}]-**16** was purified on silica-gel with $CHCl_3$. Yellow fractions were combined and concentrated *in vacuo* to give the desired product as yellow solid [D_{18}]-**16** (44.4 mg, 0.098 mmol, 13%). **^{2}H NMR** (77 MHz, unlocked, CH_2Cl_2) δ 5.49 (br. s, 1D, C(2)***D***), 5.12 (br. s, 2D, C(1,3)***D***), 1.69 (br. s, 5D, 5 × C***D***) and 0.95 (br. s, 1D, C***D***); **MS (ESI$^+$)** *m/z* 422.01 (15%), 423.01 (24%), 424.02 (33%), 425.01 (53%), 426.02 (81%), 427.02 (69%), 428.02 (100%), 429.02 (73%) [D_{18}-M-Cl]$^+$, 430.02 (80%), 431.03 (46%), 432.02 (41%), 433.03 (26%) and 435.03 (13%). These data are consistent with the literature. [18]

[Bis-acetonitrile(η^3-cyclohexenyl)palladium(II)] tetrakis(pentafluorophenyl)borate, 17-BAr$_F$

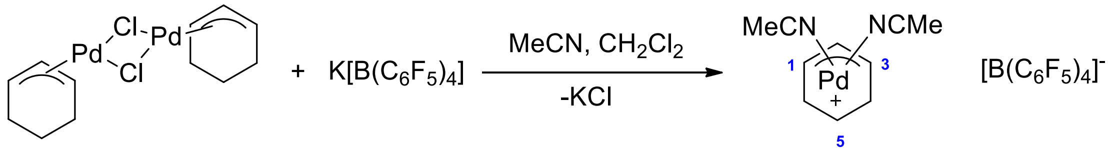

16 KBAr$_F$ [**17**][BAr$_F$]

Potassium tetrakis(pentafluorophenyl)borate (748 mg, 83% strength, 0.86 mmol, 2.0 equiv.) was dissolved in anhydrous deoxygenated CH_2Cl_2 (2.5 mL) and anhydrous deoxygenated acetonitrile (1.5 mL) under the atmosphere of nitrogen. The solution was transferred *via* cannula to a bright yellow solution of bis(η^3-cyclohexenylpalladium(II)) dichloride **16** (193 mg, 0.43 mmol, 1.0 equiv.) in anhydrous deoxygenated CH_2Cl_2 (2.5 mL) under N_2. After 5 min the resulting cloudy mixture was filtered through a cannula with a filter paper plug, and the volatile organic compounds were removed *in vacuo*. The yellowish foam was triturated with *n*-hexane (2 x 5 mL) to give the desired product [**17**][BAr$_F$] (735 mg, 0.774 mmol, 90%) as an off-white powder. The compound [**17**][BAr$_F$] was stored at -4 ºC under atmosphere of nitrogen to prevent decomposition. **^{1}H NMR** (301 MHz, CD_3CN) δ 5.63 (t, J = 6.5 Hz, 1H, C(2)***H***), 5.43 (dd, J = 6.2, 6.2 Hz, 2H, C(1,3)***H***), 2.16 (s, 6H, 2 × CH_3CN), 2.02-1.86 (m, 2H), 1.49-1.67 (m, 3H), 1.02-0.82 (m, 1H); **^{13}C NMR** (101 MHz, CD_3CN) δ 108.2 (s, 1C, ***C***(2)H), 82.3 (s, 2C, ***C***(1,3)H), 29.8 (s, 2C, ***C***(4,6)H_2), 19.0 (s, 1C, ***C***(5)H_2); **^{19}F NMR** (376 MHz, CD_3CN) δ -133.70 (br. s, 8F, 4 × BAr$_F$-C(2,6)***F***), -163.94 (t, $^3J_{FF}$ = 19.7 Hz, 4F, BAr$_F$-C(4)***F***), -168.30 (br. dd, $^3J_{FF}$ = 17.6, 17.6 Hz, 8F, 4 × BAr$_F$-C(3,5)***F***); **MS (ESI$^+$)** *m/z* 226.00 (32%), 227.00 (71%), 228.00 (91%), 229.00 (7%) $[C_6H_9CH_3CNPd]^+$, 230.00 (100%), 231.00 (8%), 232.00 (51%) and 233.00 (4%). These data are consistent with that expected on the basis of data reported for the [**17**][B(3,5-$(CF_3)_2$-C_6H_3))$_4$] compound.[18]

[Bis-acetonitrile(η^3-D_9-cyclohexenyl)palladium(II)] tetrakis(pentafluorophenyl)borate, [D_9]-17-BAr$_F$

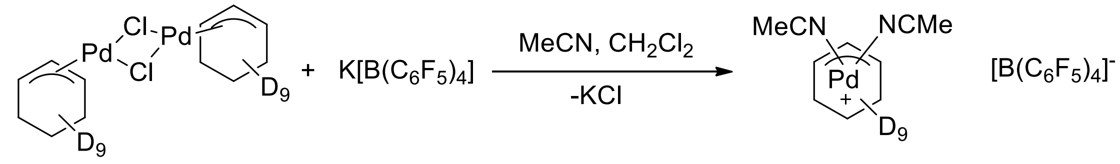

[D_{18}]-**16** KBAr$_F$ [[D_9]-**17**][BAr$_F$]

Potassium tetrakis(pentafluorophenyl)borate (88.2 mg, 0.123 mmol, 2.0 equiv.) was dissolved in anhydrous deoxygenated CH_2Cl_2 (0.7 mL) and anhydrous deoxygenated acetonitrile (0.5 mL) under the atmosphere of nitrogen. The solution was transferred *via* cannula to a bright yellow solution of bis(η^3-D_9-cyclohexenylpalladium(II)) dichloride [D_{18}]-**16** (38.5 mg, 0.0614 mmol, 1.0 equiv.) in anhydrous deoxygenated CH_2Cl_2 (0.7 mL) under N_2 and rinsed with acetonitrile (0.5 mL). After 55 min the resulting solution was filtered through a cannula with a filter paper plug, and the volatile organic compounds were removed *in vacuo.* The yellowish foam was triturated with *n*-hexane (4 × 3 mL) to give the desired product [[D_9]-**17**][BAr$_F$] (93.8 mg, 0.097 mmol, 79%) as slightly yellow powder and was used immediately in the following step.

[Bis-acetonitrile(η^3-cyclohexenyl)palladium(II)] trifluoromethanesulfonate, 17-OTf

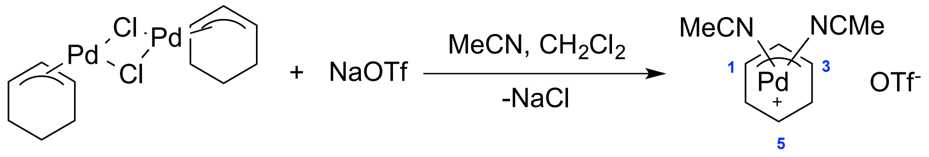

16 [**17**][OTf]

Sodium triflate (100 mg, 0.581 mmol, 2.0 equiv.) was dissolved in anhydrous deoxygenated acetonitrile (2.0 mL) under the atmosphere of nitrogen. The solution was transferred *via* gas tight syringe to a bright yellow solution of bis(η^3-cyclohexenylpalladium(II)) dichloride **16** (130 mg, 0.291 mmol, 1.0 equiv.) in anhydrous deoxygenated CH_2Cl_2 (2.0 mL) and acetonitrile (0.7 mL) under N_2. After 3 hr the resulting cloudy mixture was filtered through a cannula with a filter paper plug, and the volatile organic compounds were removed *in vacuo.* The yellowish foam was triturated with *n*-hexane (2 x 10 mL) to give the desired product [**17**][OTf] (220 mg, 0.526 mmol, 91%) as an air-sensitive off-white powder. The compound [**17**][OTf] was used immediately in the next step to avoid decomposition. **^{1}H NMR** (500 MHz, CD_3CN) δ 5.68 (t, J = 6.5 Hz, 1H, C(2)***H***), 5.44 (dd, J = 6.2, 6.2 Hz, 2H, C(1,3)***H***), 2.21 (s, 6H, 2 × CH_3CN), 2.02-1.93 (m, 2H), 1.74-1.60 (m, 3H), 1.08-0.95 (m, 1H); **^{13}C NMR** (126 MHz, CD_3CN) δ 101.0 (s, 1C, ***C***(2)H), 81.7 (s, 2C, ***C***(1,3)H), 29.7 (s, 2C, ***C***(4,6)H_2), 19.1 (s, 1C, ***C***(5)H_2); **^{19}F NMR** (470 MHz, CD_2Cl_2) δ -79.33 (s, 3F, OTf^-). These data are consistent with that expected on the basis of data reported for the [**17**][B(3,5-$(CF_3)_2$-C_6H_3)$_4$] compound.[18]

[(η^3-Cyclohexenyl)palladium(II){(1*R*,2*R*)-1,2-*N*-*N'*-bis[2'-(diphenylphosphino)benzoyl] diaminocyclohexane}] tetrakis(pentafluorophenyl)borate, (*R*,*R*)-1-BAr$_F$

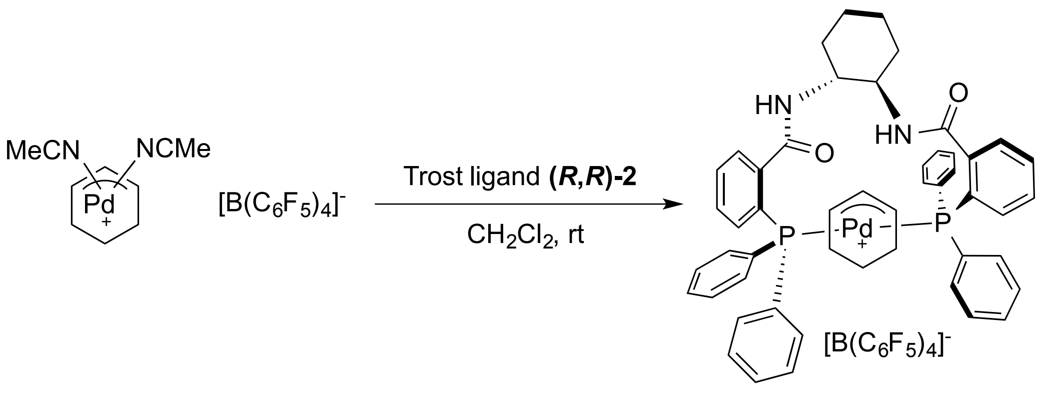

[**17**][BAr$_F$]   [(*R*,*R*)-**1**][BAr$_F$]

A solution of [bis-acetonitrile(η^3-cyclohexenyl)palladium(II)] tetrakis(pentafluorophenyl)borate [**17**][BAr$_F$] (0.630 g, 0.664 mmol, 1.02 equiv.) in anhydrous, deoxygenated CH_2Cl_2 (7.0 cm^3) was slowly transferred *via* cannula to a solution of (1*R*,2*R*)-DACH-phenyl Trost ligand (*R*,*R*)-**2** (0.448 g, 0.649 mmol, 1.00 equiv.) in anhydrous deoxygenated CH_2Cl_2 (7.0 cm^3) under an atmosphere of nitrogen. The golden yellow solution was stirred for 2 h, and then concentrated *in vacuo* to yield the desired product [(*R*,*R*)-**1**][BAr$_F$] (0.810 g, 0.520 mmol, 80%) as a bright yellow solid. Mp (decomp.): 174-180 °C; **^{1}H NMR** (500 MHz, CD_2Cl_2) δ 7.68-7.05 (m, 28H, Ar-H), 7.02-6.66 (m, 8H, Ar-H), 5.78 (br. d, J = 30.3 Hz, 2H), 5.24 (br. s, 1H), 4.44 (br. s, 1H), 3.76-3.57 (m, 2H), 2.19-0.97 (m, 14H); **^{31}P NMR** (202 MHz, CD_2Cl_2, 4.05 mM) δ 22.48 (d, $^2J_{PP}$ = 33.4 Hz, 1P) and 19.76 (d, $^2J_{PP}$ = 34.6 Hz, 1P); **^{19}F NMR** (376 MHz, CD_2Cl_2) δ -133.2 (br. s, 8F, 4 × BAr$_F$-C(2,6)***F***), -163.8 (t, $^3J_{FF}$ = 20.4 Hz, 4F, BAr$_F$-C(4)***F***) and -167.6 (br. dd, $^3J_{FF}$ = 18.0, 18.0 Hz, 8F, 4 × BAr$_F$-C(3,5)***F***); ^{13}C NMR (126 MHz, CD_2Cl_2) δ 147.6, 145.7, 137.7, 135.8, 133.8, 132.5, 131.7, 131.1, 129.8, 129.4, 128.8, 127.7, 126.6, 125.5, 122.1, 115.4, 111.4, 29.6, 26.4, 24.5, 22.8, 18.1; **IR** 3429 (w), 3058 (w), 2936 (w), 1643 (m), 1511 (s), 1459 (s), 1436 (s), 1374 (w), 1324 (w), 1274 (m, C-F), 1084 (s), 976 (s), 746 (s), 683 (s) and 660 (s) cm^{-1}; **MS (ESI$^+$)** *m/z* 771.33 [M-BAr$_F$-Pd]$^+$, 797.17 [M-BAr$_F$-C_6H_9]$^+$ and 877.24 [M-BAr$_F$]$^+$; **HRMS (ESI$^+$)** found 877.2300 [M-BAr$_F$]$^+$. These data are consistent with that expected on the basis of data reported for the [(*R*,*R*)-**1**][B(3,5-$(CF_3)_2$-C_6H_3))$_4$] compound.[18]

[(η^3-Cyclohexenyl)palladium(II){(1*S*,2S*)*-1,2-*N*-*N'*-bis[2'-(diphenylphosphino)benzoyl] diaminocyclohexane}] tetrakis(pentafluorophenyl)borate, (*S*,*S*)-1-BAr$_F$

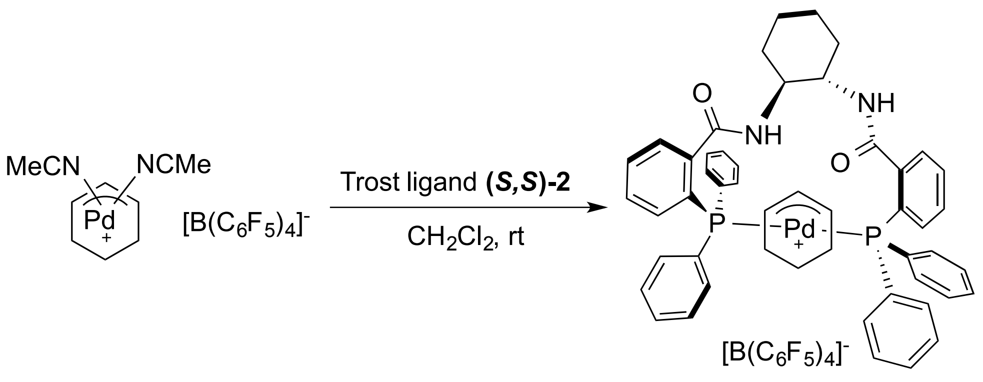

[**17**] [BAr$_F$]   [(*S*,*S*)-**1**] [BAr$_F$]

The compound [(*S*,*S*)-**1**][BAr$_F$] was synthesised following the same procedure as [(*R*,*R*)-**1**][BAr$_F$]. Yellow solid [(*S*,*S*)-**1**][BAr$_F$] (90 mg, 0.058 mmol, 47 %). **NMR** data identical to [(*R*,*R*)-**1**][BAr$_F$]. **MS (ESI$^+$)** *m/z* 771.33 [M-BAr$_F$-Pd]$^+$, 797.17 [M-BAr$_F$-C_6H_9]$^+$ and 877.23 [M-BAr$_F$]$^+$.

[(η^3-D_9-Cyclohexenyl)palladium(II){(1*S*,2*S*)-1,2-*N*-*N*'-bis[D_{14}-2'-(diphenylphosphino) benzoyl]diaminocyclohexane-D_{10}}] tetrakis(pentafluorophenyl)borate, [D_{47}]-(*S*,S)-1-BAr_F

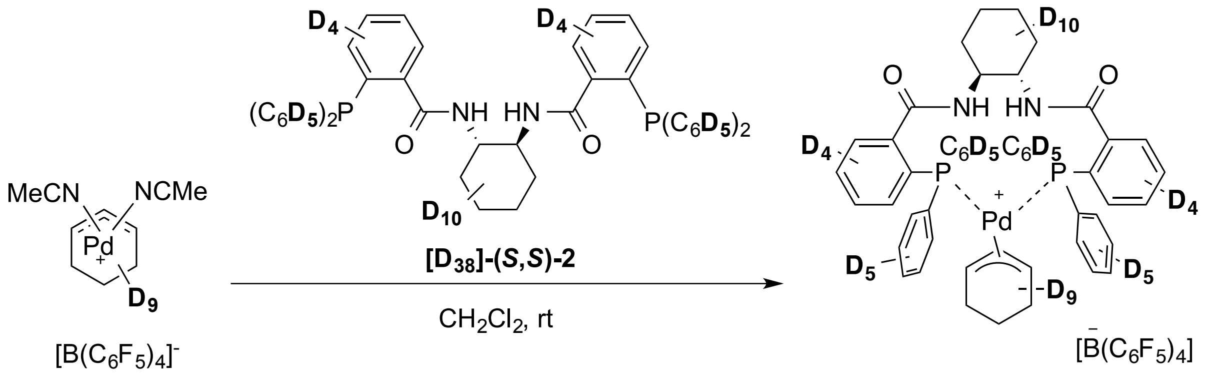

[[D_9]-**17**] [BAr_F] [[D_{47}]-(*S*,*S*)-1][BAr_F]

A solution of [[D_9]-**17**][BAr_F] (94 mg, 0.099 mmol, 1.0 equiv.) in anhydrous, deoxygenated CH_2Cl_2 (1.0 cm^3) was transferred *via* cannula to a solution of [D_{38}]-(*S*,*S*)-**2** (73 mg, 0.10 mmol, 1.0 equiv.) in anhydrous deoxygenated CH_2Cl_2 (7.0 cm^3) and rinsed with an additional (1.0 cm^3) under an atmosphere of nitrogen. The golden yellow solution was stirred for 2 h, and then slowly concentrated *in vacuo* to yield the desired product [[D_{47}]-(*S*,*S*)-**1**][BAr_F] (160 mg, 0.100 mmol, 99%) as light yellow-brown solid. Mp (decomp.): 174-180 ºC; **^{31}P NMR** (202 MHz, unlocked) δ 28.85–26.32 (m, 1P, oligomer), 26.32–23.50 (m, 1P, oligomer), 22.42 (d, $^2J_{PP}$ = 33.0 Hz, 1P, monomer), 19.67 (d, $^2J_{PP}$ = 33.0 Hz, 1P, monomer); **^{19}F NMR** (376 MHz, CD_2Cl_2) δ -140.3 (br. s, 8F, 4 × BAr_F-C(2,6)***F***), -170.9 (br. s, 4F, 4 × BAr_F-C(4)***F***) and -174.7 (br. s, 8F, 4 × BAr_F-C(3,5)***F***); **^{11}B NMR** (160 MHz, unlocked) δ -23.94 (s, 1B); **IR** 3428 (w), 2924 (w), 2279 (w), 2211 (w), 2112 (w), 1643 (m), 1510 (s), 1460 (s), 1374 (w), 1310 (w), 1274 (m, C-F), 1084 (s), 976 (s), 834 (m), 774 (m), 755 (m), 682 (m) and 660 (s) cm^{-1}; **MS (ESI$^+$)** *m/z* 920.49 (16%) [D_{42}-M-BAr_F]$^+$, 921.49 (40%) [D_{43}-M-BAr_F]$^+$, 922.49 (76%) [D_{44}-M-BAr_F]$^+$, 923.49 (100%) [D_{45}-M-BAr_F]$^+$, 924.49 (88%) [D_{46}-M-BAr_F]$^+$, 925.49 (68%) [D_{47}-M-BAr_F]$^+$, 926.49 (60%), 927.49 (36%), and 928.49 (23%). These data are consistent with that expected on the basis of data reported for the unlabelled [**1**][B(3,5-$(CF_3)_2$-C_6H_3))$_4$] compound.[18]

[(η^3-Cyclohexenyl)palladium(II){(1*S*,2*S*)-1,2-*N*-*N'*-bis[2'-(diphenylphosphino)benzoyl] diaminocyclohexane}] trifluoromethylsulfonate, (*S*,*S*)-1-OTf

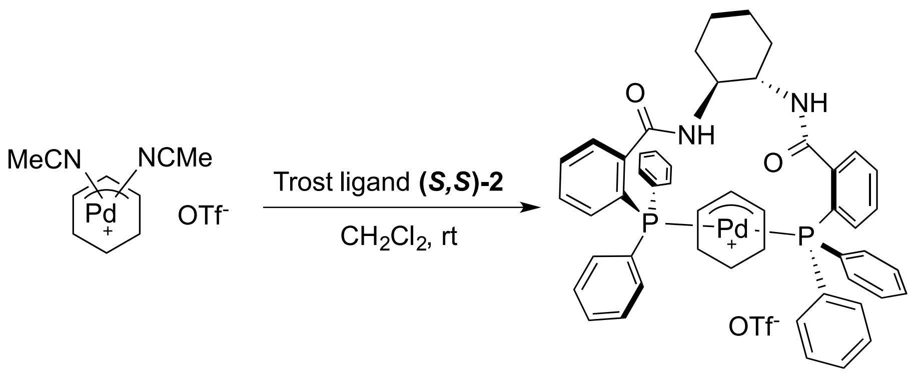

[**17**][OTf] [(*S*,*S*)-**1**][OTf]

A solution of (*S*,*S*)-**2** (0.361 g, 0.522 mmol, 1.00 equiv.) in anhydrous, deoxygenated CH_2Cl_2 (3.0 cm^3) was slowly transferred *via* syringe to a solution of [**17**][OTf] (0.224 g, 0.526 mmol, 1.02 equiv.) in anhydrous deoxygenated CH_2Cl_2 (3.0 cm^3) under an atmosphere of nitrogen. The golden yellow solution was stirred for 20 min, and then concentrated *in vacuo* and triturated with hexane to yield the desired product [(*S*,*S*)-**1**][OTf] (0.252 g, 0.246 mmol, 47%) as a bright yellow solid. **$^{31}P\{^1H\}$ NMR** (122 MHz, unlocked, CH_2Cl_2, 23 mM) δ 29.20-27.30 (m, 1P, oligomer), 26.00-24.50 (m, 1P, oligomer), 22.96 (d, J = 32.6 Hz, 1P, monomer) and 19.69 (d, J = 32.5 Hz, 1P, monomer); **^{19}F NMR** (283 MHz, CD_2Cl_2) δ -78.83 (s, 3F, OTf^-). These data are consistent with that expected on the basis of data reported for the [**1**][B(3,5-$(CF_3)_2$-C_6H_3)$)_4$] compound.[18]

S.2.5 Synthesis of other compounds

[(η^3-Cyclohexenyl)palladium(II){1,3-(diphenylphosphino)propane}] tetrakis(pentafluorophenyl)borate, 18-BAr$_F$

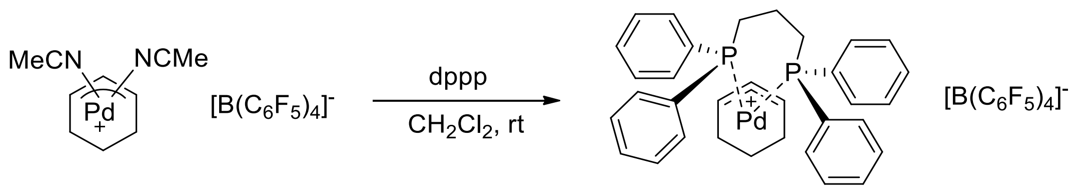

[17] [BAr$_F$] [18] [BAr$_F$]

[Bis-acetonitrile(η^3-cyclohexenyl)palladium(II)] tetrakis(pentafluorophenyl)borate [**17**][BAr$_F$] (261 mg, 0.275 mmol, 1.01 equiv.) and dppp (112 mg, 0.271 mmol, 1.00 equiv.) were dissolved in anhydrous deoxygenated dichloromethane (5 mL) under the atmosphere of nitrogen and stirred for 1 hour at room temperature. The volatile organic compounds were removed *in vacuo* to yield the desired complex [**18**][BAr$_F$] (294 mg, 0.230 mmol, 85%) as yellow-brown foam. **^{31}P NMR** (122 MHz, $CDCl_3$) δ 8.63 (s, 2P); **^{1}H NMR** (500 MHz, $CDCl_3$) δ 7.60-6.95 (m, 20H, Ar-H), 5.43 (dd, J = 7.0 Hz, 2H), 5.13 (br. s, 1H), 2.60 (ddd, J = 14.9, 7.5, 7.5 Hz, 2H), 2.48 (m, 2H), 2.38-0.73 (m, 8H); **^{13}C NMR** (126 MHz, $CDCl_3$) δ 133.4 (dd, J = 6.4 Hz), 132.3 (s), 131.0 (s), 130.7 (dd, J = 5.5 Hz), 129.5 (m), 112.8 (s), 89.8 (s), 27.4 (s), 27.0 (s), 19.7 (s), 18.35 (s); **IR** 2933 (w), 1644 (m), 1512 (s), 1459 (s), 1436 (s), 1374 (w), 1275 (m, C-F), 1083 (s), 975 (s), 774 (m), 756 (m), 742 (s), 692 (s) and 660 (s) cm^{-1}; **MS (ESI$^+$)** *m/z* 519.06 [M-BAr$_F$-C_6H_9]$^+$ and 599.12 [M-BAr$_F$]$^+$.

Cyclohex-2-en-1-yl acetate, 19

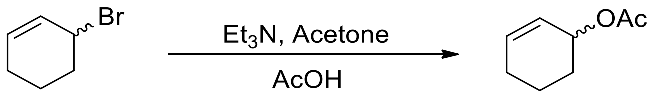

(±)-**19**

3-Bromocyclohex-1-ene (3.0 mL, 4.2 g, 26 mmol, 1.0 equiv.) was dissolved in acetone (50 mL, re-distilled over molecular sieves). The reaction was cooled to 0 °C and triethylamine (50 mL) was added drop-wise. After 50 min of stirring, glacial acetic acid (30 mL, with small aliquot of acetic anhydride) was added over 10 min at 0 °C. The reaction was left to warm up to room temperature overnight. The reaction mixture was cooled in ice bath and quenched with saturated aqueous $NaHCO_3$. The mixture was extracted with diethyl ether. The organic layer was washed with distilled water, brine, dried over magnesium sulphate and concentrated *in vacuo* at 0 °C to crude orange oil **19**. The material **19** was purified by column chromatography eluting with hexane : ethyl acetate 10 : 90 to colourless oil **19** (2.541 g, 18.15 mmol, 70%). **^{1}H NMR** (400 MHz, $CDCl_3$) δ 5.96 (dtd, J = 10.1, 3.8, 3.8, 1.2 Hz, 1H, -HC=C***H***-), 5.70 (ddt, J = 10.1, 4.0, 2.3, 2.3 Hz, 1H, -HC=C***H***-), 5.26 (dtd, J = 6.7, 3.7, 3.6, 1.6 Hz, 1H, -***H***C-O-), 2.05 (s, 3H, -C***H₃***), 2.14–1.93 (m, 2H, -C***H₂***-), 1.93–1.81 (m, 1H, -HC***H***-), 1.80–1.68 (m, 2H, -C***H₂***-) and 1.68–1.58 (m, 1H, -HC***H***-); **^{13}C NMR** (101 MHz, $CDCl_3$) δ 132.67 (=***C***H-CHO-), 125.67 (-***C***H=CHCHO-), 68.08 (-***C***H-O-), 28.28 (-***C***H_2-CHO-), 24.85 (-***C***H_2-), 21.41 (-C***H₃***) and 18.85 (-***C***H_2-). These data are consistent with the literature values.[19]

S.3 NMR

Oligomerisation of [(*R*,*R*)-1][BAr$_F$] in CD_2Cl_2

The solutions for ^{31}P NMR measurements were prepared by dissolving an accurate mass of [(*R*,*R*)-**1**][BAr$_F$] in a specific volume of dichloromethane-d$_2$, as listed in Table S1. The calculation of the monomer [**1**] and oligomer [(**1**)$_{oligomer}$] concentration was based on the integral values of the ^{31}P NMR spectra:

$$[\mathbf{1}]_{oligomer} = [\mathbf{1}]_{total} \times \frac{\sum oligomer\ integrals}{\sum all\ Pd - TSL\ integrals}$$

Table S1. **^{31}P NMR measurements of oligomerisation of [(*R*,*R*)-1][BAr$_F$] in CD_2Cl_2 at 25 °C**

$m_{1\text{-}BArF}$ / mg	$V_{solvent}$ / mL	$[1]_{total}$ / mM	$[1]_{oligomer}$ / mM	$[1]_{monomer}$ / mM
3.8	0.60	4.1	-	4.1
6.0	0.60	6.4	1.7	4.7
9.5	0.60	10.2	4.4	5.8
12.4	0.60	13.3	8.2	5.1
14.0	0.60	15.0	10.2	4.8
38.4	1.20	20.6	15.9	4.7
23.6	0.60	25.2	20.8	4.4
38.4	0.74	33.3	28.5	4.8
38.4	0.60	41.1	36.2	4.9
47.5	0.60	50.8	45.3	5.5

Oligomerisation of [(*R*,*R*)-1][BAr_F] in the presence of dppp-Pd complex [18][BAr_F] in CD_2Cl_2

Complex [(*R*,*R*)-**1**][BAr_F] (69.6 mg, 29.5 μmol) was dissolved in CD_2Cl_2 (3.0 mL) to prepare 14.9 mM stock solution. The ^{31}P NMR samples were prepared by dissolving the additive [**18**][BAr_F] in the stock solution of **1** (0.50 mL) as listed in Table S2.

Table S2. ^{31}P NMR sample preparation with additive 18 in CD_2Cl_2

Sample	m / mg	$[18]_{end}$ / mM	$(1)_n$ / %
1	-	-	67 ± 2
2	3.4	5.3	68 ± 3
3	6.3	9.9	72 ± 5
4	9.9	15.5	73 ± 7
5	12.8	20.0	72 ± 8

Oligomerisation of [(±)-1][BAr_F] in the presence of dppp-Pd complex [18][BAr_F] in CD_2Cl_2

A mixture of complex [(*R*,*R*)-**1**][BAr_F] (22.4 mg, 14.4 μmol) and [(*S*,*S*)-**1**][BAr_F] (22.7 mg, 14.6 μmol) was dissolved in CD_2Cl_2 (1.9 mL) to prepare 15.3 mM racemic stock solution. The ^{31}P NMR samples were prepared by dissolving the additive [**18**][BAr_F] in the stock solution of **1** (0.50 mL) as listed in Table S3.

Table S3. ^{31}P NMR sample preparation with additive 18 in CD_2Cl_2

Sample	m / mg	$[18]_{end}$ / mM	$(1)_n$ / %
1	-	-	51 ± 2
2	3.5	5.5	52 ± 3
3	Combined (2) + (4)	10.3	55 ± 5
4	9.7	15.2	53 ± 7

Oligomerisation of [(*R*,*R*)-1][BAr_F] in the presence of other palladium compounds in CD_2Cl_2

Complex [(*R*,*R*)-**1**][BAr_F] (46 mg, 29.5 µmol) was dissolved in CD_2Cl_2 (2.3 mL) to prepare 12.8 mM stock solution. The ^{31}P NMR samples were prepared by dissolving the additives (6.5 µmol) in the stock solution of **1** (0.50 mL) as listed in Table S4.

Table S4. ^{31}P NMR sample preparation with additives in CD_2Cl_2

Sample	Additive	m / mg	$(1)_n$ / %
1	-	-	61 ± 1
2	[dppe-Pd(C_6H_9)][BAr_F]	8.2	71 ± 1
3	[dppb-Pd(C_6H_9)][BAr_F]	8.4	73 ± 1
4	[dpppent-Pd(C_6H_9)][BAr_F]	8.7	70 ± 2
5	[dppf-Pd(C_6H_9)][BAr_F]	8.8	51 ± 1

Oligomerisation of [(*R*,*R*)-1][BAr_F] in the presence of additives in CD_2Cl_2

Complex [(*R*,*R*)-**1**][BAr_F] (77 mg, 49.5 µmol) was dissolved in CD_2Cl_2 (3.3 mL) to prepare 15.0 mM stock solution. The ^{31}P NMR samples were prepared by dissolving the molten salt additives in the stock solution of **1** (0.50 mL) as listed in Table S5.

Table S5. ^{31}P NMR sample preparation with molten salt additives in CD_2Cl_2.

Entry	Additive	m / mg	n / µmol	$[Additive]_{end}$ / mM	$(1)_n$ / %
1	-	-	-	-	68 ± 1
2	^{n}Bu$_4$N BF$_4$	3.4	10.3	20.6 ± 0.3	73 ± 1
3	^{n}Bu$_4$N PF$_6$	3.2	8.2	16.5 ± 0.3	78 ± 1
4	^{n}Bu$_4$N OTf	3.2	8.2	16.3 ± 0.3	72 ± 1
5	IMes BF$_4$	4.1	9.1	18.3 ± 0.3	73 ± 1
6	IMes PF$_6$	3.7	9.4	18.9 ± 0.3	76 ± 1

IMes = 1,3-dimesitylimidazolium

Oligomerisation of [(*R*,*R*)-1][X] in different solvents

The solutions for ^{31}P NMR measurements were prepared by dissolving an accurate mass of enantio-enriched salt [(*R*,*R*)-**1**][X] in specific volume of solvent to achieve the desired end-concentrations listed in Table S6. Samples in non-deuterated solvents were run unlocked.

Table S6. ^{31}P NMR measurements of oligomerisation of 1 in selected solvents at 25 °C

Solvent	Anion	[1] / mM #	% olig	$[1]_{oligomer}$ / mM	$[1]_{monomer}$ / mM
**Hexane**	BAr_F	Insoluble	-	-	-
**Et_2O**	BAr_F	Insoluble	-	-	-
**Dioxane**	BAr_F	Insoluble	-	-	-
**C_6F_6**	BAr_F	Insoluble	-	-	-
**$CHCl_3$**	BAr_F	Insoluble	-	-	-
**THF-d_8**	BAr_F	5.0	78	3.88	1.12
**THF-d_8**	BAr_F	14.9	92	13.7	1.18
**THF-d_8**	BAr_F	29.4	98	28.7	0.71
**THF-d_8**	BAr_F	62.8	99	62.2	0.56
**THF-d_8**	OTf	Insoluble	-	-	-
**CD_2Cl_2**	OTf	15	83	12.4	2.6
**CD_2Cl_2**	Krossing	20	70	14.0	6.0
**THF + *o*-$C_6F_2H_4$**	BAr_F	15	95	14.3	0.72
***o*-$C_6H_4F_2$**	BAr_F	15	79	11.8	3.16
***m*-$C_6H_4F_2$**	BAr_F	Insoluble	-	-	-
**MeCN + THF**	BAr_F	15	91	13.7	1.32
**DMF**	BAr_F	15	93	13.9	1.06
**MeCN-d_3**	BAr_F	15	92	13.8	1.17
**MeCN-d_3**	OTf	25	96	24.0	1.00
**$MeNO_2$**	BAr_F	15	81	12.1	2.86
**DMSO-d_6**	BAr_F	15	95	14.2	0.79
**MeCN + D_2O**	OTf	15	100	15.0	-
**MeCN + D_2O**	BAr_F	Insoluble	-	-	-
**D_2O**	OTf	Insoluble	-	-	-

# - ± 0.3 mM

S.4 SANS

Small-angle neutron scattering data were obtained on the D11, D16, D22 and D33 instruments at Institut Laue-Langevin (France), and on the SANS2D and LOQ instruments at ISIS at the Rutherford Appleton Laboratory (UK).

All samples in deuterated solvents were prepared in 2 mm path-length Hellma quartz cuvettes, and the rest were made up in 1 mm path-length Hellma cuvettes. The desired concentration of the samples was achieved by dissolving an accurate mass of compounds in an accurate volume of solvent. If necessary, the samples were filtered through a thin plug of cotton wool and were then transferred into the cells.

The samples were thermostated at 25 ˚C, unless specified otherwise. Appropriate detector masking was used to remove data affected by detector element imperfections. The scattering signals were corrected for transmission, empty cell and solvent background. The data were fitted with the least squares FISH[20] and SasView[21] software, using ROD and Cylinder models, respectively.

S.4.1 SANS Justification

Scattering length densities are specific for each chemically different material, and depend on the density, d, molecular weight, M_w, and the sum of coherent scattering lengths, $b_{i,coh}$, of the nuclei in the molecule **(1)**:

$$\rho_{coh} = \frac{1}{V_m}\sum_i b_{i,coh} = \frac{dN_A}{M_w}\sum_i b_{i,coh} \quad \textbf{(1)}$$

Table S7. Neutron scattering lengths, *b* of selected isotopes[22]

Isotope	Spin	Nat. Abundance, %	b_{coh}, fm	b_{incoh}, fm
**H**		---	-3.7390	---
**^{1}H**	½(+)	99.985	**-3.7406**	25.274
**^{2}H**	1(+)	0.015	**6.671**	4.04
**B**		---	5.30-0.213i	---
**C**		---	6.6460	---
**^{12}C**	0(+)	98.9	6.6511	0
**^{13}C**	½(-)	1.1	6.19	-0.52
**N**		---	9.36	---
**^{14}N**	1(+)	99.63	9.37	2.0
**O**		---	5.803	---
**^{16}O**	0(+)	99.762	5.803	0
**^{19}F**	½(+)	100	5.654	-0.082
**^{23}Na**	1½(+)	100	3.63	3.59
**^{31}P**	½(+)	100	5.13	0.2
**^{32}S**	0(+)	95.02	2.804	0
**Cl**		---	9.5770	---
**^{35}Cl**	1½(+)	75.77	11.65	6.1
**^{37}Cl**	1½(+)	24.23	3.08	0.1

**Pd**		---	5.91	---
**^{102}Pd**	0(+)	1.02	7.7(7)	0
**^{104}Pd**	0(+)	11.14	7.7(7)	0
**^{105}Pd**	2½(+)	22.33	5.5	-2.6(1.6)
**^{106}Pd**	0(+)	27.33	6.4	0
**^{108}Pd**	0(+)	26.46	4.1	0
**^{110}Pd**	0(+)	11.72	7.7(7)	0

The scattering lengths of proton and deuterium are very different, which makes the SANS technique suitable for "neutron contrast" experiments, where the sample particles are dispersed in deuterated media or vice versa.

The normalised SANS scattering intensity profile, $I(Q)$ of monodisperse homogeneous particles is defined by the equation **(2)**:[23]

$$I(Q) = \phi_p \Delta\rho^2 V_p P(Q,R) S(Q) \quad \textbf{(2)}$$

where Φ_p is the volume fraction of the scattering particles, and $\Delta\rho^2 = (\rho_p - \rho_{solvent})^2$ is the difference in the scattering length densities between the aggregates and the solvent. The ($\phi_p \Delta\rho^2 V_p$) term is also known as the scale factor, S_F, and may be used to validate data analysis. $P(Q,R)$ is a single particle form factor arising from the intra-particle scattering and encodes the information about the shape and size of aggregate. Inter-particle interactions give rise to the structure factor, $S(Q)$ but may be often negligible in dilute systems, where $S(Q) \approx 1$.

The Guinier approximation **(3)** holds true at low Q-range (**Guinier regime: $Q_{max}R_g \leq 1$**) of the scattering data for dilute, non-interacting systems and provides the radius of gyration, R_g of the particles:

$$P(Q,R) = 1 - \frac{Q^2 R_g^2}{3} \quad \textbf{(3)}$$

R_g is the root mean square of the radius of the particle, and relates to the dimensions of the cylinders, or rods **(5)**:

$$R_g = \frac{L}{\sqrt{12}} \quad \textbf{(4)}$$

Over the Guinier regime, when $S(Q) = 1$, the equations **(2)** and **(3)** can be combined:

$$I(Q) \approx \phi_p \Delta\rho^2 V_p \left(1 - \frac{Q^2 R_g^2}{3}\right) \approx \phi_p \Delta\rho^2 V_p e^{\left(-\frac{Q^2 R_g^2}{3}\right)} \quad \textbf{(5)}$$

For spherical particles a Guinier plot of ln[$I(Q)$] versus Q^2 should include a linear section, where $QR_g \leq 1$, with the slope = $-\frac{R_g^2}{3}$. A general expression of the Guinier approximation **(6)** is particle shape dependent:

$$I(Q) \propto Q^{-D} e^{\left(-\frac{Q^2 R^2}{K}\right)} \quad \textbf{(6)}$$

where D = 4 for cylinders, and the radius can be obtained by plotting **(7)**:

$$\ln[I(Q)Q] \text{ vs. } Q^2; \qquad R_{cylinder} = \sqrt{slope\ x\ 4} \quad \textbf{(7)}$$

Computer-based fitting software including FISH[24] and SASVIEW[25] uses the least-squares iterative method (**8**) of fitting the experimental data:

$$P(Q) = V_p^2 \Delta\rho^2 \int_0^{\frac{\pi}{2}} \frac{[\sin^2(\frac{1}{2}QL\cos\beta][4]J_1^2(QR\sin\beta)]}{[\frac{1}{4}(QL)^2\cos^2\beta][(QR)^2\sin^2\beta]} \sin\beta \, d\beta \quad \textbf{(8)}$$

where $J_1()$ is a Bessel function of the first kind and β is the angle between the scattering vector and the rod axis.

S.4.2 Kinetic experiments

The kinetic SANS experiments were planned to follow the changes in [(*R*,*R*)-**1**][BAr_F] structure during the allylic alkylation reaction. The model substrate cyclohexenyl acetate is known to participate in the oxidative addition step of the catalytic cycle regenerating cyclohexenylpalladium(II) Trost ligand intermediate $[(R,R)\text{-}\mathbf{1}]^+$. The majority of previous SANS studies were conducted in THF-D_8; also THF is usually a solvent of choice for the target reaction.

Initially, tetra-*n*-butylammonium malonate was selected as a candidate nucleophile in THF-D_8. However, the compound displayed a flat, strong scattering profile across a broad *Q* range, which would have obscured the Trost complex [(*R*,*R*)-**1**][BAr_F] signals.

Alternatively, phenol derivatives are also known to participate in the allylic alkylation cycle. Nucleophile 4-methoxyphenol makes only a minor contribution to background scattering levels.

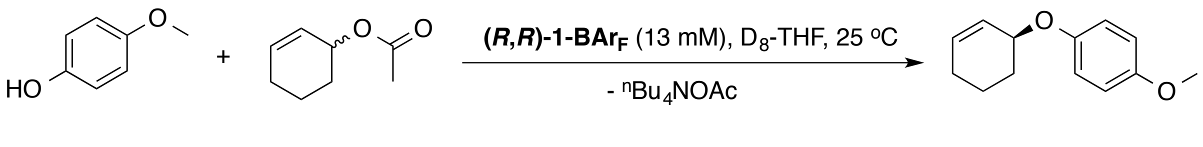

(±)-**19** **20**

EXP 1) A reaction mixture containing 4-methoxyphenol (14 ± 0.5 mg, 0.11 mmol, 161 mM), cyclohex-2-en-1-yl acetate **19** (14 ± 0.5 mg, 0.10 mmol, 143 mM) and complex [(*R*,*R*)-**1**][BAr_F] (14 ± 0.5 mg, 9.0 µmol, 13 mM) in D_8-THF (0.70 ± 0.01 mL) was prepared in 2 mm path-length cell. The reaction was followed by SANS.

The scattering profiles of the reaction mixture did not change over the course of the reaction as shown in Figure S1. The sample remained homogeneous. The results indicated that the overall oligomeric catalyst [(*R*,*R*)-**1**]+ architecture and aggregation remained unchanged.

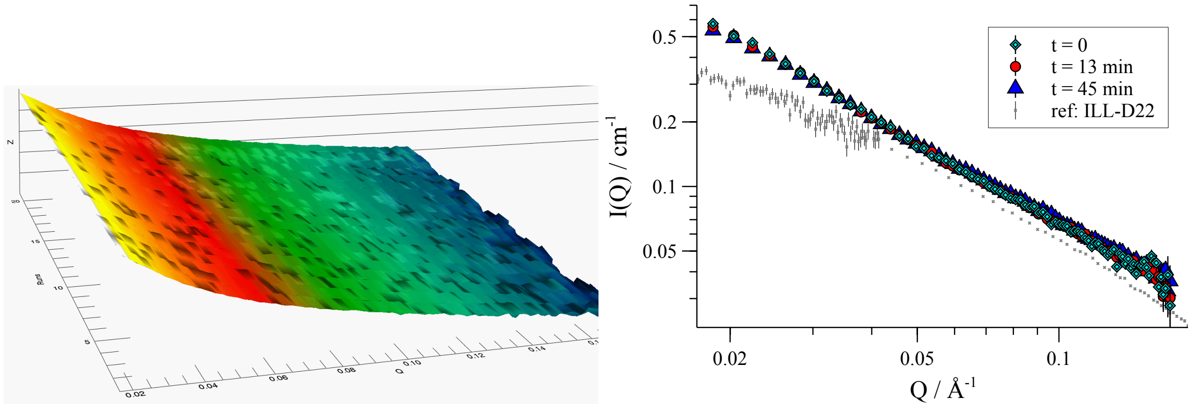

Figure S1. SANS kinetic experiment with complex [(*R*,*R*)-1][BAr_F] (13 mM) with 4-methoxyphenol (161 mM) and cyclohexenyl acetate 19 (143 mM) in THF-D_8 at 25 °C. D33, ILL

The reaction scattering profiles were consistent with infinitely long cylinders [(*R*,*R*)-**1**][BAr_F]. The Trost complex [(*R*,*R*)-**1**][BAr_F] structure is known to be very sensitive to solvent properties and quality as well as any additive. For example, moisture or polymerisation of THF-D_8 could have contributed to the shape of the scattering profiles. Additionally, the [(*R*,*R*)-**1**][BAr_F] batch-to-batch variability of the complex and slightly different experimental setup or sensitivity could have contributed to slightly different scattering.

EXP 2A) Tetra-*n*-butylammonium chloride (100 mg, 0.360 mmol) was mixed with sodium dimethyl malonate (56 mg, 0.364 mmol) in THF (2.8 mL). The mixture was filtered to give 130 mM solution of tetra-*n*-butylammonium malonate **21**. Cyclohex-2-en-1-yl acetate **19** (50 mg, 0.360 mmol) was then added.

Complex [(*R*,*R*)-**1**][BAr_F] (8 ± 0.5 mg, 5.1 µmol, 14 mM) was dissolved in the 130 mM stock solution of substrate **19** and malonate nucleophile **21** (0.350 ± 0.01 mL) in 1 mm path-length cell. The reaction was followed by SANS.

EXP 2B) Complex [(*R*,*R*)-**1**][BAr_F] (8 ± 0.5 mg, 5.1 µmol, 14 mM) was dissolved in 130 mM stock solution of substrate **19** and malonate nucleophile **21** (0.175 ± 0.01 mL) and THF (0.175 ± 0.01 mL) in 1 mm path-length cell. The reaction was followed by SANS.

Malonate as nucleophile was then tested in THF-H_8. While scattering was still significant in the high-*Q* region, and despite increased noise owing to the protonated solvent, it was still possible to resolve the Trost complex [(*R*,*R*)-**1**][BAr_F] at lower *Q* values. The experiment was setup with a 1:10 excess of both nucleophile **21** and substrate **(±)-19**.

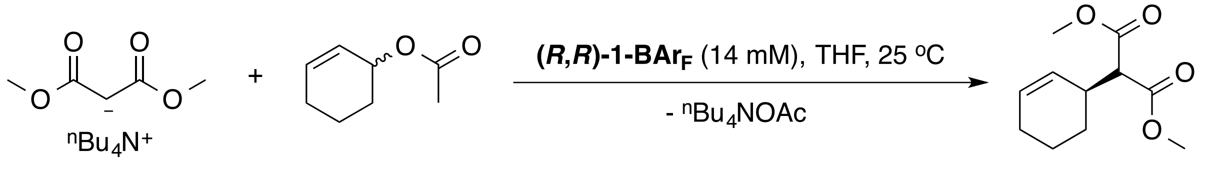

21 **(±)-19** **22**

Over time a slight increase in scattering was observed in the low-*Q* section, with a sharp transition after around 20 min (Figure S2; the data acquisition time was increased from 2 to 5 min – leading to increased *I*(*Q*) values). Also the reaction produced a small amount of tetra-*n*-butylammonium acetate precipitate. The observation of ^{31}P NMR data of Trost complex [(*R*,*R*)-**1**][BAr_F] mixtures with various additives such as salts and ionic liquids in all cases indicated an increase in the extent of aggregation. It is possible that a similar effect was also observed in this experiment. There was no major shift in the scattering curve shape recorded; therefore the cylindrical shape of aggregates [(*R*,*R*)-**1**][BAr_F] was retained.

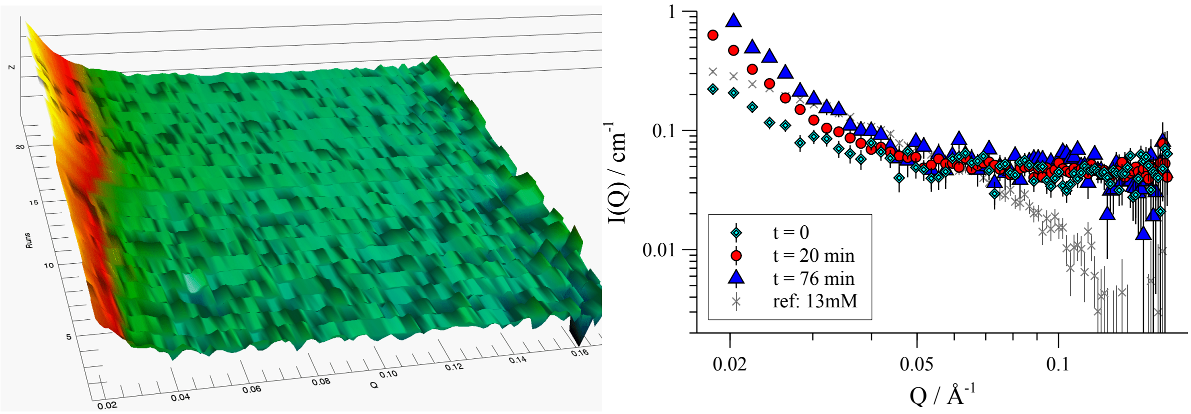

Figure S2. SANS kinetic experiment of [(*R*,*R*)-1][BAr$_F$] (14 mM) with tetra-*n*-butyl malonate 21 (130 mM) and cyclohexenyl acetate 19 (130 mM) in THF at 25 °C. D33, ILL

The experiments were also attempted in CD_2Cl_2. However due to excessive background noise and strong scattering by the nucleophile **21** the reactions could not be followed by SANS.

In conclusion, the SANS experiments revealed that no significant structural changes occurred to the Trost ligand palladium complex **1** during the catalytic cycle. While the effects of substrate background scattering, varying acquisition times and a shorter *Q*-range slightly affected the accuracy of data, it remains clear that the predominant structures in THF solution were long cylinders for the whole duration of these kinetic experiments.

S.5 MM3

Molecular modeling of **(1)$_n$** complexes was performed using a modified[18, 26,27] MM3* force field in Schrödinger MacroModel[28] software. A dielectric constant of $\varepsilon = 9$ and 35 was used as a very rough representation of dichloromethane and acetonitrile solvents, respectively. The cut-off distances were set to 99.0 Å for both Van der Waals and electrostatic interactions, and 4.0 Å – for H-bonds. The minimization was carried out using *truncated-Newton conjugate-gradient* (TNCG) method with convergence gradient threshold maximum value set to 0.05. Molecular Dynamics simulations were set up over a range of 100-300 ps time frame at the desired temperature using "SHAKE bonds to H constrained" parameter. The MD simulations were sampled at fixed-time intervals to generate a matrix of 25-100 structures.

Modifications to mm3.fld file[18]

The code to be added to the mm3.fld file[18] is listed in bold between the solid black lines (the position is indicated in *italic* in the following format: [*new line positions*]*d*[*old line position*]):

134d133 ("Stretching Interactions" section)

```
 1  SX - OM              1.4500    8.4120    2.9300  0202 0000           A 3          S(=O)-OM
```

316,317d314 ("Bending Interactions" section)

```
 2  C3 - SX - OM       107.7000    0.5970    0.2400  0000 0000 0000      A 3          C-S(=O)-OM
 2  O2 = SX - OM       116.6000    0.9000    0.2400  0000 0000 0000      A 3          O=S-OM
```

1074d1070 ("Van der Waals Interactions" section)

```
  Li     1.6000    0.0160    0.0000                              0000    A 3
```

2012,2025d2007 ("Special Structures" section)

```
 C  Barf
 9  C2-B3-C2*C2
-2
 1   1   2               1.4000    4.0000    0.0000
 2   1   2   3         110.0000    0.5000
 4   1   2   3   4       0.0000    0.0000    0.0000
-3
 C  BF4
 9  F0-B3-F0
-2
 1   1   2               1.4300    7.8000   -2.7000
 2   1   2   3         110.0000    0.5000
-3
```

Optimisation of Structures (1)$_n$

The initial [(±)-**1**][BAr_F] input model was based on the X-Ray crystal structure of the racemic allyl tetramer [(±)-**2**-Pd(C_3H_5)][OTf] in 1,1,2,2-tetrachloroethane. The input structures were also modified as required to represent enantio-enriched ([(*R*,*R*)-**1**][X])$_n$ and ([(*S*,*S*)-**1**][X])$_n$ oligomer cycles of various sizes, where anion X = BAr_F, OTf and Cl.

The models were first optimised in MacroModel using MM3 force field to local energy minima and were then shaken with Molecular Dynamics simulation. The resulting structures were re-optimised at MM3 theory level; the procedure was repeated if necessary.

Aggregate models $\{([\mathbf{1}][X])_4\}_m$ were generated by duplicating and stacking the MM3-optimised structures of oligomer rings $([\mathbf{1}][X])_4$ or smaller aggregates $\{([\mathbf{1}][X])_4\}_{m/2}$. The models were then re-optimised as described above.

S.6 Appendices

S.6.1 NMR spectra

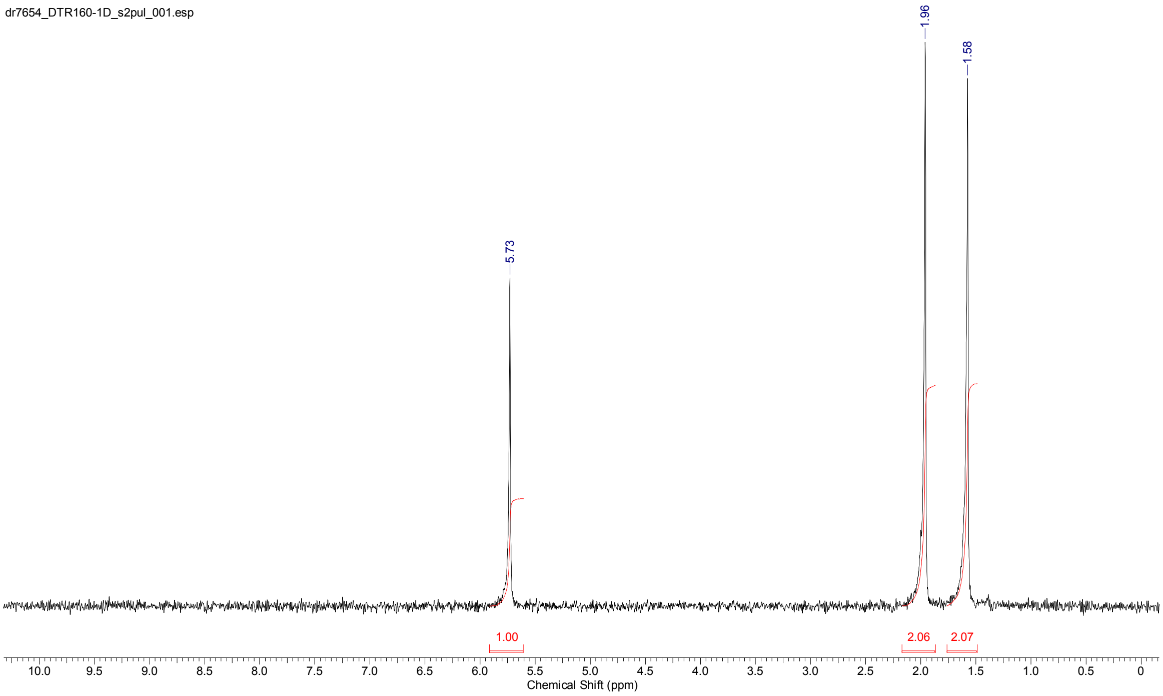

Figure S3. ^{2}**H NMR (77 MHz, unlocked) spectrum of cyclohexene-D$_{10}$, [D$_{10}$]-5**

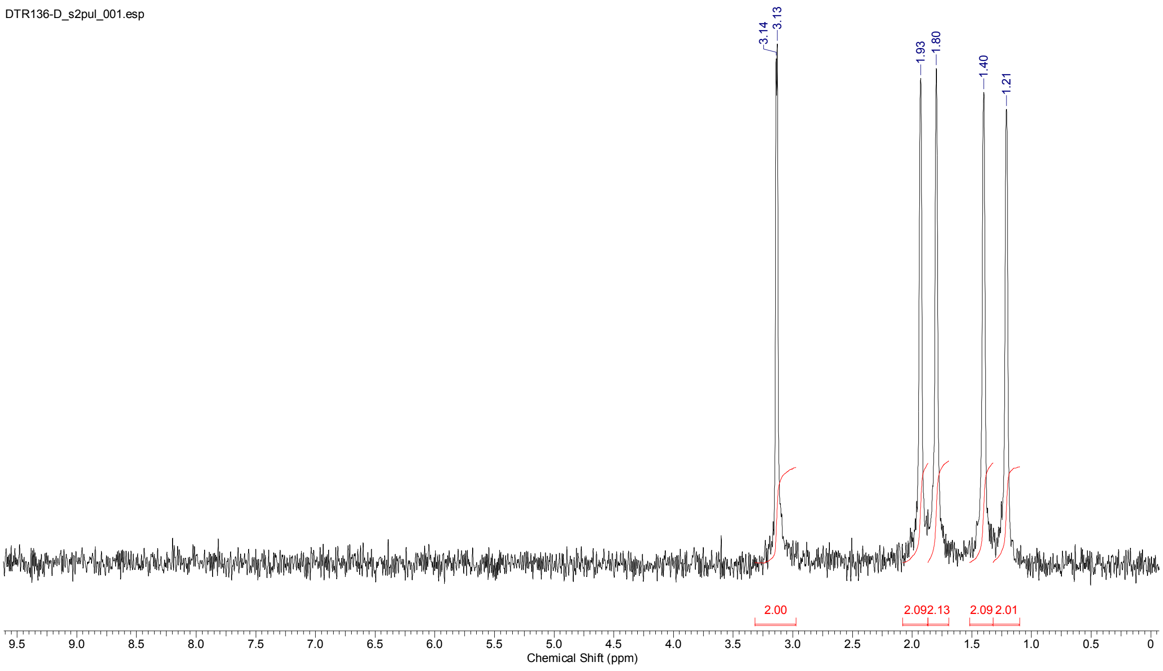

Figure S4. ^{2}**H NMR (77 MHz, unlocked) spectrum of cyclohexene oxide-D$_{10}$, [D$_{10}$]-11**

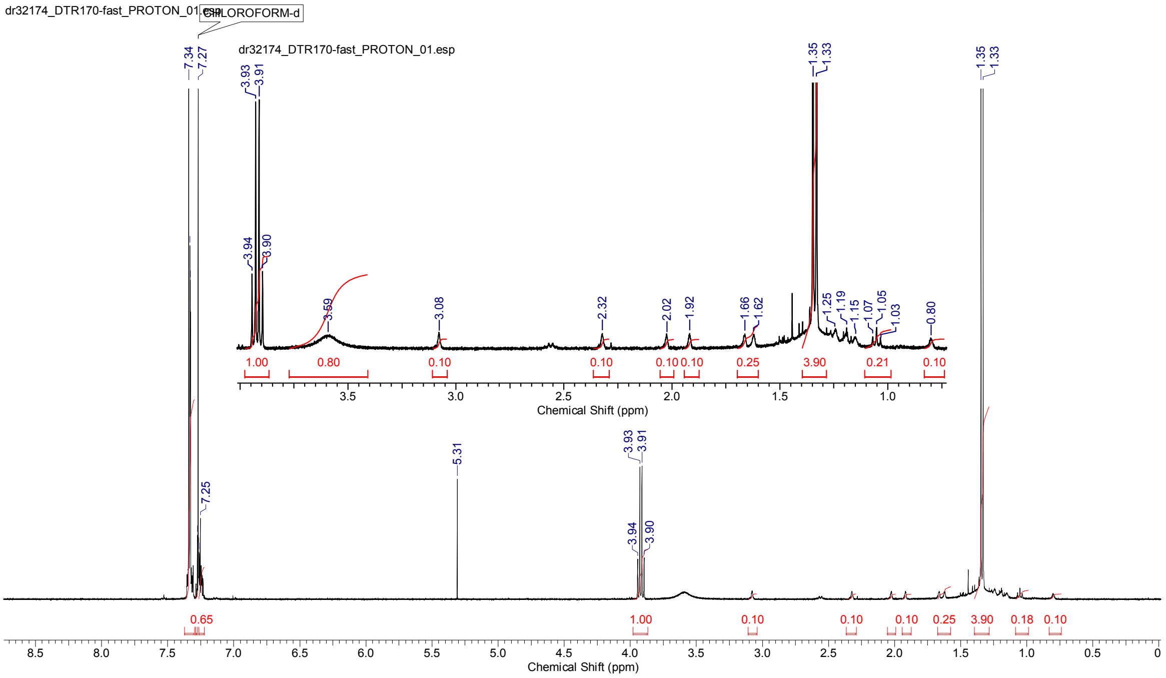

Figure S5. ^{1}H **NMR (400 MHz,** $CDCl_3$**) spectrum of *trans*-2-(((*R*)-1-phenylethyl)amino)cyclohexanol-**D_{10}**,** $[D_{10}]$**-[*R*]-12**

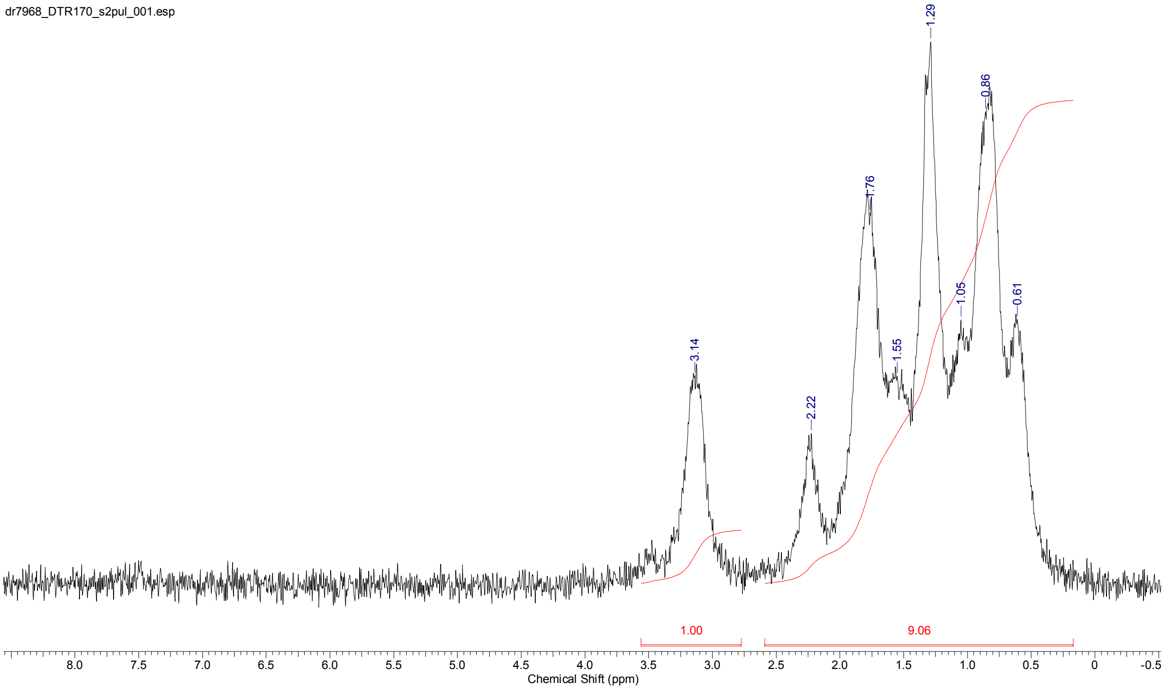

Figure S6. ^{2}H **NMR (77 MHz, unlocked) spectrum of *trans*-2-(((*R*)-1-phenylethyl)amino)cyclohexanol-**D_{10}**,** $[D_{10}]$**-[*R*]-12**

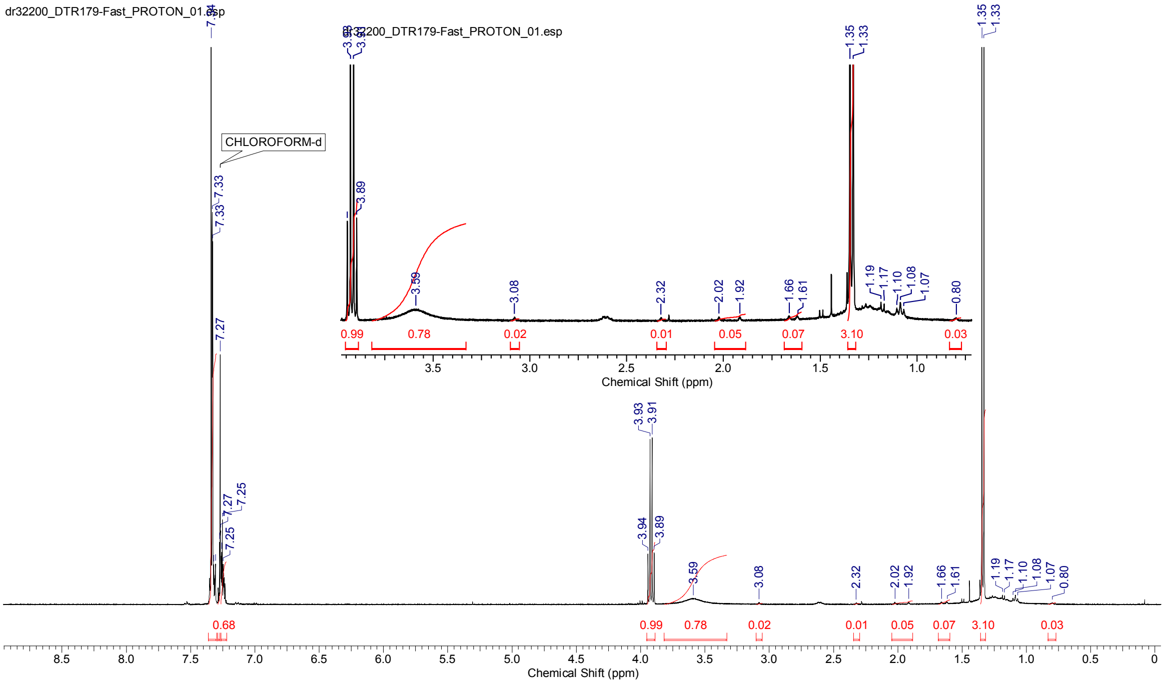

Figure S7. **^{1}H NMR (400 MHz, $CDCl_3$) spectrum of *trans*-2-(((*S*)-1-phenylethyl)amino)cyclohexanol-D_{10}, [D_{10}]-[*S*]-12**

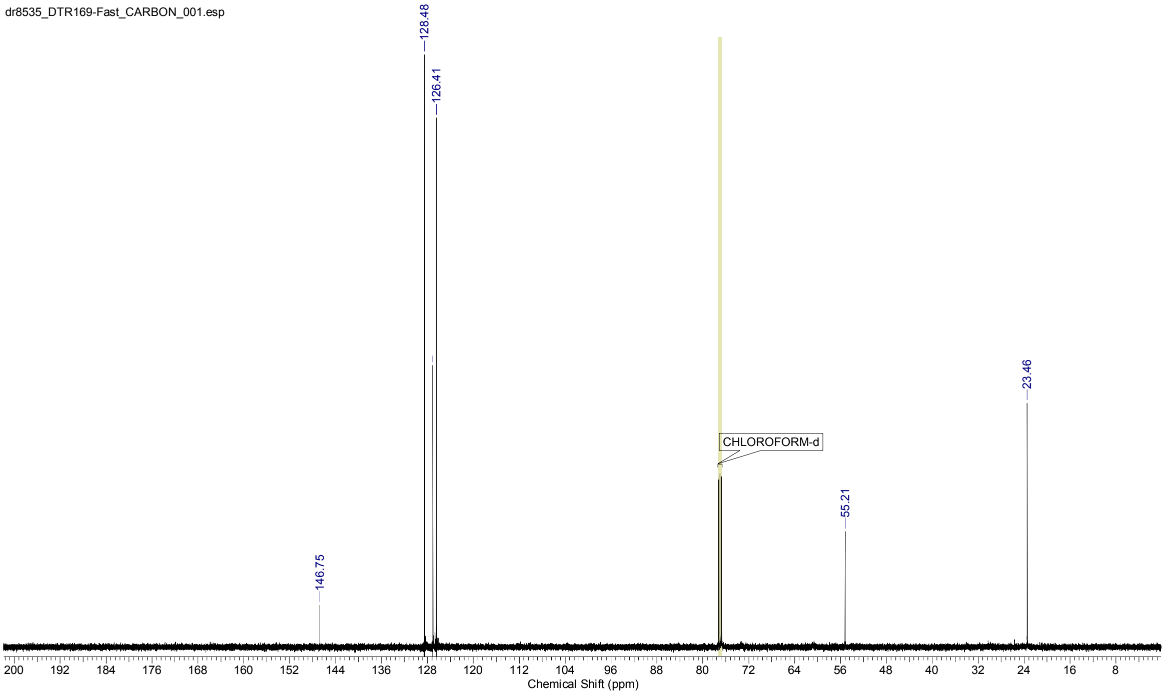

Figure S8. **^{13}C NMR (126 MHz, $CDCl_3$) spectrum of *trans*-2-(((*S*)-1-phenylethyl)amino)cyclohexanol-D_{10}, [D_{10}]-[*S*]-12**

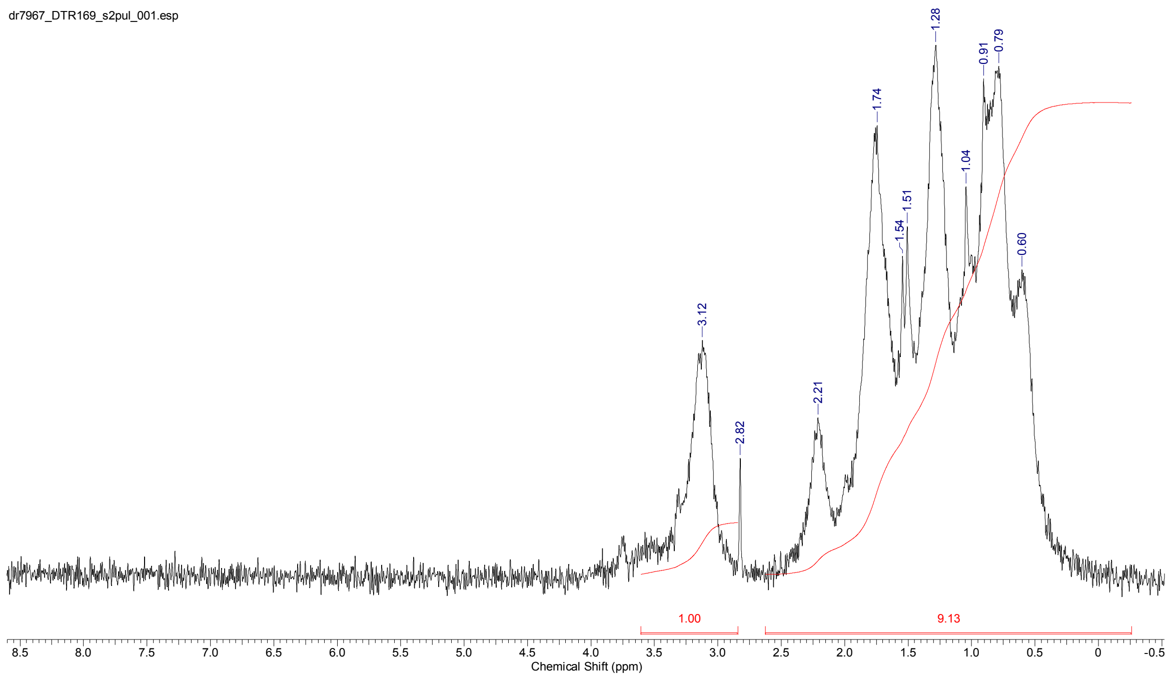

Figure S9. **^{2}H NMR (77 MHz, unlocked) spectrum of *trans*-2-(((*S*)-1-phenylethyl)amino)cyclohexanol-D_{10}, [D_{10}]-[*S*]-12**

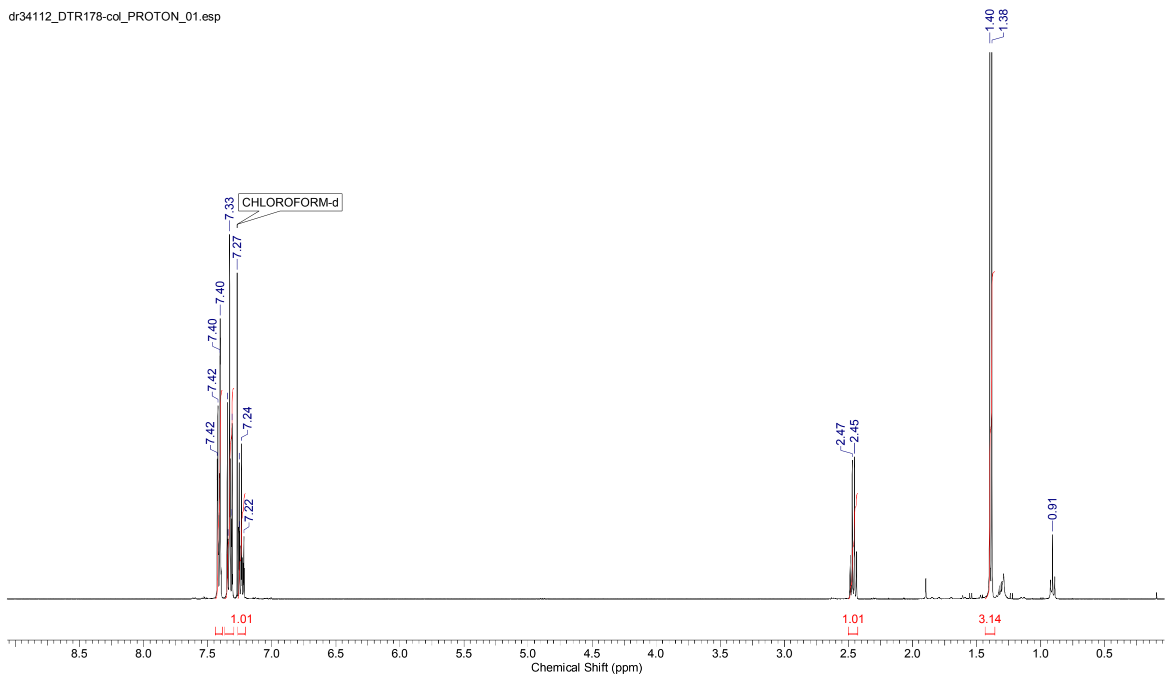

Figure S10. **^{1}H NMR (400 MHz, $CDCl_3$) spectrum of *N*-(*S*-α-phenylethyl)cyclohexene-D_{10} aziridine, [D_{10}]-[*S*]-13**

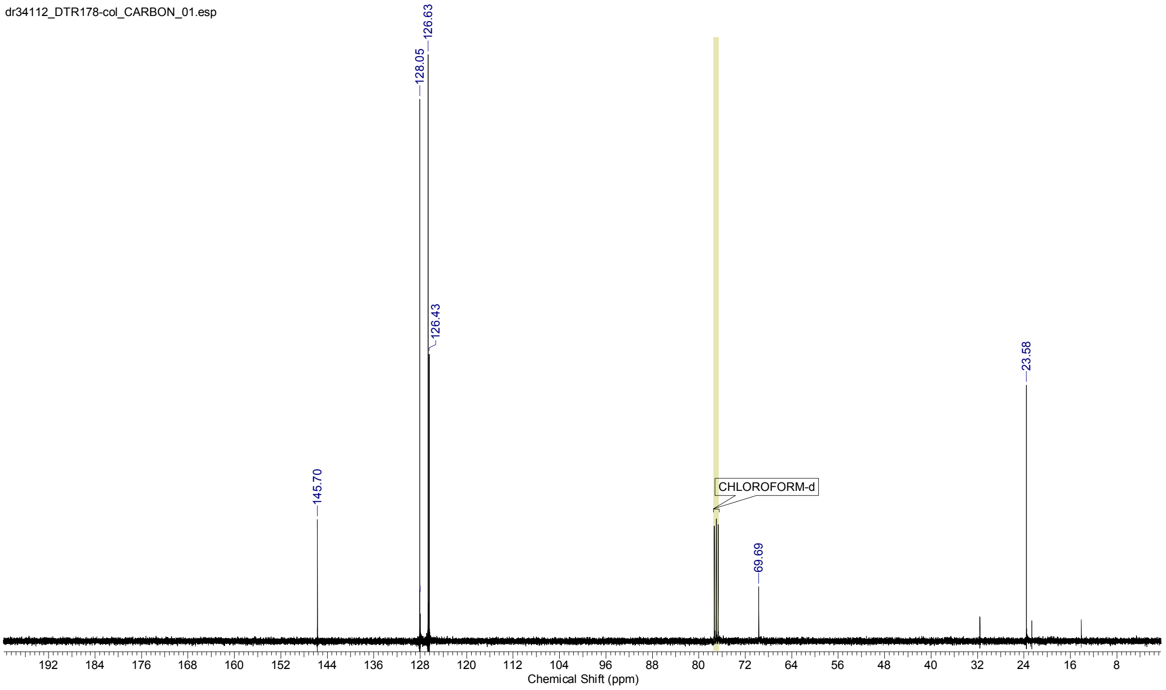

Figure S11. **^{13}C NMR (100 MHz, $CDCl_3$) spectrum of *N*-(*S*-α-phenylethyl)cyclohexene-D_{10} aziridine, [D_{10}]-[*S*]-13**

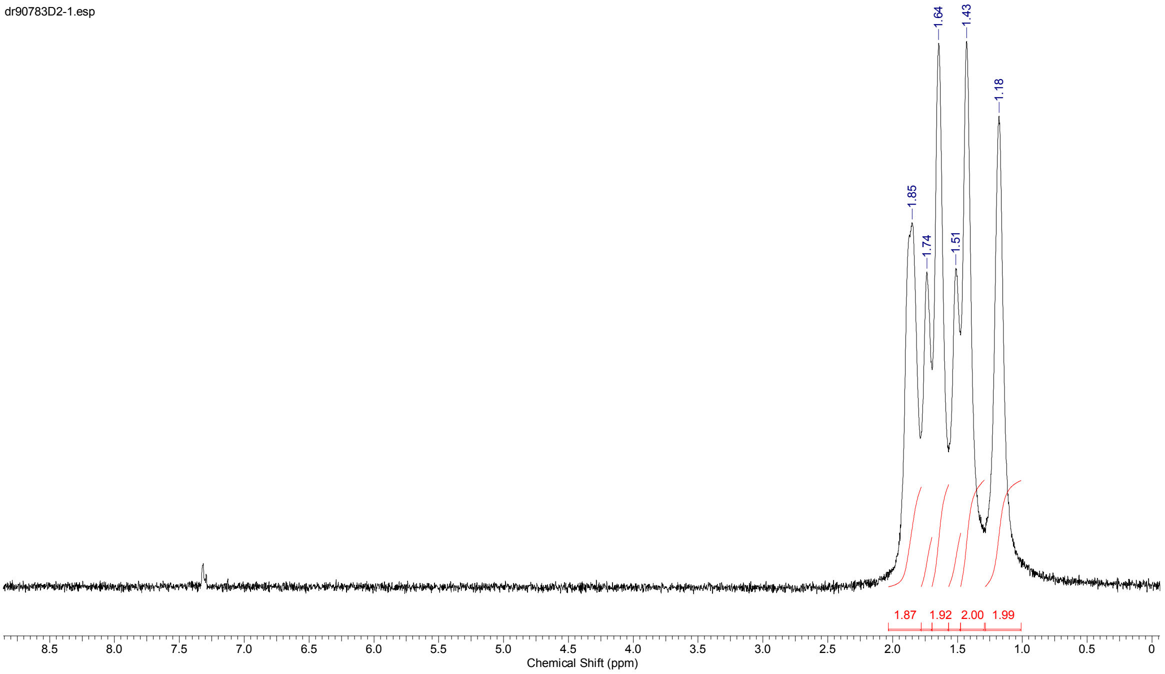

Figure S12. **^{2}H NMR (46 MHz, unlocked) spectrum of *N*-(*S*-α-phenylethyl)cyclohexene-D_{10} aziridine, [D_{10}]-[*S*]-13**

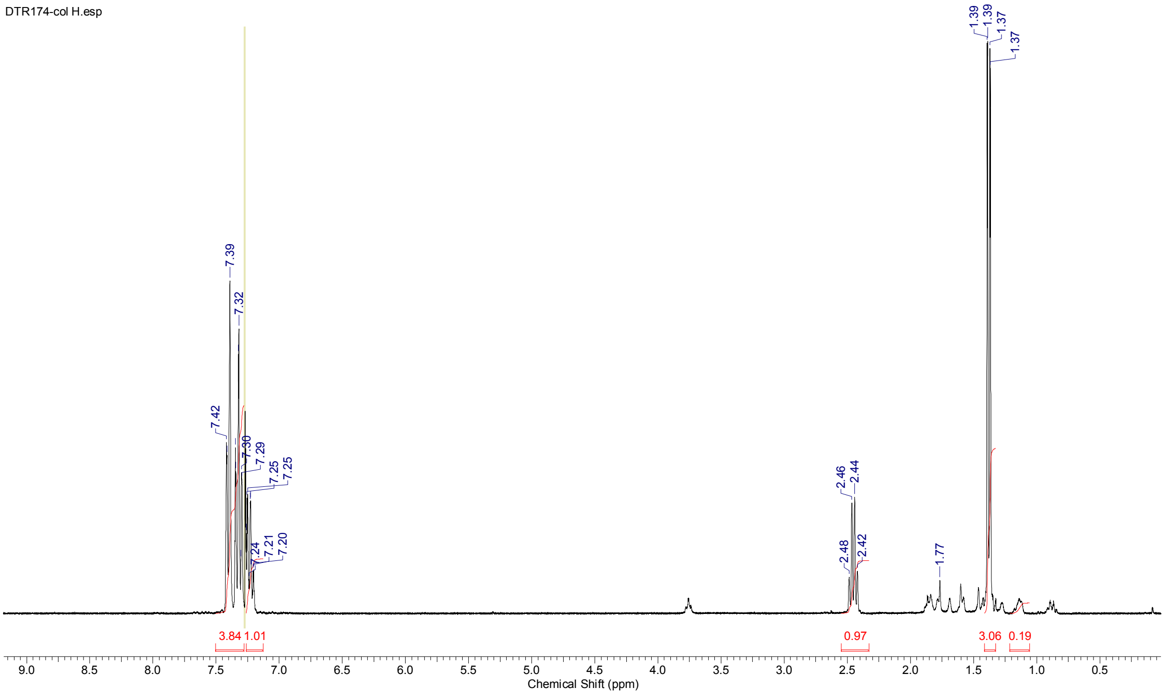

Figure S13. ^{1}H NMR (300 MHz, $CDCl_3$) spectrum of *N*-(*R*-α-phenylethyl)cyclohexene-D_{10} aziridine, [D_{10}]-[*R*]-13

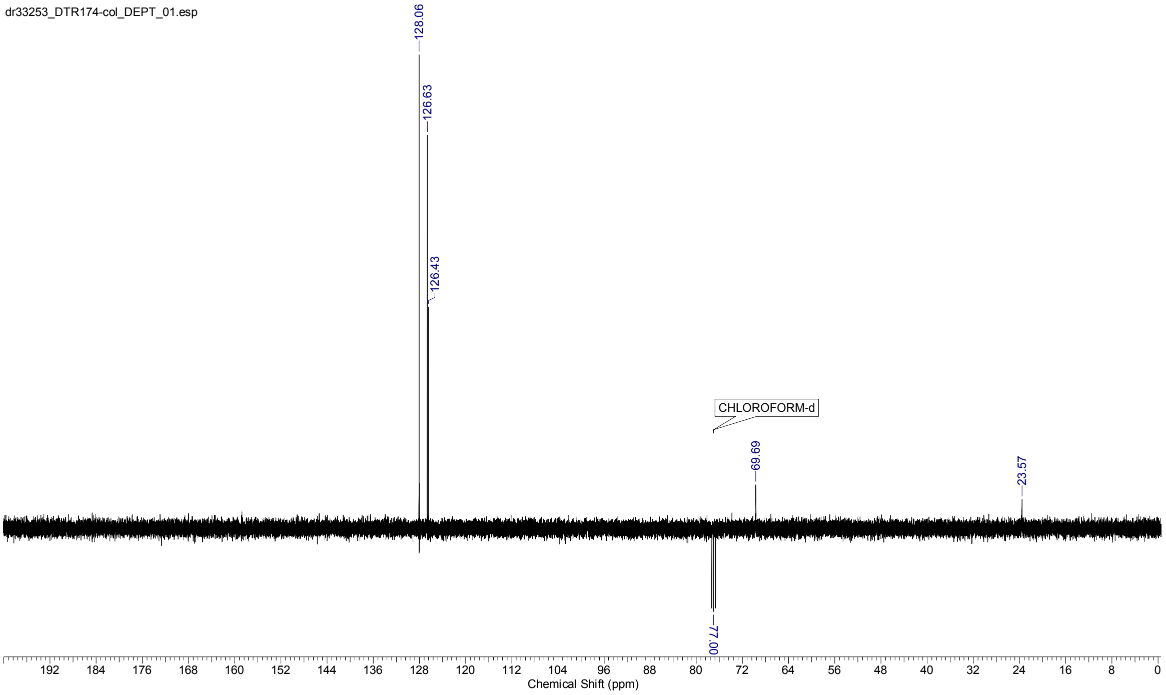

Figure S14. ^{13}C NMR (100 MHz, $CDCl_3$) spectrum of *N*-(*R*-α-phenylethyl)cyclohexene-D_{10} aziridine, [D_{10}]-[*R*]-13

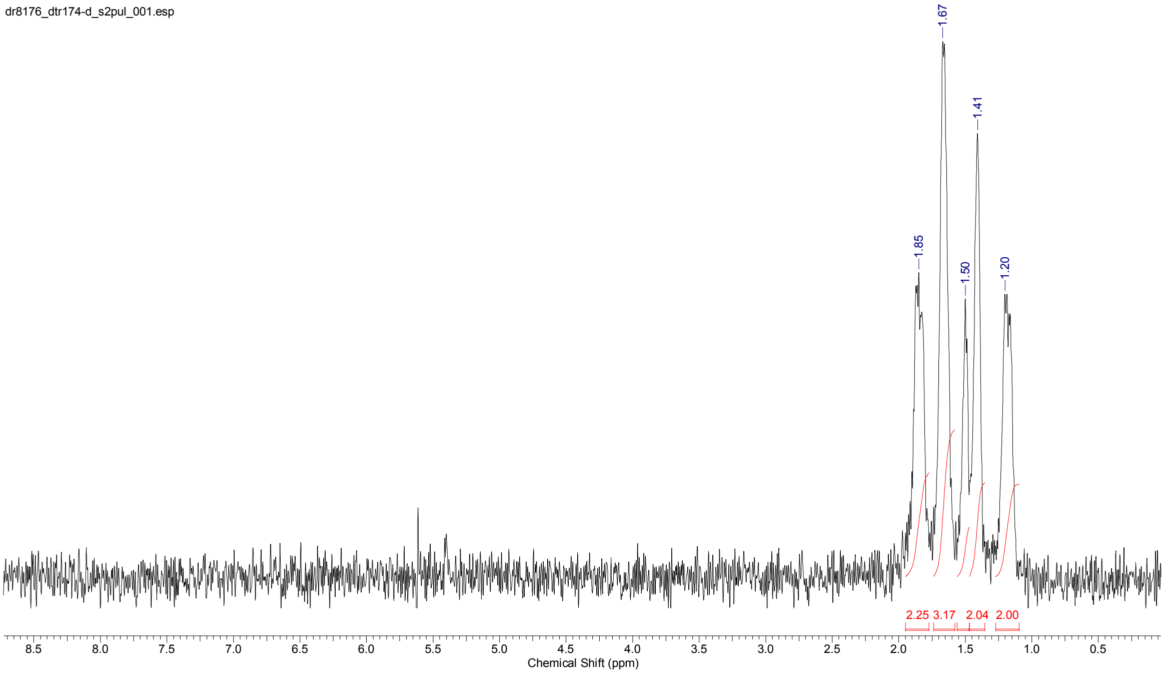

Figure S15. ^{2}**H NMR (77 MHz, unlocked) spectrum of** ***N*****-(*****R*****-α-phenylethyl)cyclohexene-D$_{10}$ aziridine, [D$_{10}$]-[*****R*****]-13**

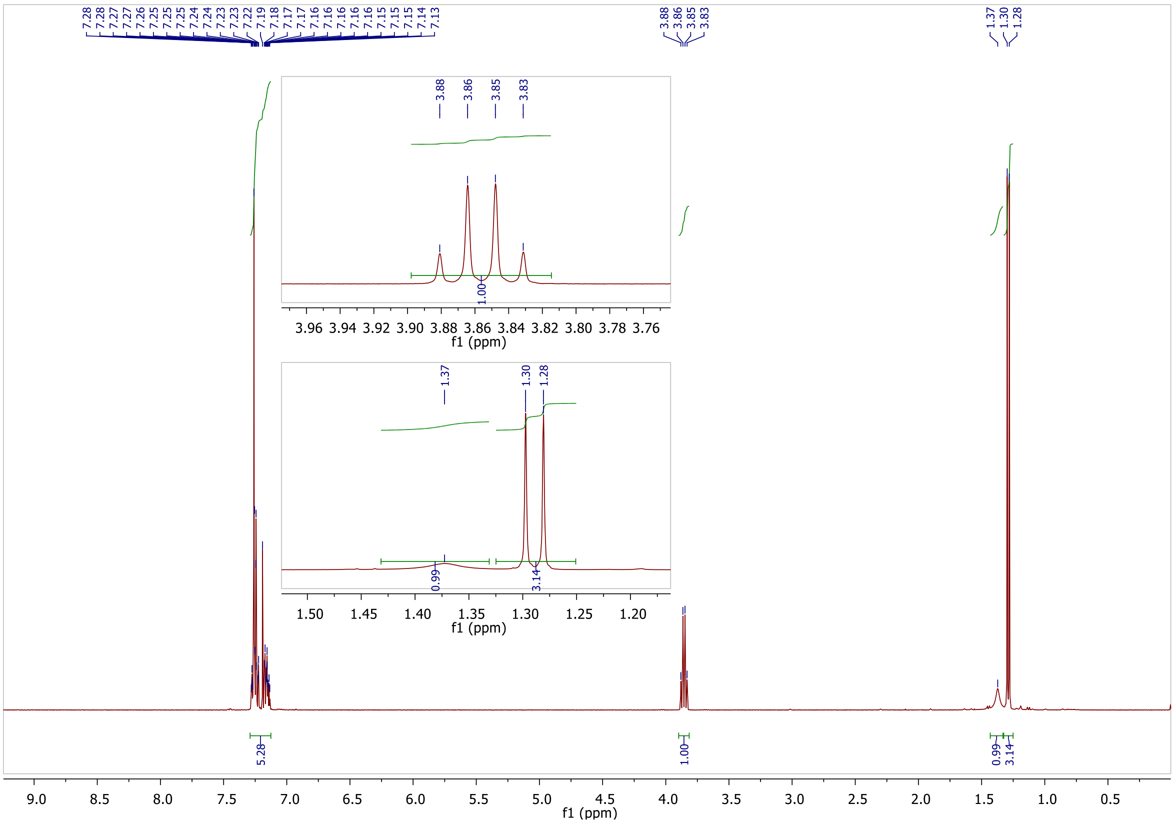

Figure S16. ^{1}**H NMR (400 MHz, CDCl$_3$) spectrum of** ***S,S*****-2-azido-*****N*****-(*****S*****-1-phenylethyl)-D$_{10}$-cyclohexanamine, [D$_{10}$]-(*****S*****,S,*****S*****)-14**

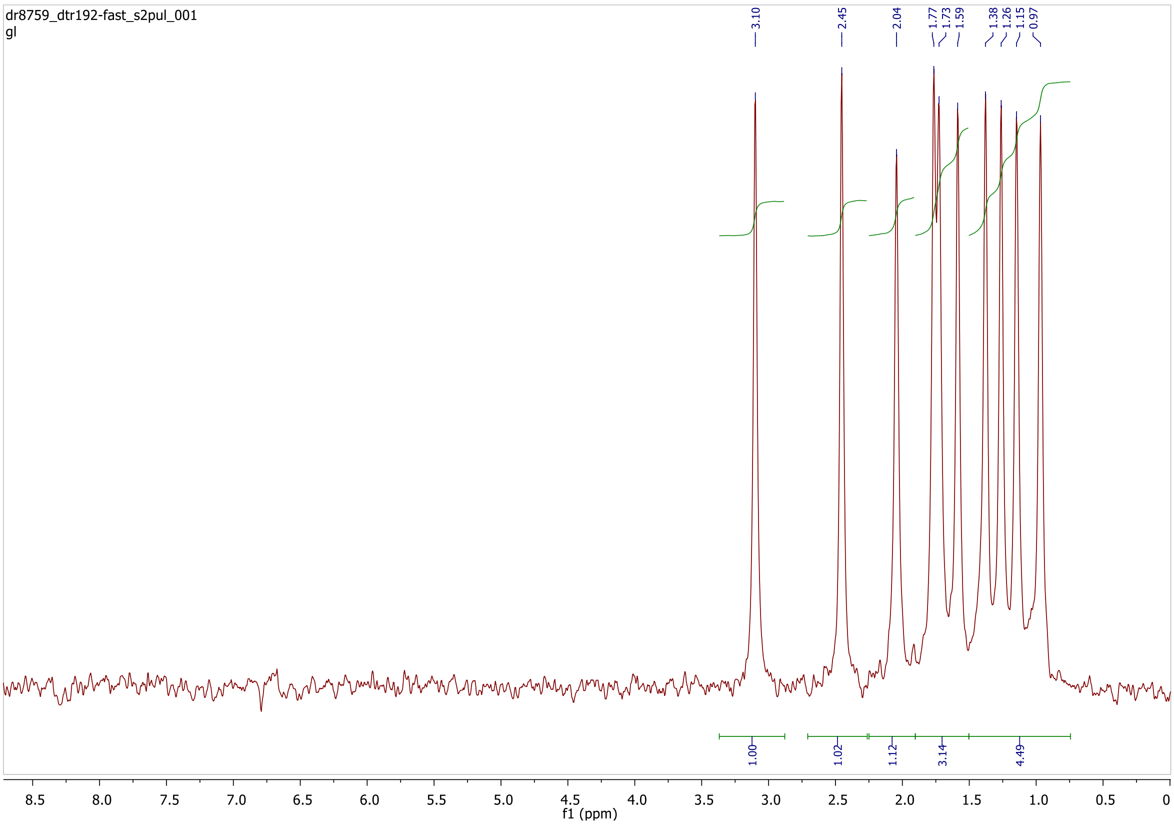

Figure S17. ^{2}**H NMR (77 MHz, unlocked) spectrum of *S*,*S*-2-azido-*N*-(*S*-1-phenylethyl)-D$_{10}$-cyclohexanamine, [D$_{10}$]-(*S*,S,*S*)-14**

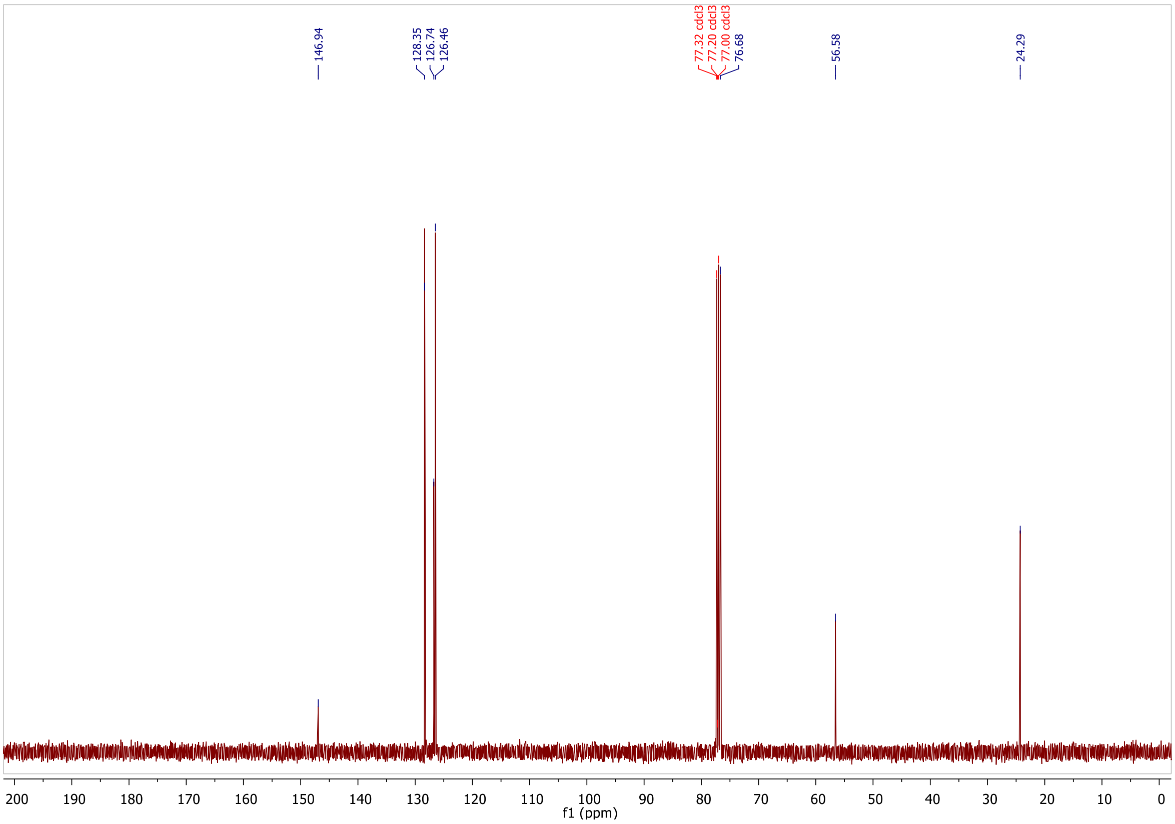

Figure S18. ^{13}**C NMR (101 MHz, CDCl$_3$) spectrum of *S*,*S*-2-azido-*N*-(*S*-1-phenylethyl)-D$_{10}$-cyclohexanamine, [D$_{10}$]-(*S*,S,*S*)-14**

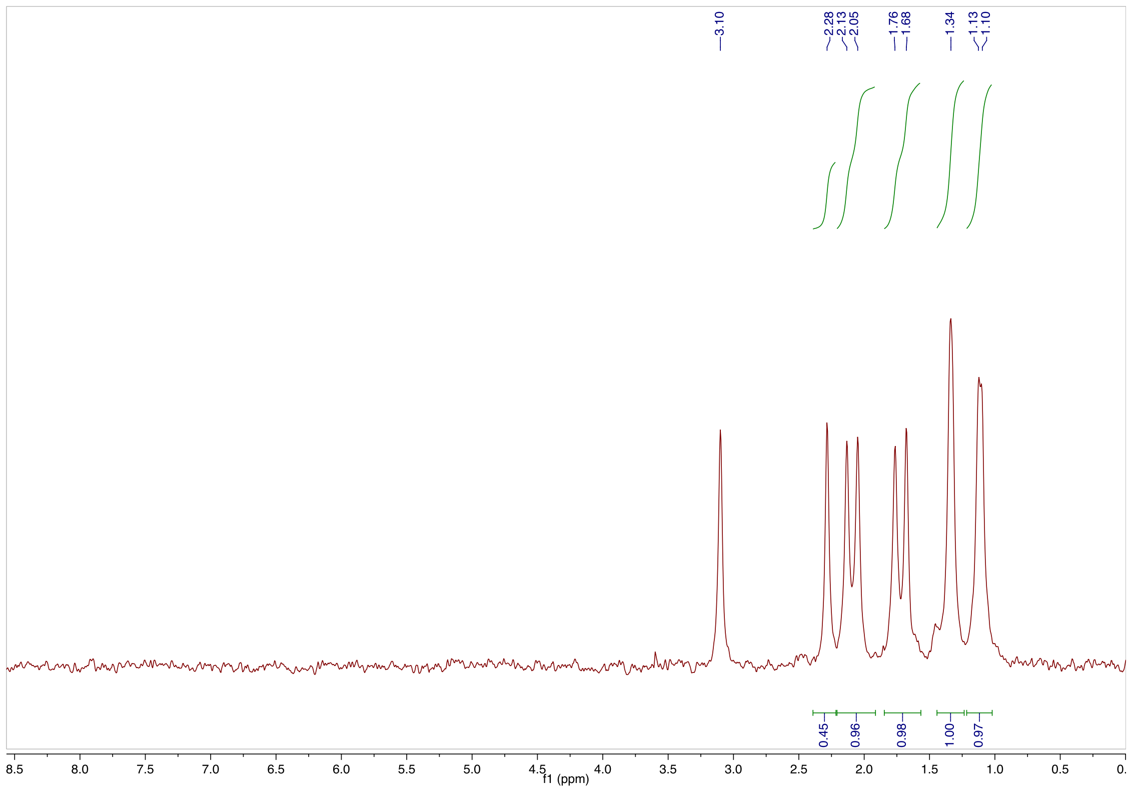

Figure S19. ^{2}**H NMR (77 MHz, unlocked) spectrum of *S,S*-2-azido-*N*-(*R'*-1-phenylethyl)-D_{10}-cyclohexanamine, [D_{10}]-(*S*,S,*R'*)-14**

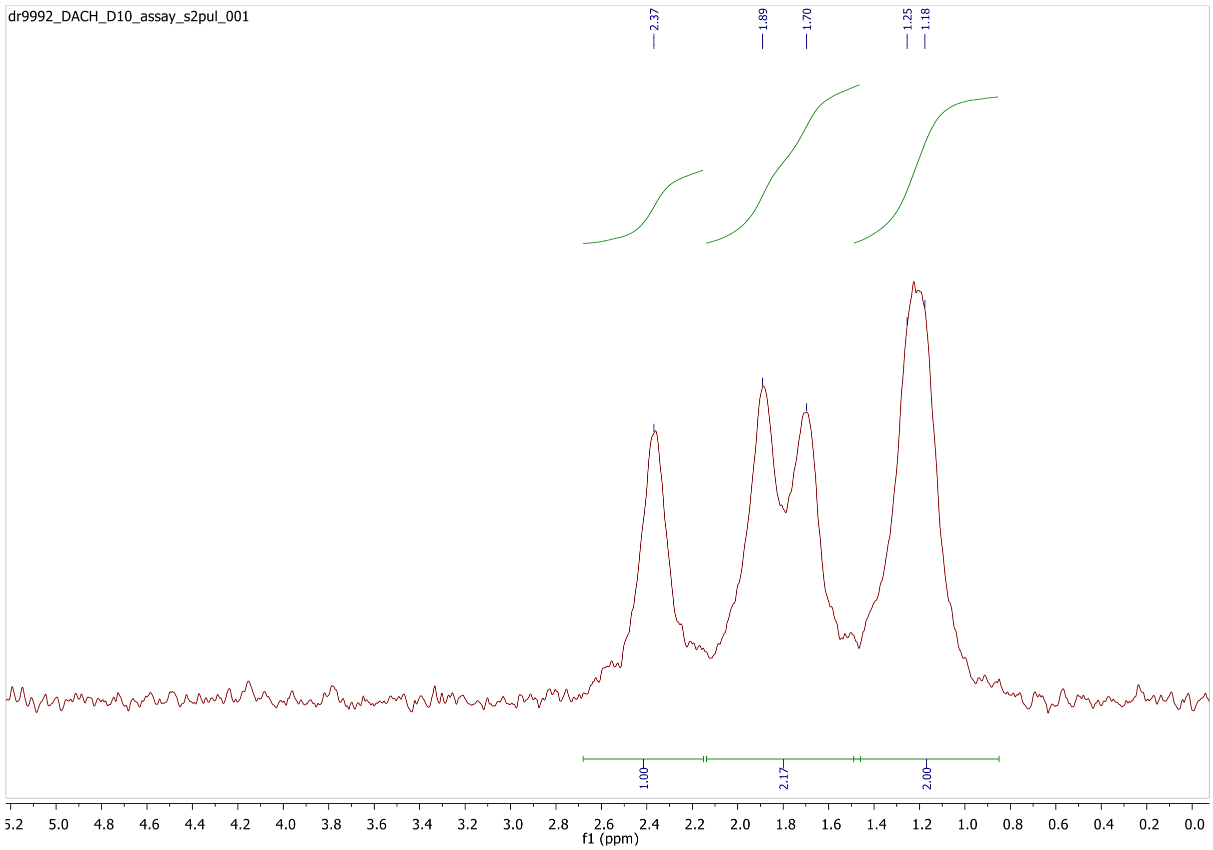

Figure S20. ^{2}**H NMR (77 MHz, unlocked) spectrum of (1*S*,2*S*)-diaminocyclohexane-[D_{10}], [D_{10}]-(*S*,*S*)-15**

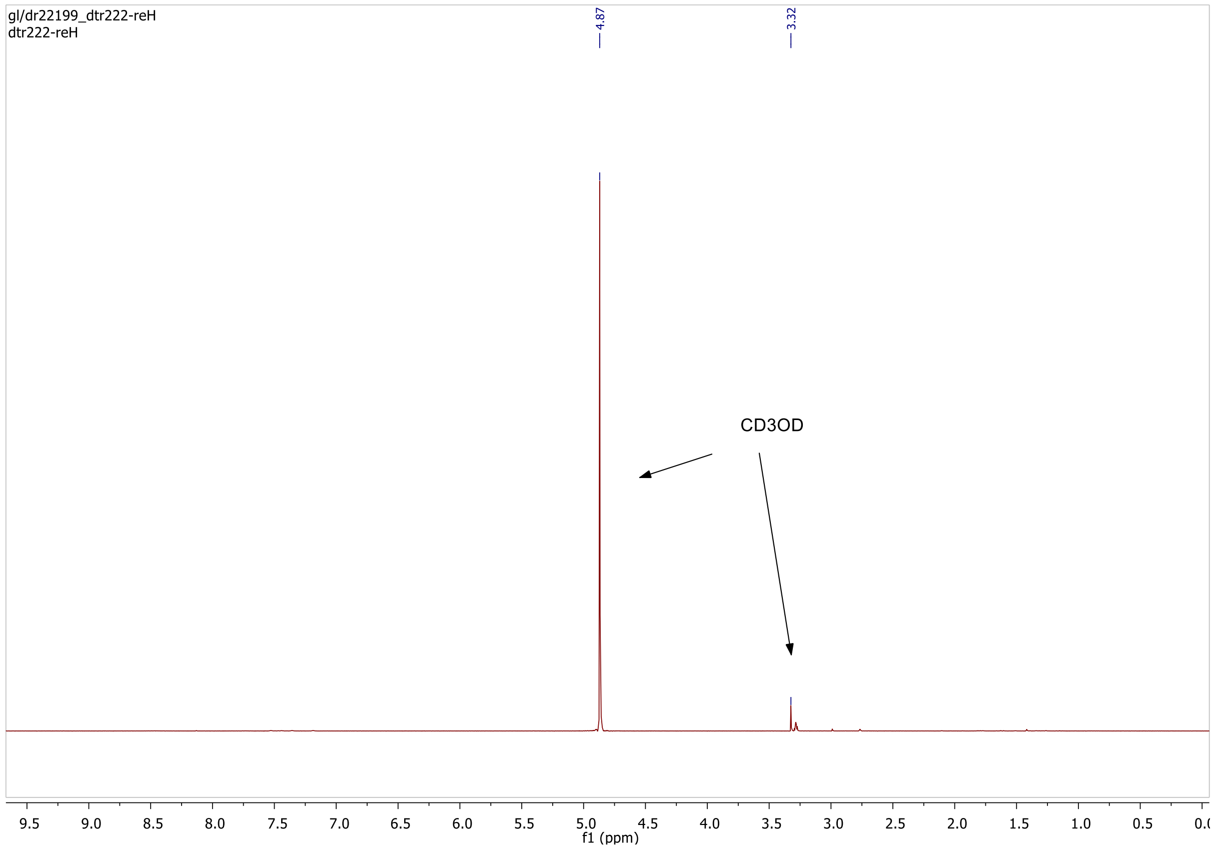

Figure S21. ^{1}**H NMR (301 MHz,** CD_3OD**) spectrum of (1*S*,2*S*)-diaminocyclohexane-[**D_{10}**], [**D_{10}**]-(*S*,*S*)-15**

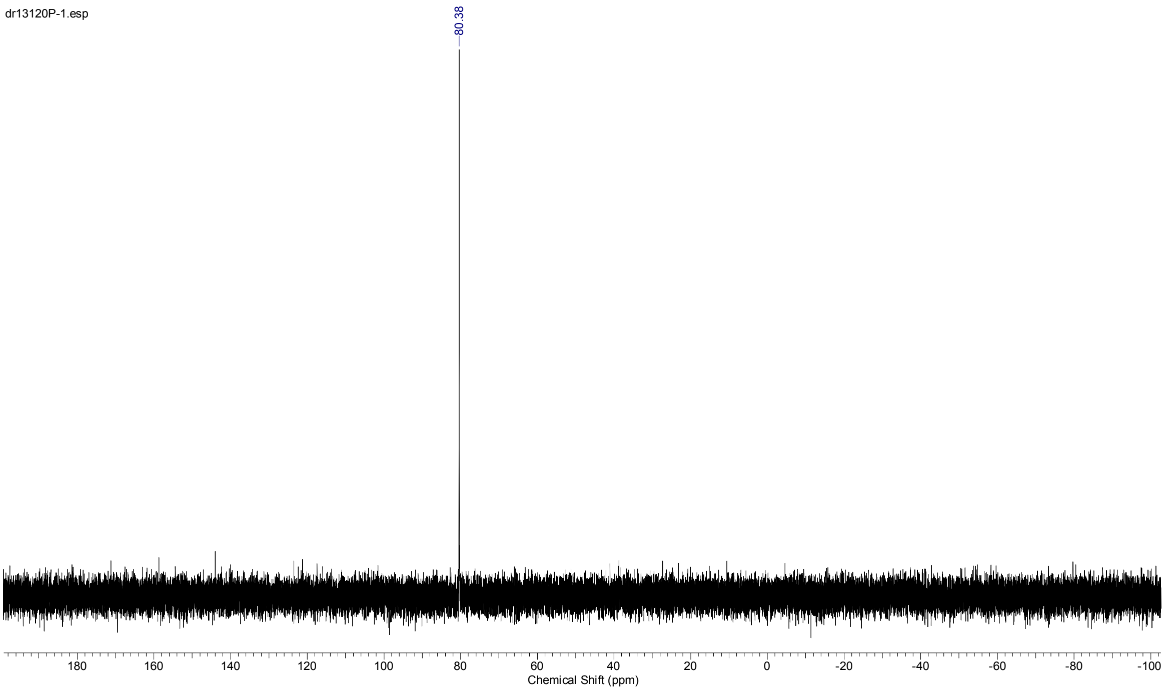

Figure S22. ^{31}**P NMR (122 MHz, unlocked) spectrum of chlorodiphenylphosphine-**D_{10}**, [**D_{10}**]-8**

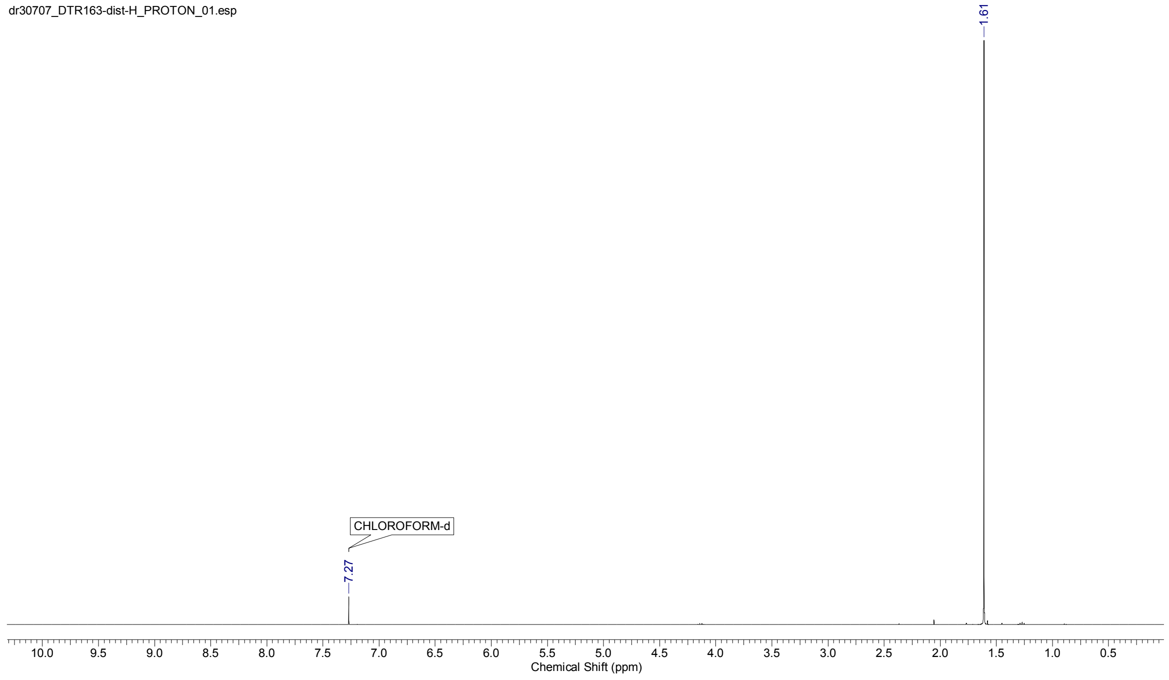

Figure S23. ^{1}**H NMR (400 MHz, CDCl$_3$) spectrum of *tert*-butyl benzoate-D$_5$, [D$_5$]-6**

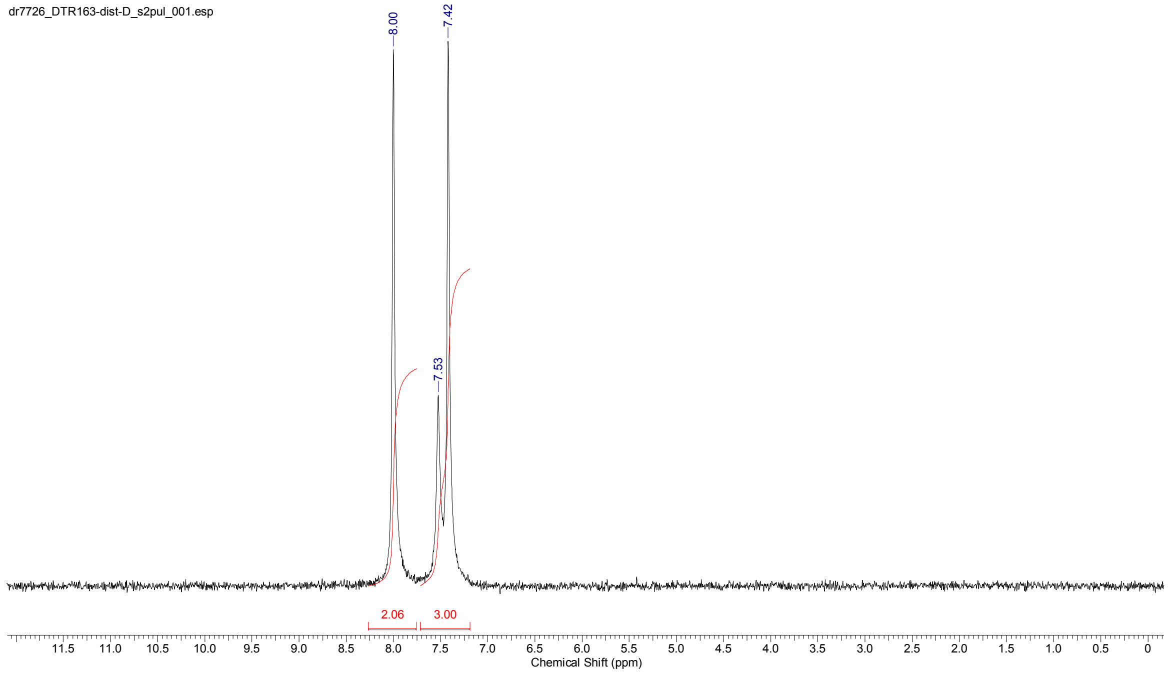

Figure S24. ^{2}**H NMR (77 MHz, unlocked) spectrum of *tert*-butyl benzoate-D$_5$, [D$_5$]-6**

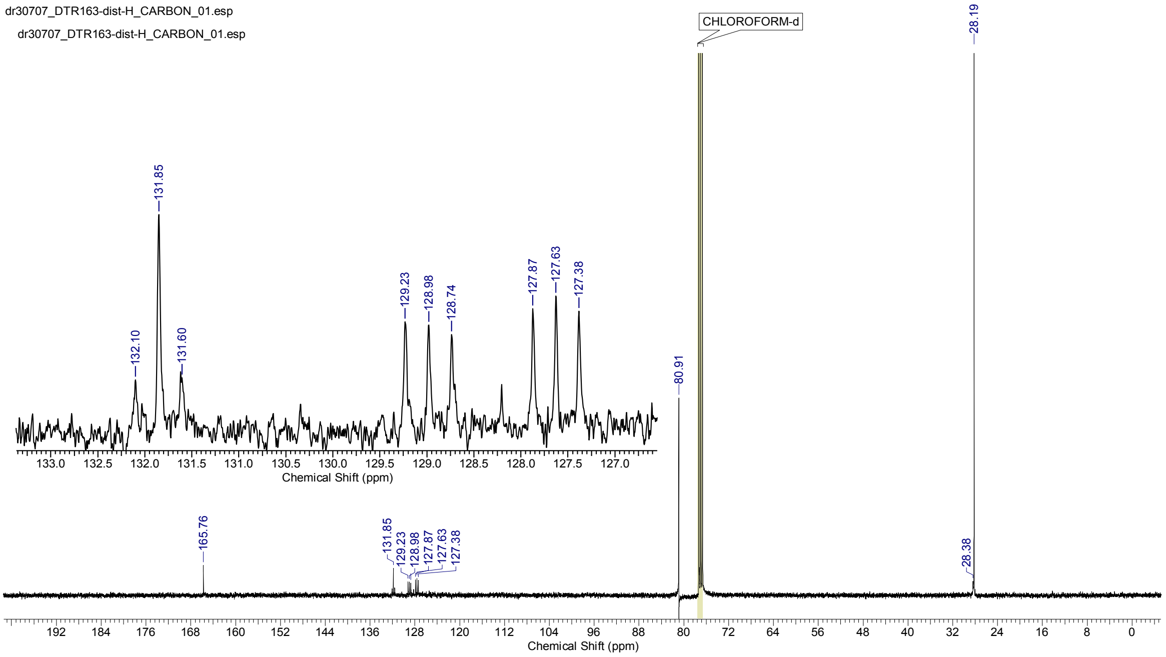

Figure S25. ^{13}C **NMR (100 MHz,** $CDCl_3$**) spectrum of** ***tert*****-butyl benzoate-D$_5$, [D$_5$]-6**

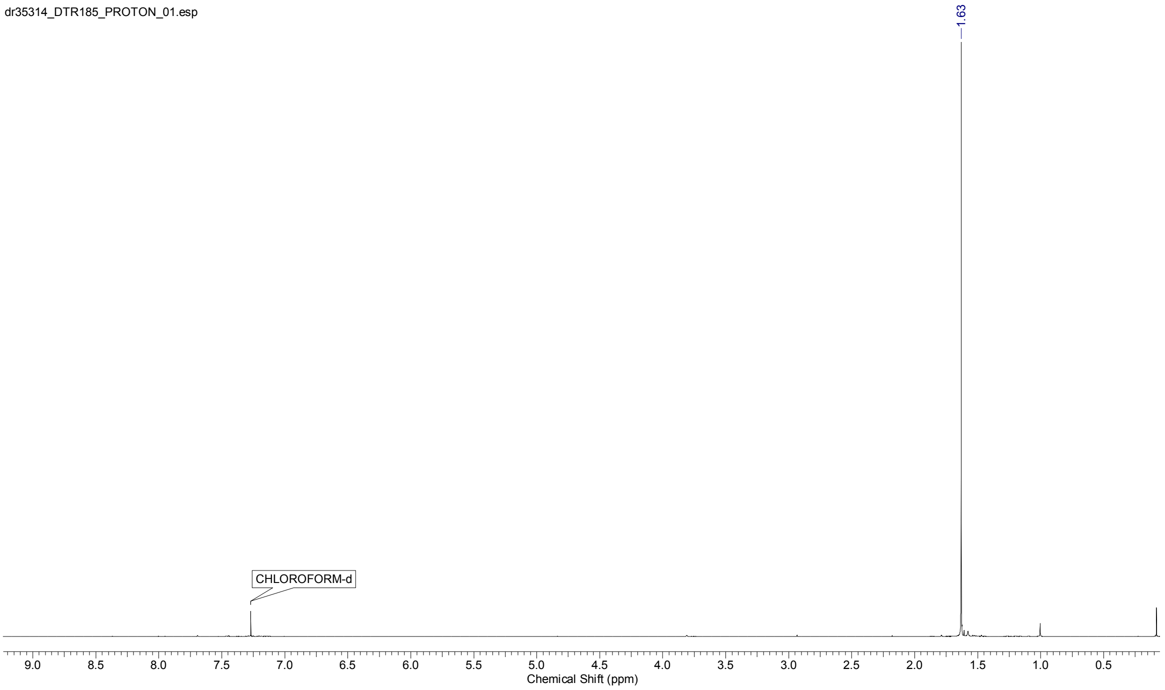

Figure S26. ^{1}H **NMR (400 MHz,** $CDCl_3$**) spectrum of** ***tert*****-butyl 2-iodobenzoate-D$_4$, [D$_4$]-7**

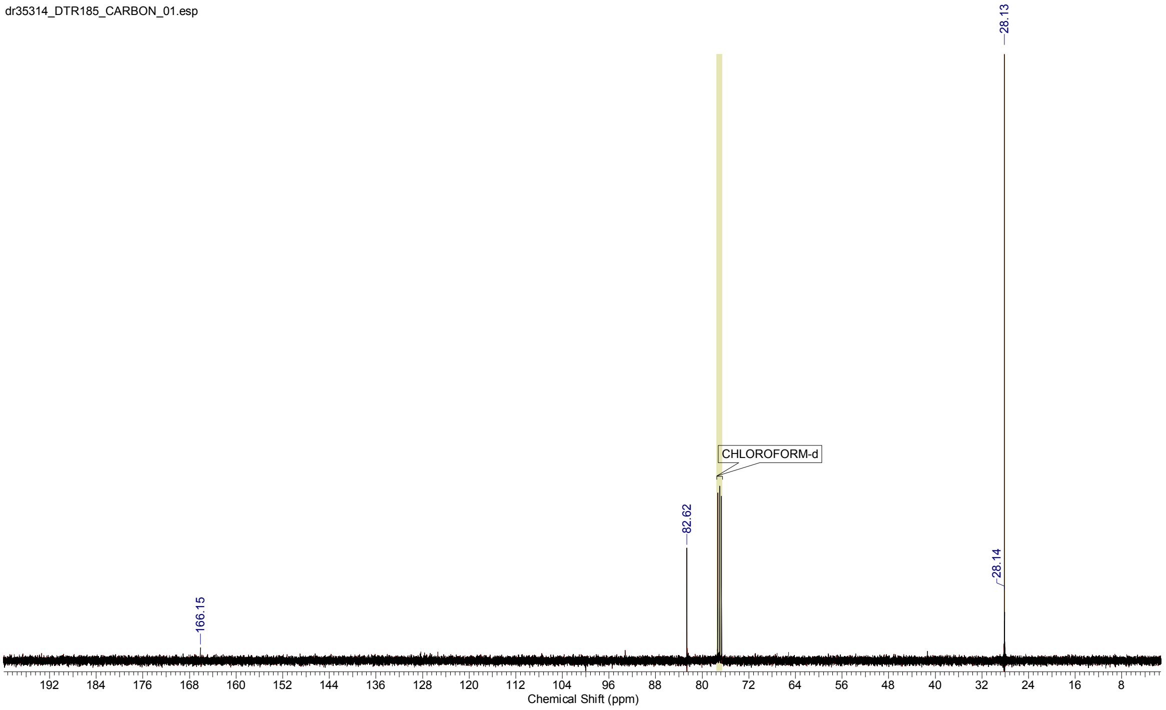

Figure S27. ^{13}**C NMR (100 MHz, CDCl$_3$) spectrum of *tert*-butyl 2-iodobenzoate-D$_4$, [D$_4$]-7**

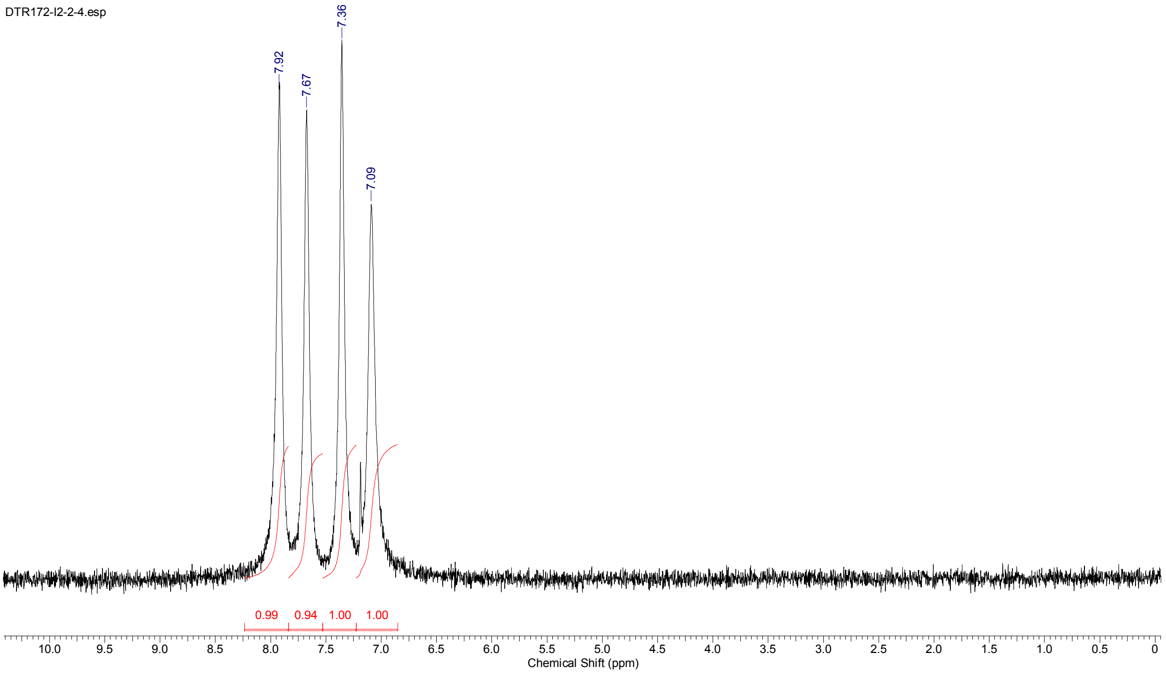

Figure S28. ^{2}**H NMR (46 MHz, unlocked) spectrum of *tert*-butyl 2-iodobenzoate-D$_4$, [D$_4$]-7**

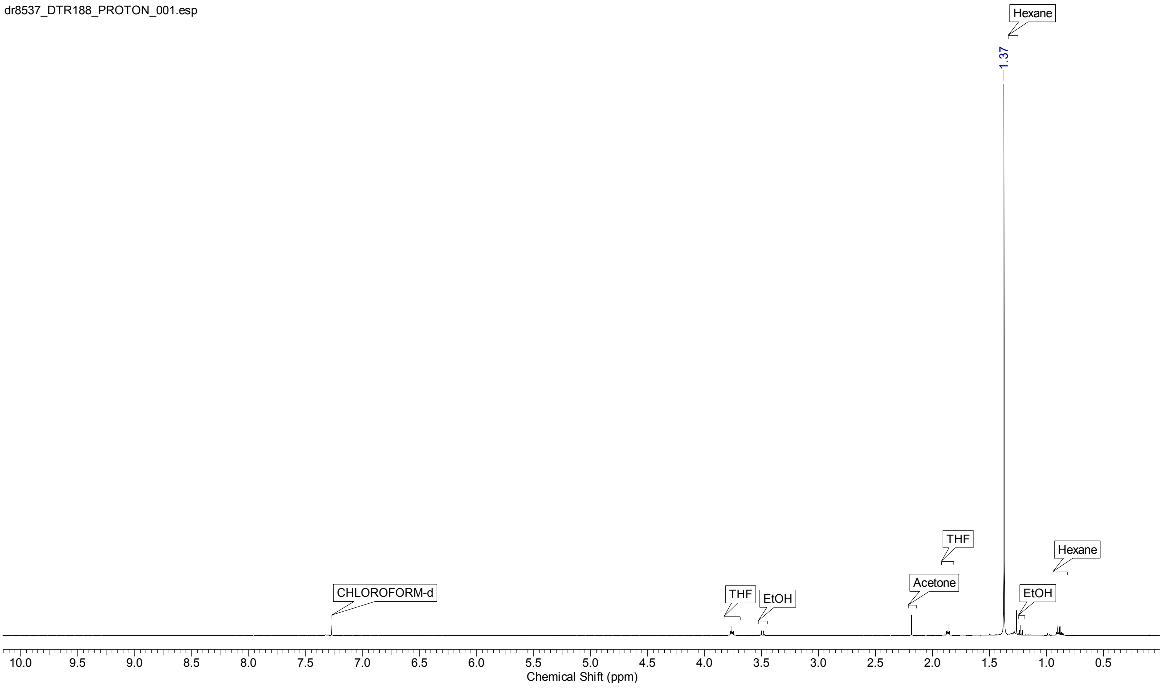

Figure S29. ^{1}**H NMR (500 MHz, CDCl$_3$) spectrum of *tert*-butyl D$_{14}$-2-(diphenylphosphino)benzoate, [D$_{14}$]-9**

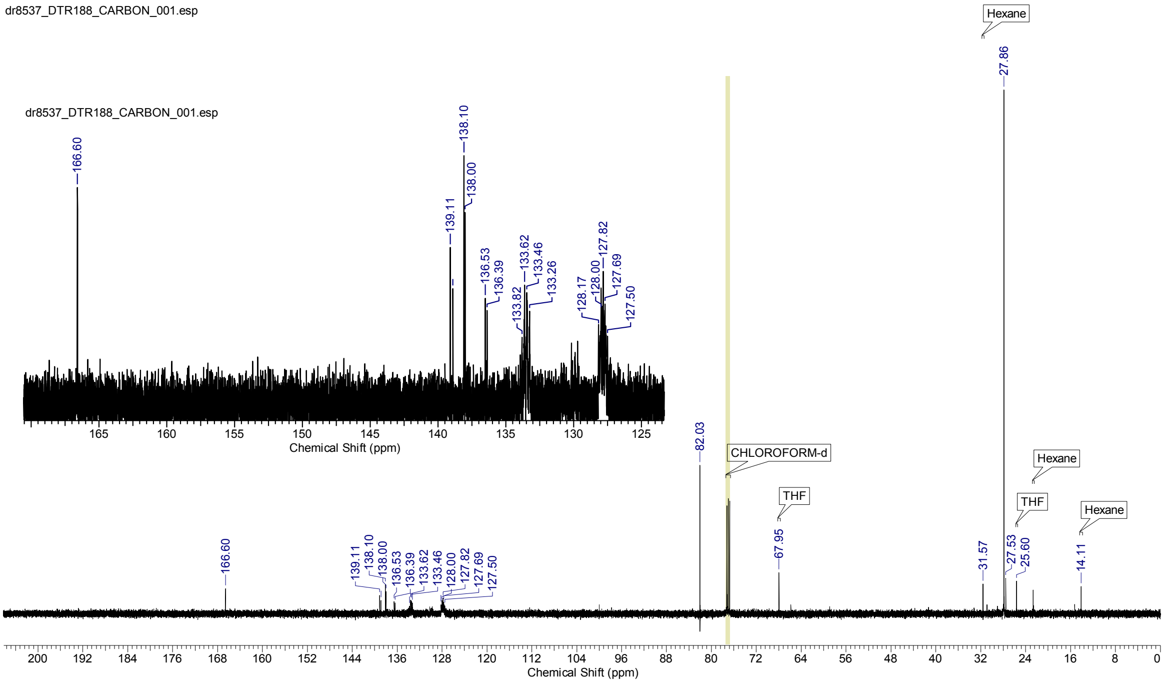

Figure S30. ^{13}**C NMR (126 MHz, CDCl$_3$) spectrum of *tert*-butyl D$_{14}$-2-(diphenylphosphino)benzoate, [D$_{14}$]-9**

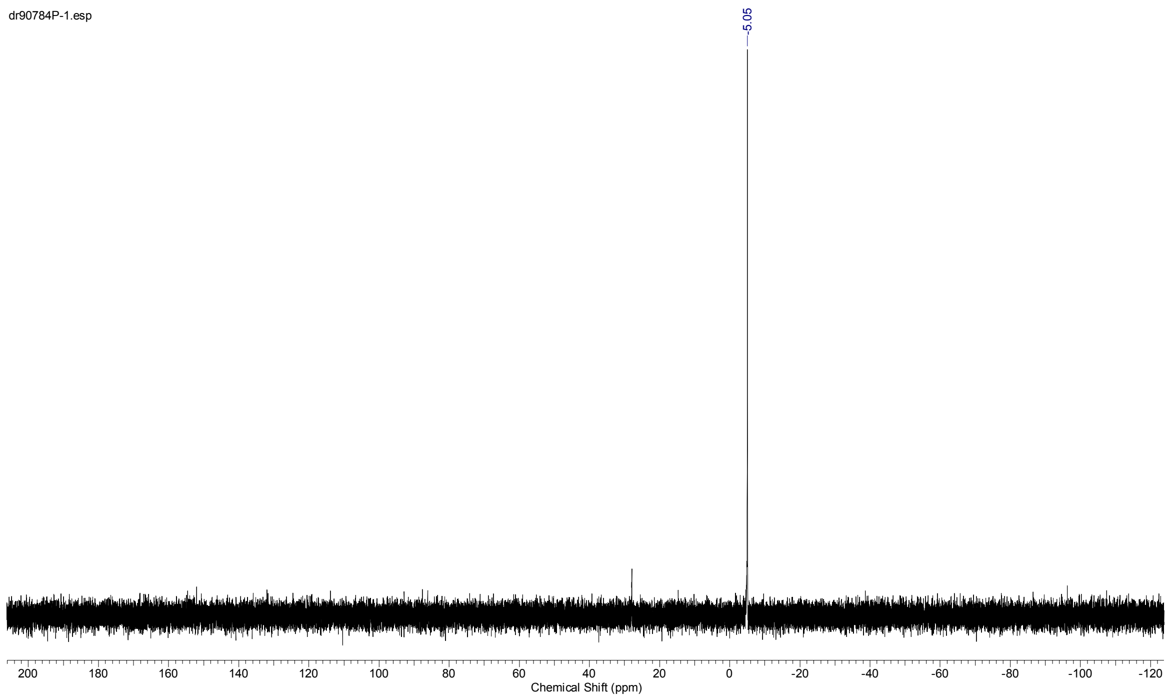

Figure S31. ^{31}**P NMR (121 MHz, CDCl$_3$) spectrum of *tert*-butyl D$_{14}$-2-(diphenylphosphino)benzoate, [D$_{14}$]-9**

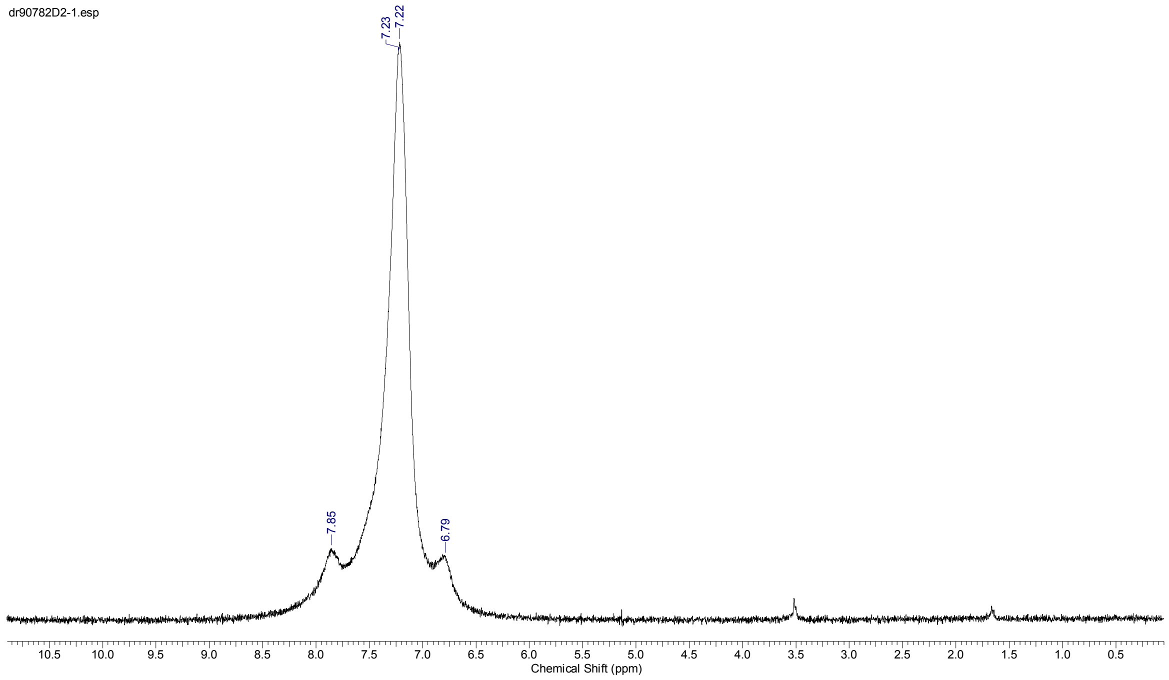

Figure 32. ^{2}**H NMR (46 MHz, unlocked) spectrum of *tert*-butyl D$_{14}$-2-(diphenylphosphino)benzoate, [D$_{14}$]-9**

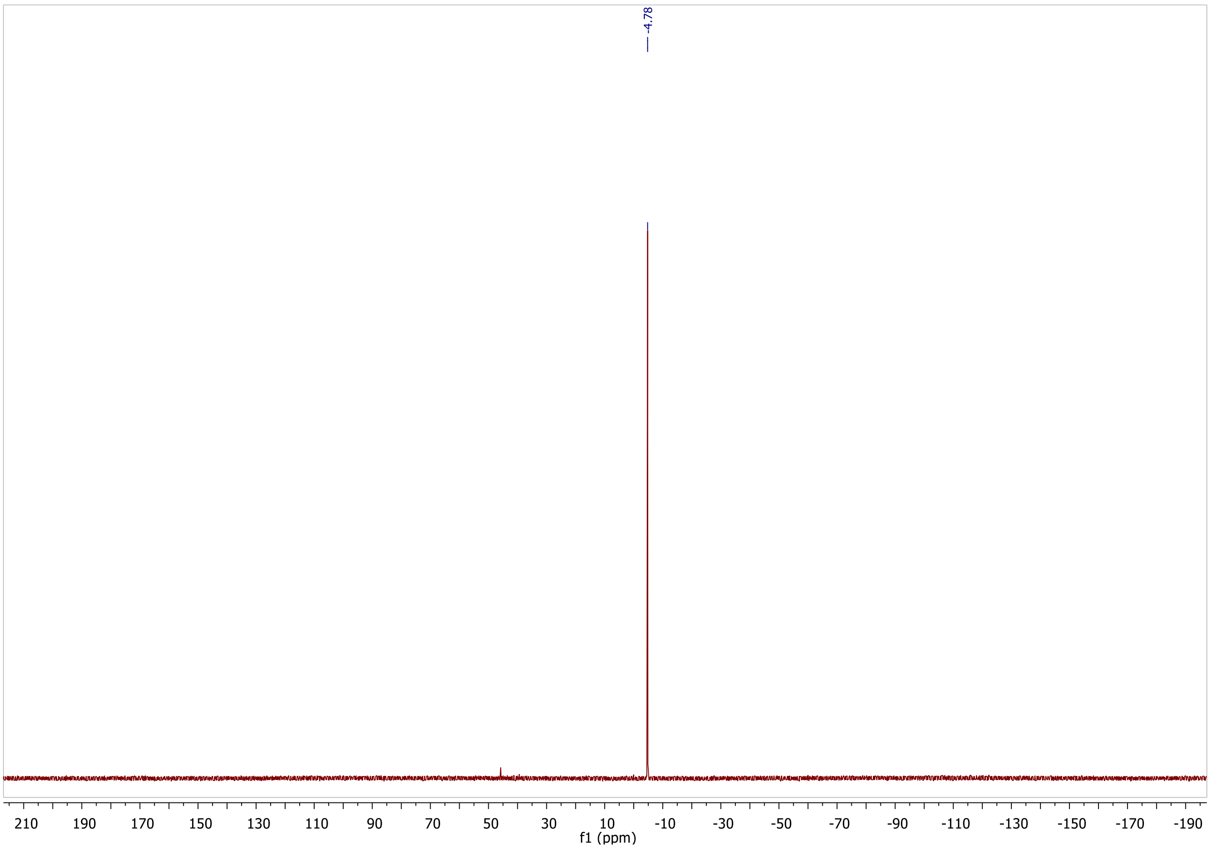

Figure S33. ^{31}P NMR (202 MHz, $CDCl_3$) spectrum of D_{14}-2-(diphenylphosphino)benzoic acid, [D_{14}]-10

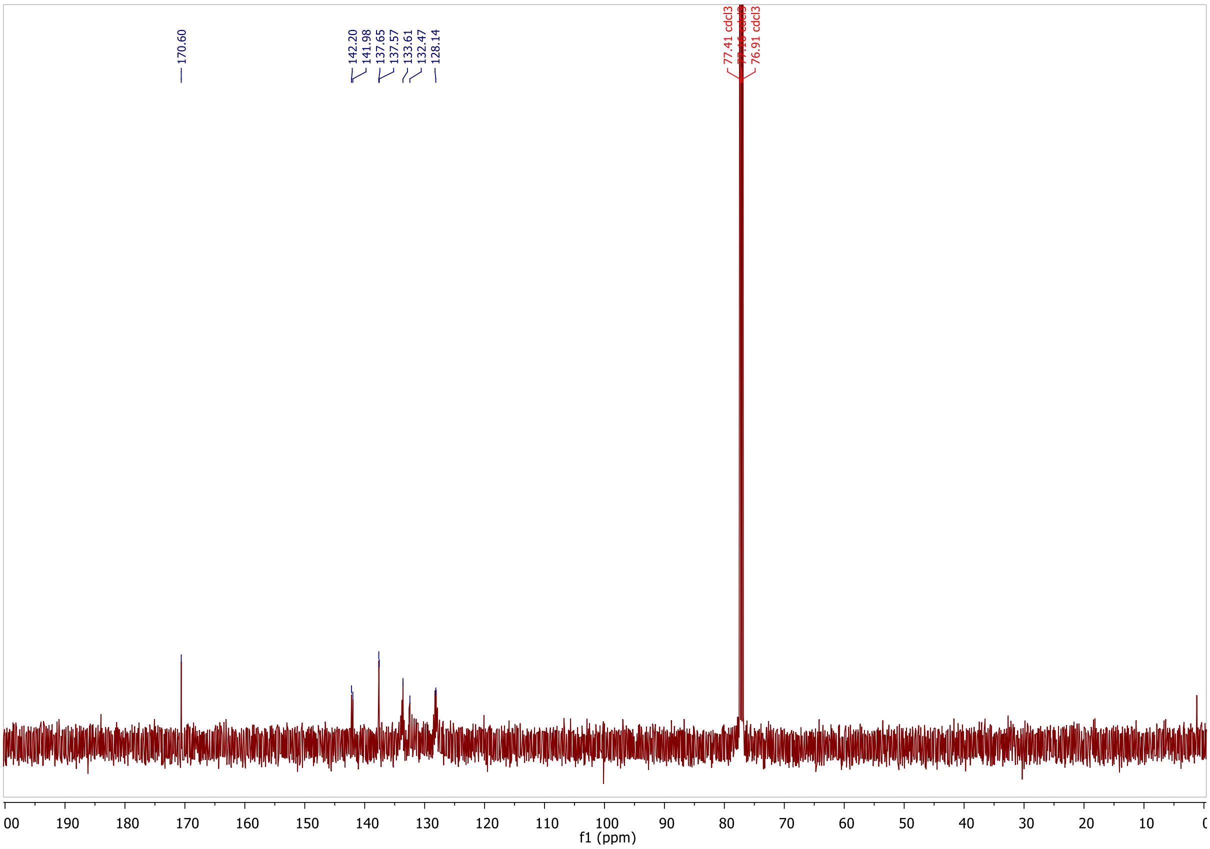

Figure S34. ^{13}C NMR (126 MHz, $CDCl_3$) spectrum of D_{14}-2-(diphenylphosphino)benzoic acid, [D_{14}]-10

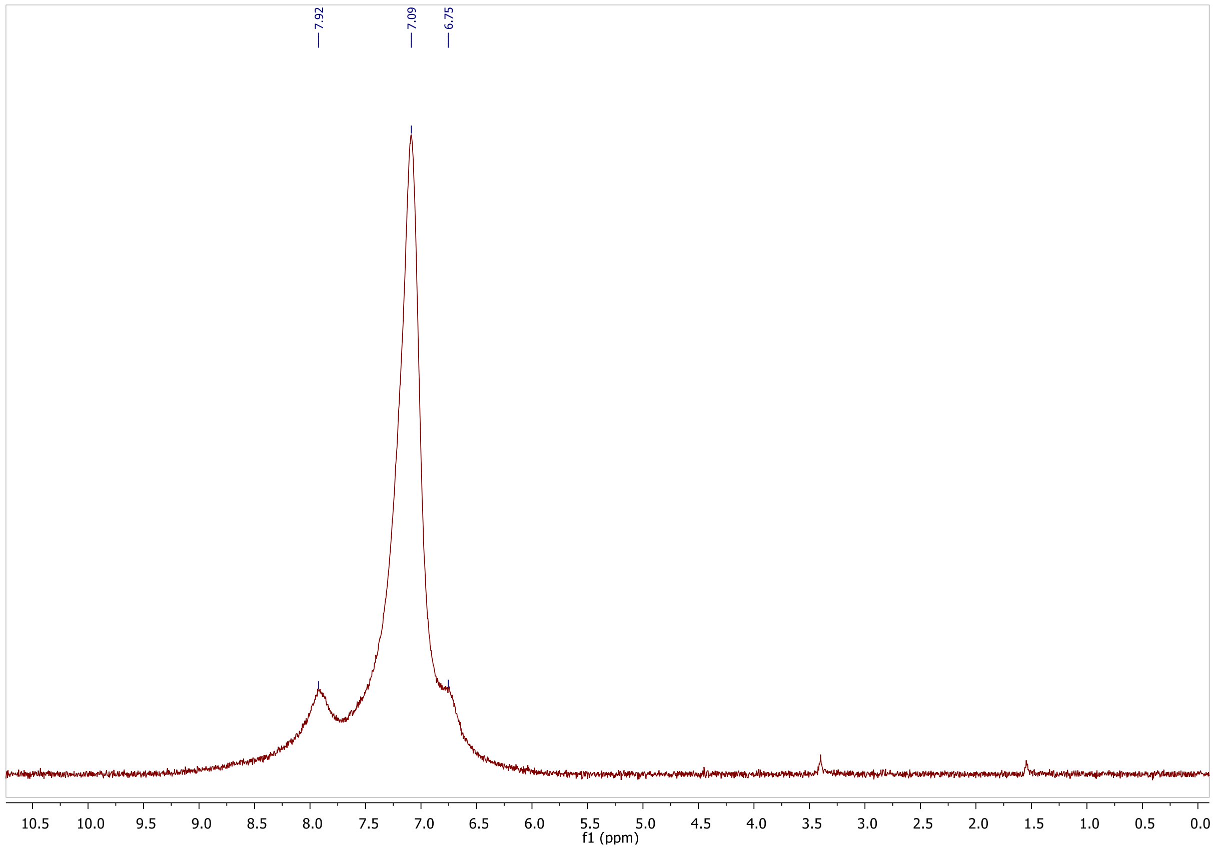

Figure S35. ^{2}**H NMR (46 MHz, unlocked) spectrum of** D_{14}**-2-(diphenylphosphino)benzoic acid, [**D_{14}**]-10**

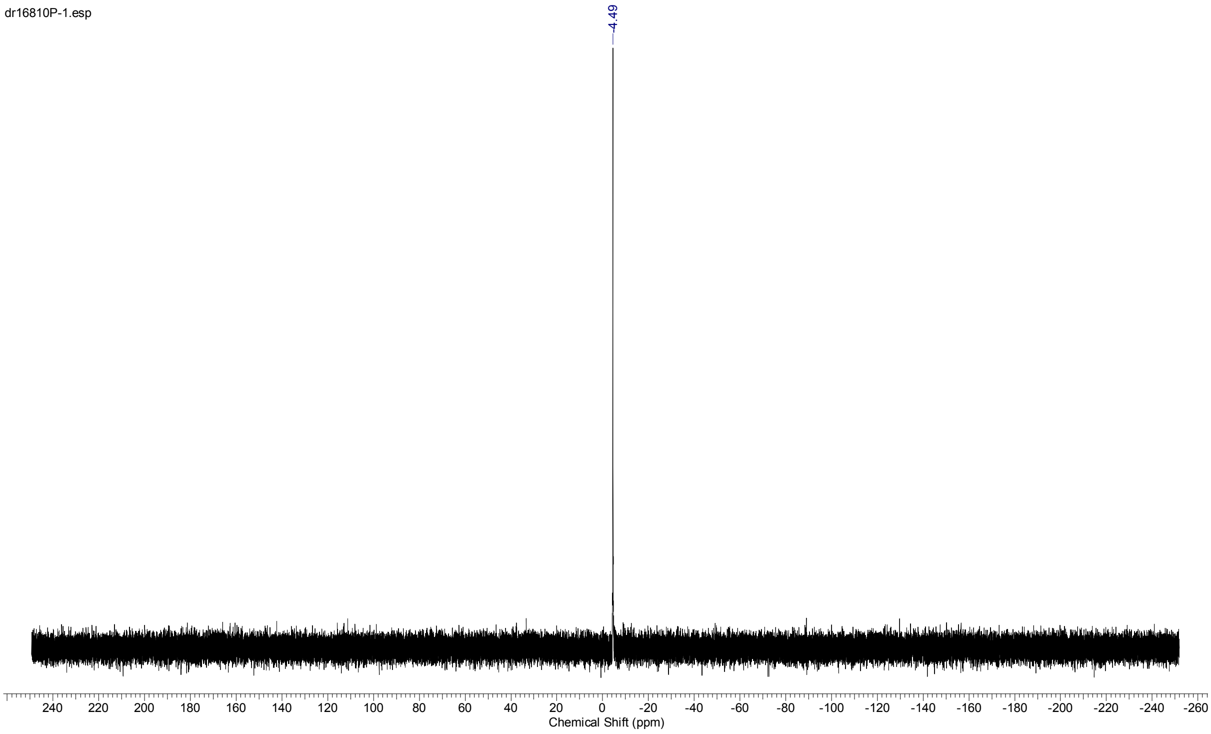

Figure S36. ^{31}**P NMR (122 MHz, unlocked) spectrum of** D_4**-2-(diphenylphosphino)benzoic acid, [**D_4**]-10**

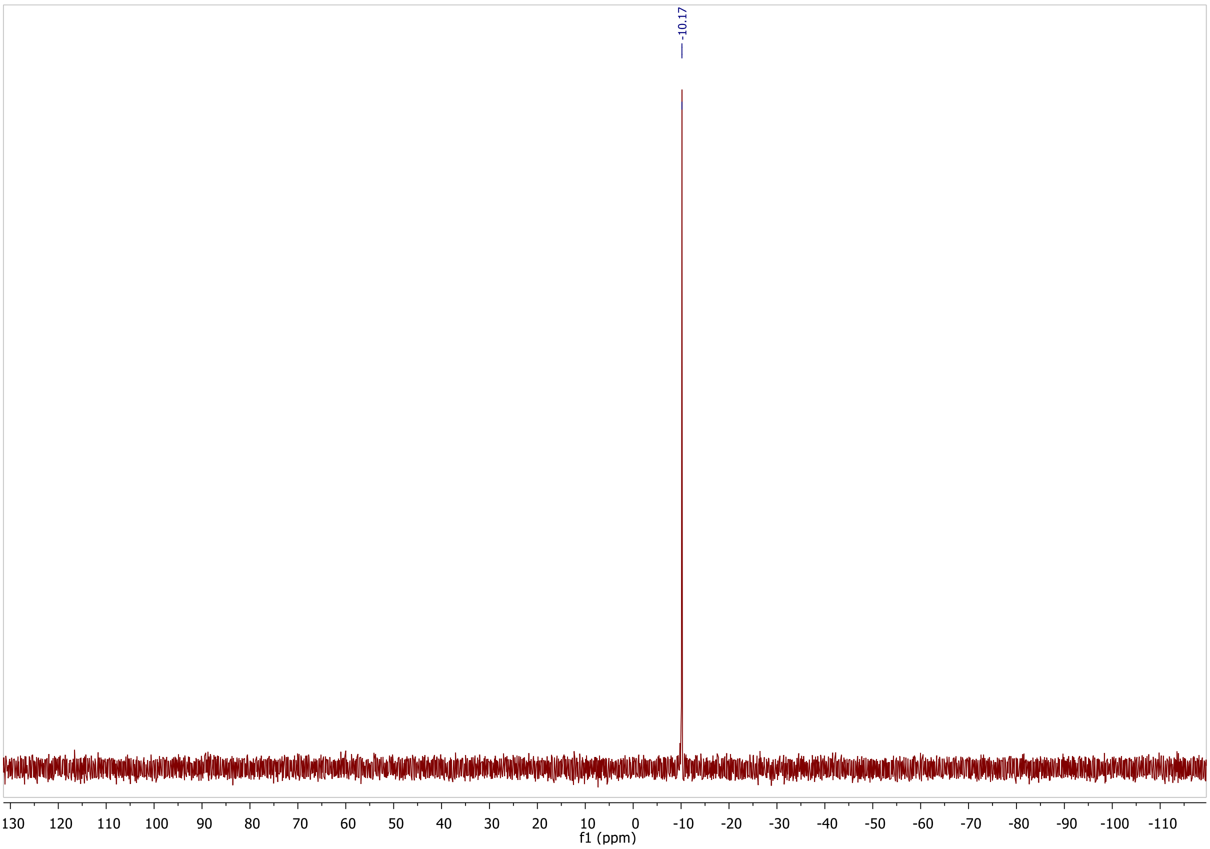

Figure S37. ^{31}P **NMR (121 MHz, unlocked) spectrum of** D_{38}**-*S*,*S*-DACH-phenyl Trost Ligand, [**D_{38}**]-(*S*,*S*)-2**

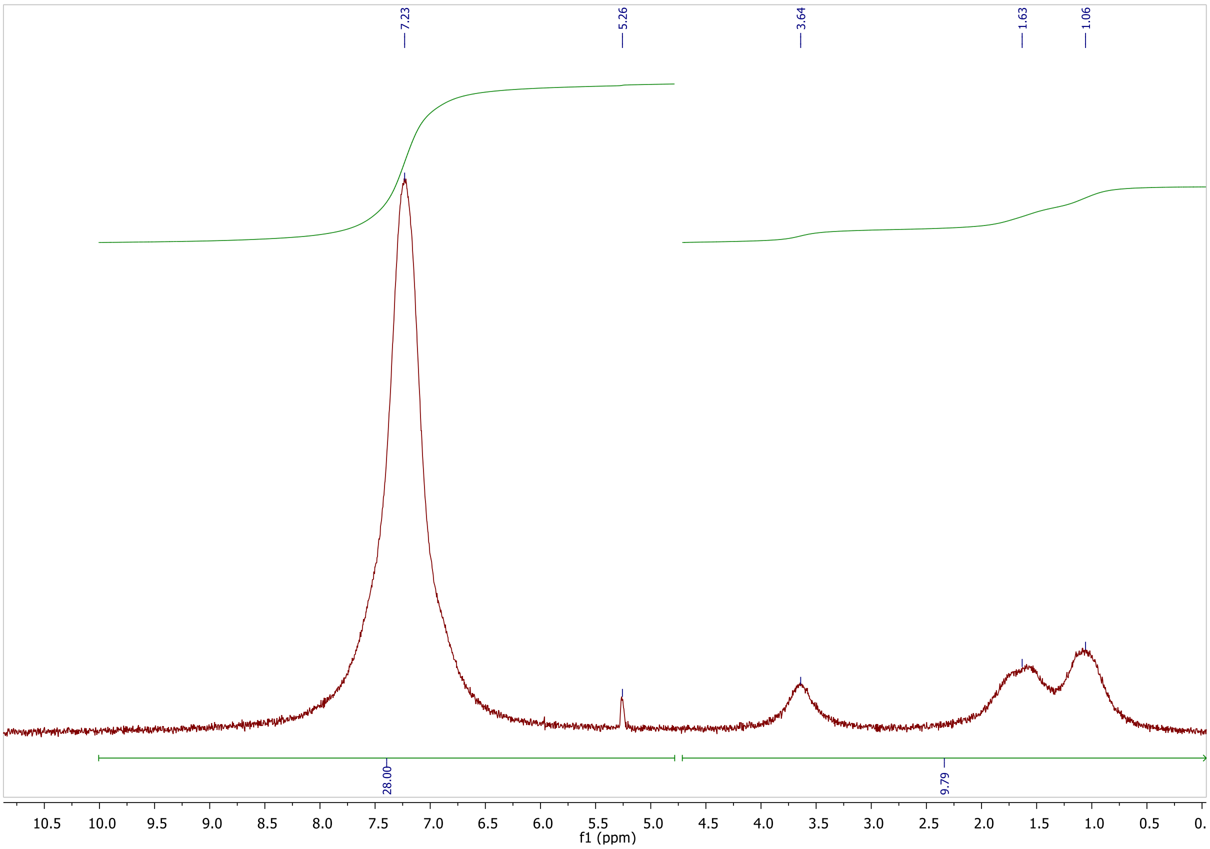

Figure S38. ^{2}H **NMR (46 MHz, unlocked) spectrum of** D_{38}**-*S*,*S*-DACH-phenyl Trost Ligand, [**D_{38}**]-(*S*,*S*)-2**

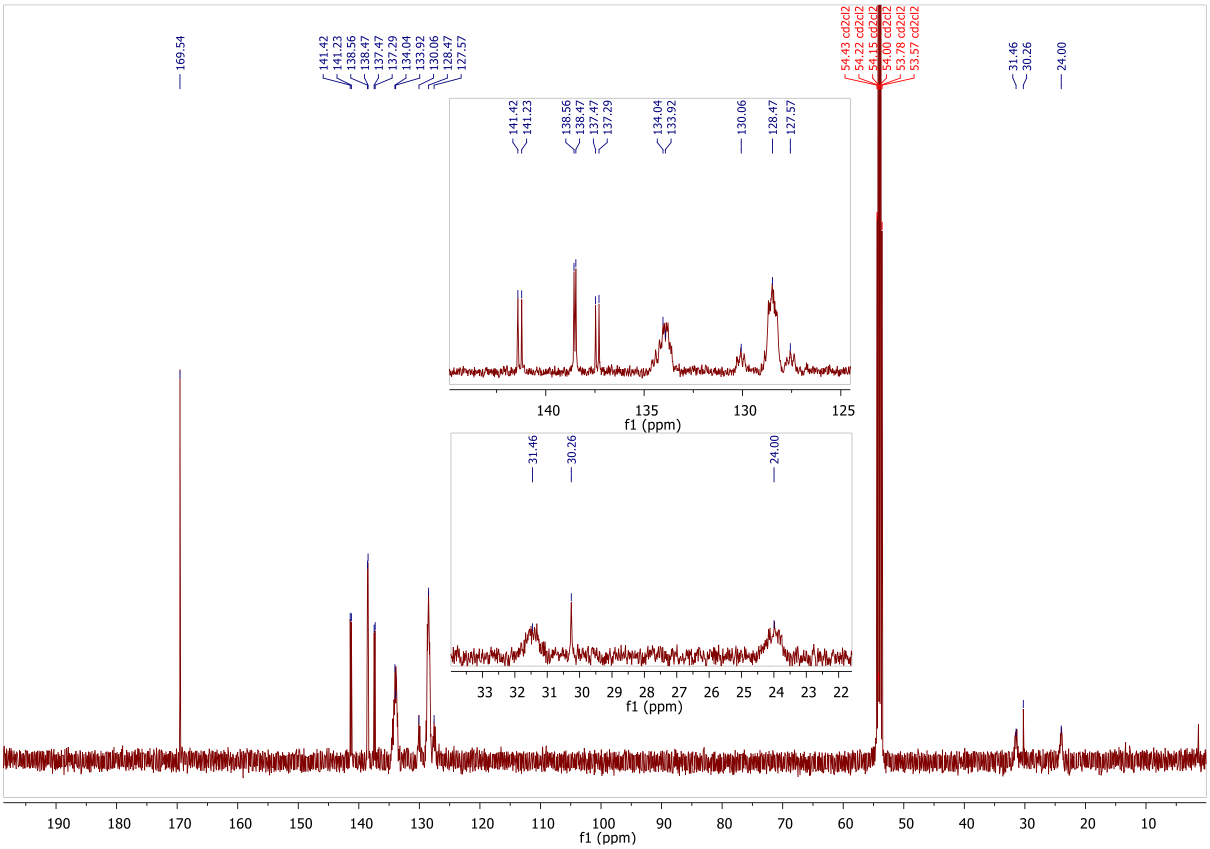

Figure S39. ^{13}**C NMR (126 MHz, CD_2Cl_2) spectrum of D_{38}-*S*,*S*-DACH-phenyl Trost Ligand, [D_{38}]-(*S*,*S*)-2**

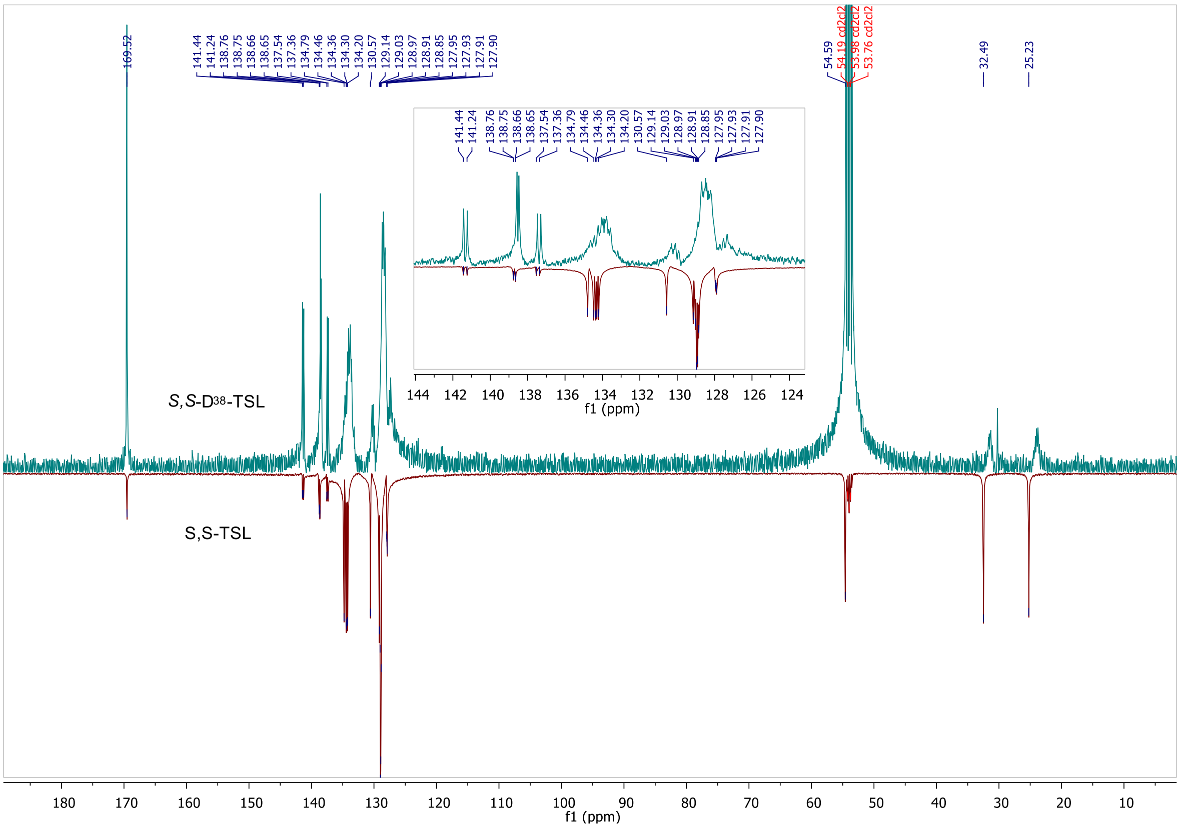

Figure S40. ^{13}**C NMR (126 MH z, CD_2Cl_2) spectrum of D_{38}-*S*,*S*-DACH-phenyl Trost Ligand, [D_{38}]-(*S*,*S*)-2 (green) and (*S*,*S*)-2 (red)**

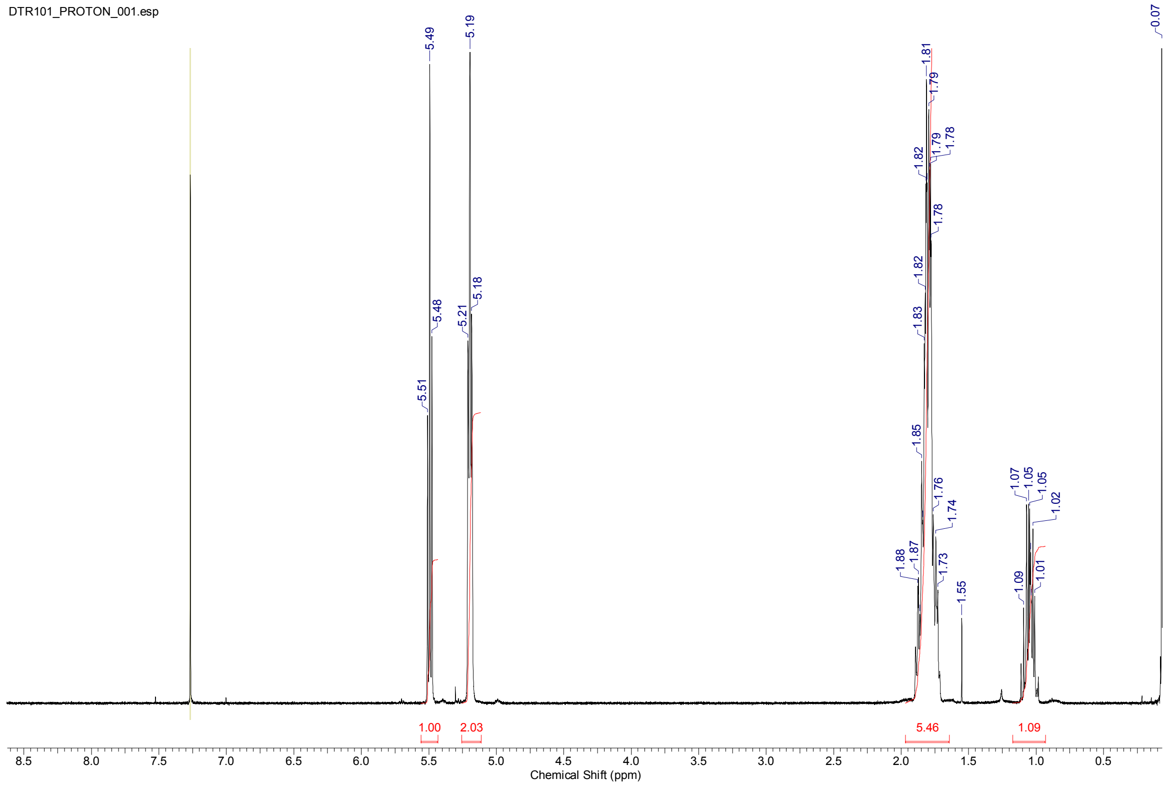

Figure S41. ^{1}**H NMR (400 MHz, CDCl$_3$) spectrum of bis(η^3-cyclohexenylpalladium(II)) dichloride, 16**

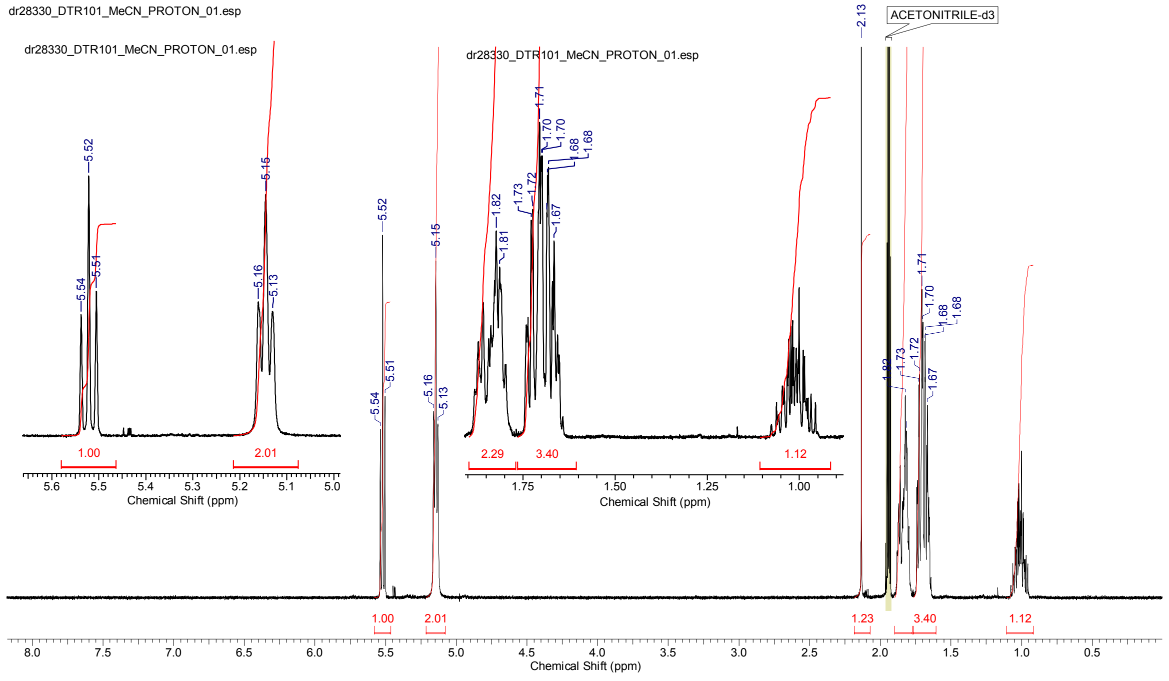

Figure S42. ^{1}**H NMR (400 MHz, CD$_3$CN) spectrum of bis(η^3-cyclohexenylpalladium(II)) dichloride, 16**

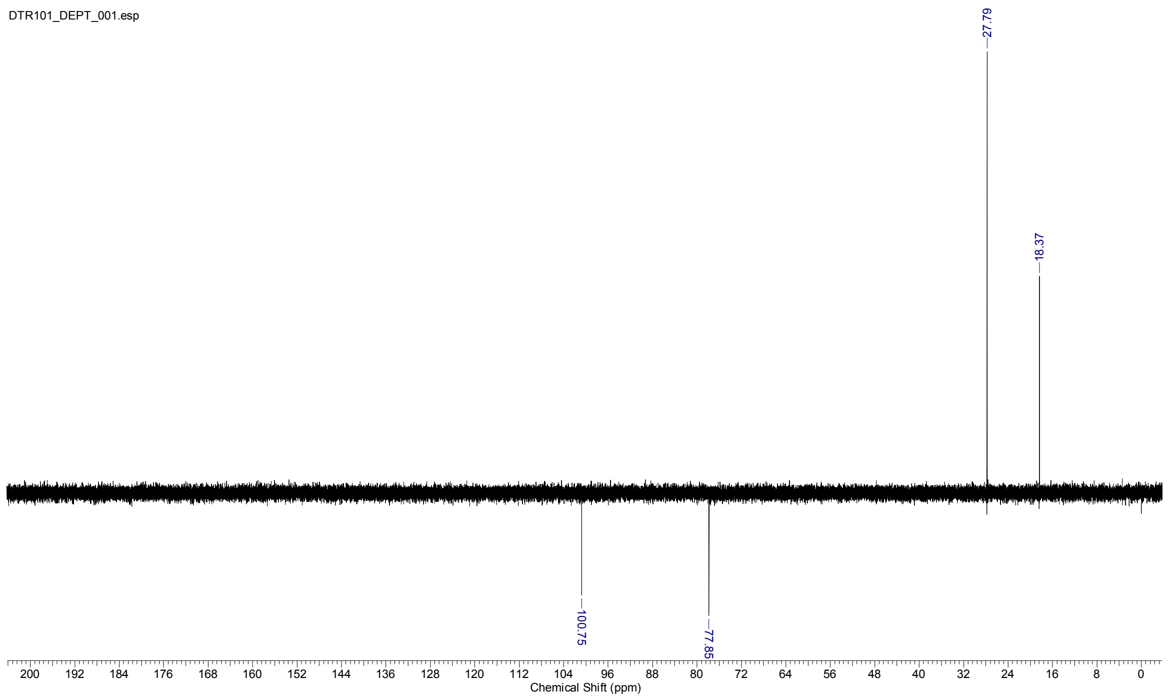

Figure S43. ^{13}C **NMR (100 MHz,** $CDCl_3$**) spectrum of bis(*η*^{3}-cyclohexenylpalladium(II)) dichloride, 16**

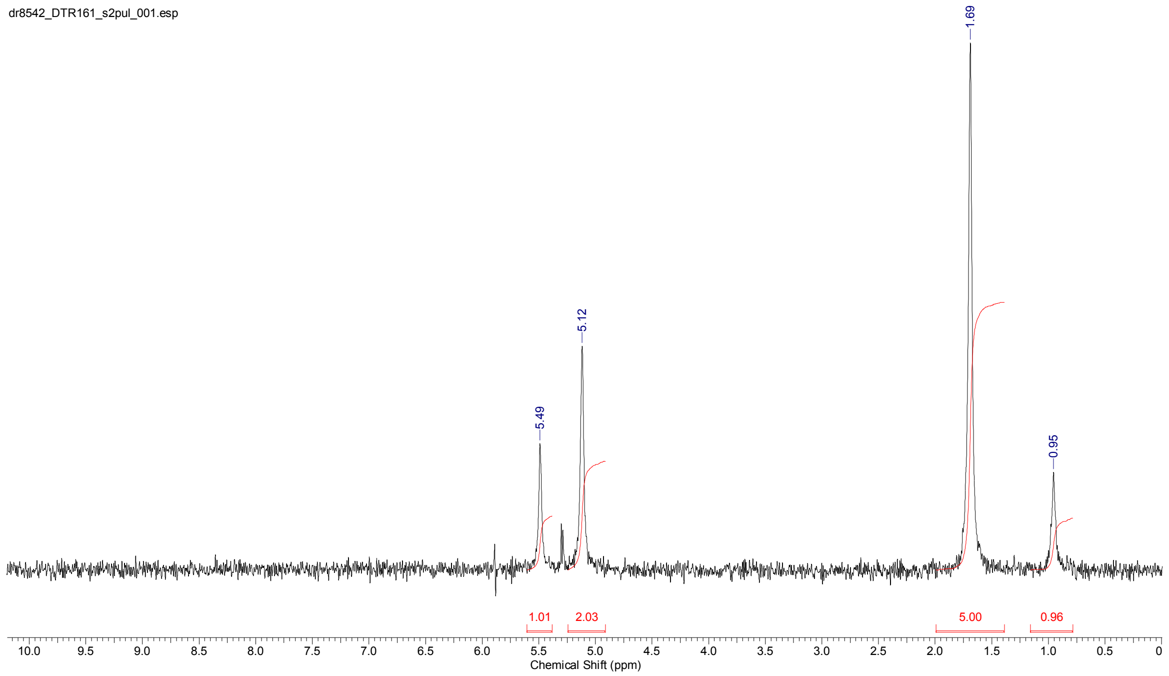

Figure S44. ^{2}H **NMR (77 MHz, unlocked,** CH_2Cl_2**) spectrum of bis(*η*^{3}-D_9-cyclohexenylpalladium(II)) dichloride, [D_{18}]-16**

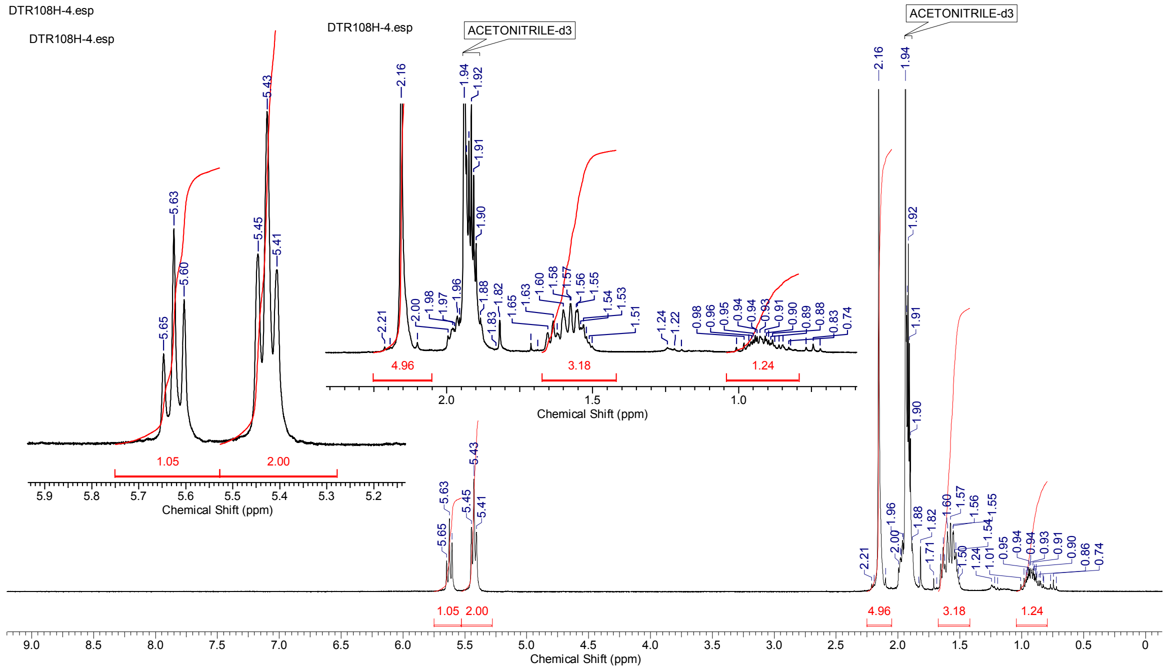

Figure S45. ^{1}**H NMR (300 MHz,** CD_3CN**) spectrum of [bis-acetonitrile(**η^3**-cyclohexenyl)palladium(II)] tetrakis(pentafluorophenyl)borate, [17][**BAr_F**]**

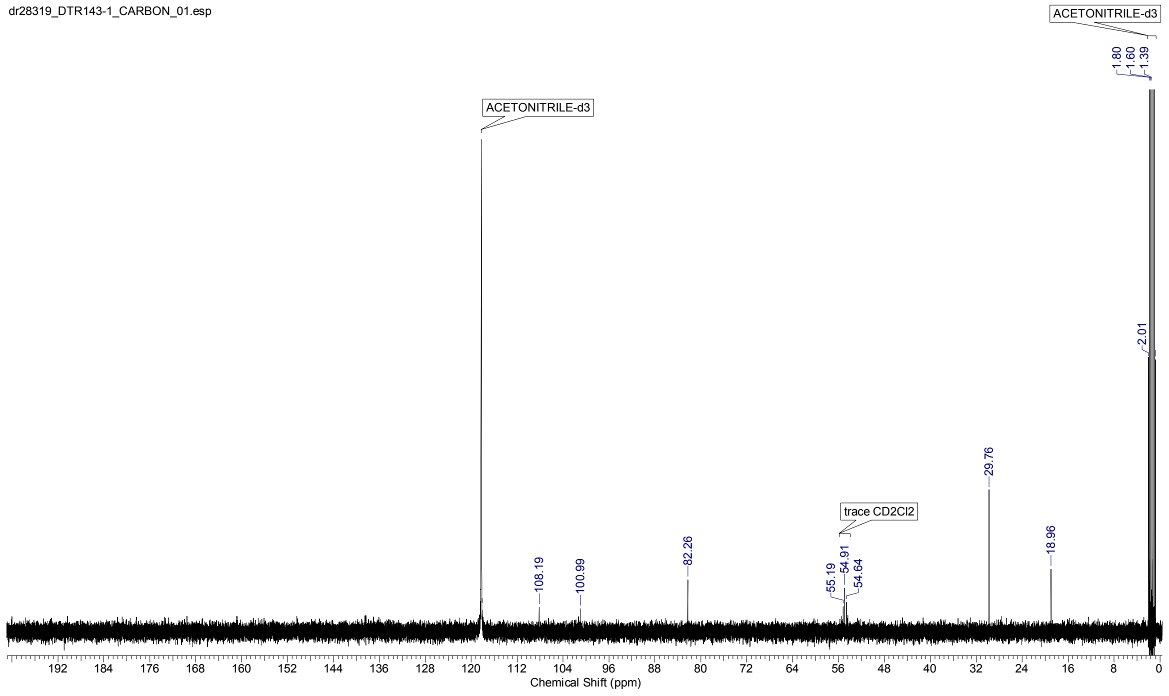

Figure S46. ^{13}**C NMR (100 MHz,** CD_3CN**) spectrum of [bis-acetonitrile(**η^3**-cyclohexenyl)palladium(II)] tetrakis(pentafluorophenyl)borate, [17][**BAr_F**]**

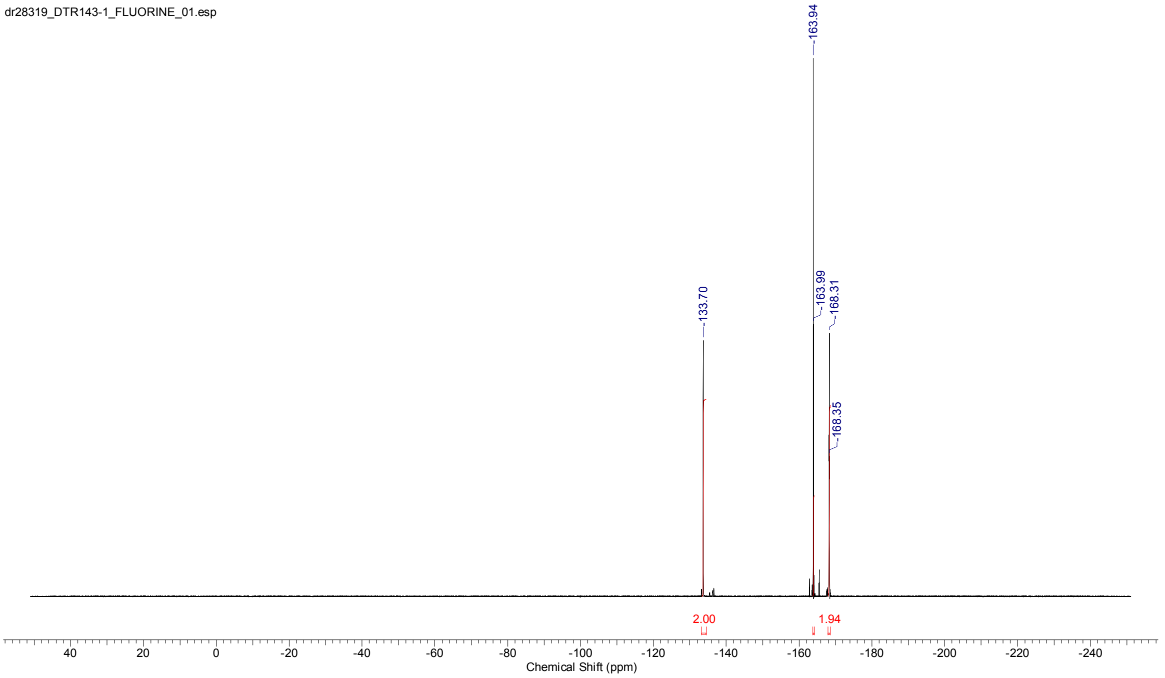

Figure S47. ^{19}**F NMR (376 MHz, CD_3CN) spectrum of [bis-acetonitrile(η^3-cyclohexenyl)palladium(II)] tetrakis(pentafluorophenyl)borate, [17][BAr$_F$]**

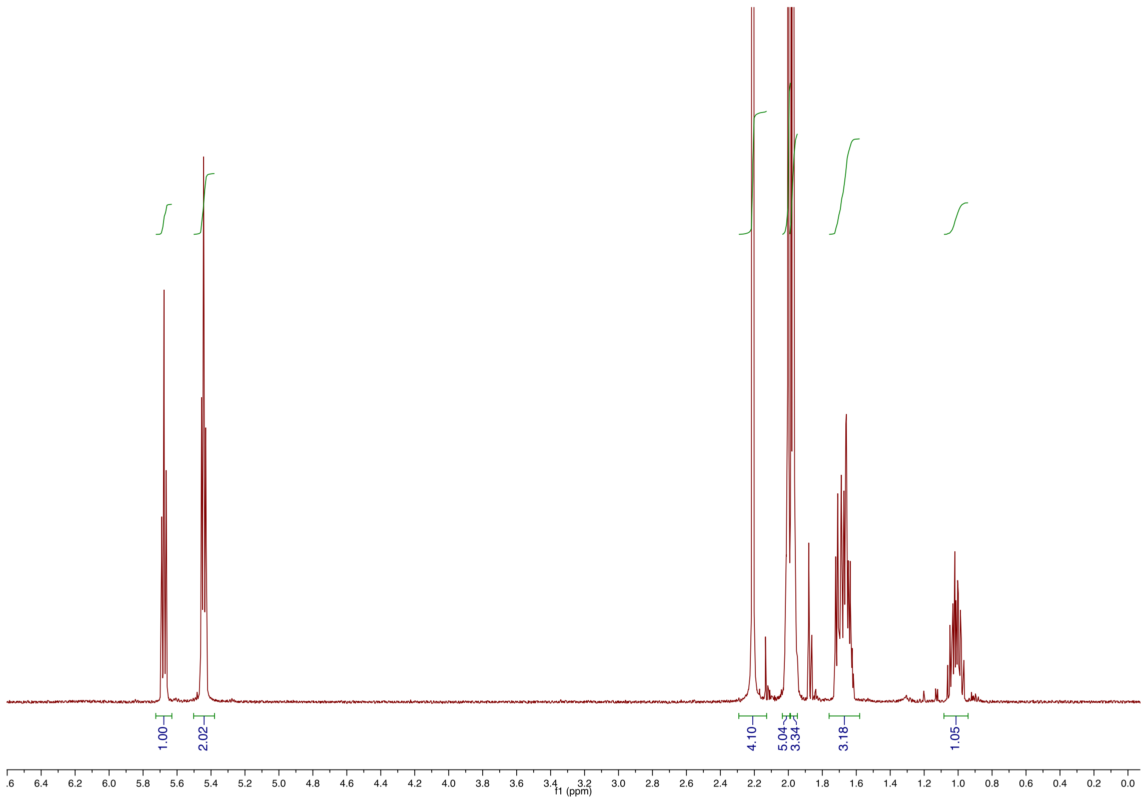

Figure S48. ^{1}**H NMR (500 MHz, CD_3CN) spectrum of [bis-acetonitrile(η^3-cyclohexenyl)palladium(II)] triflate, [17][OTf]**

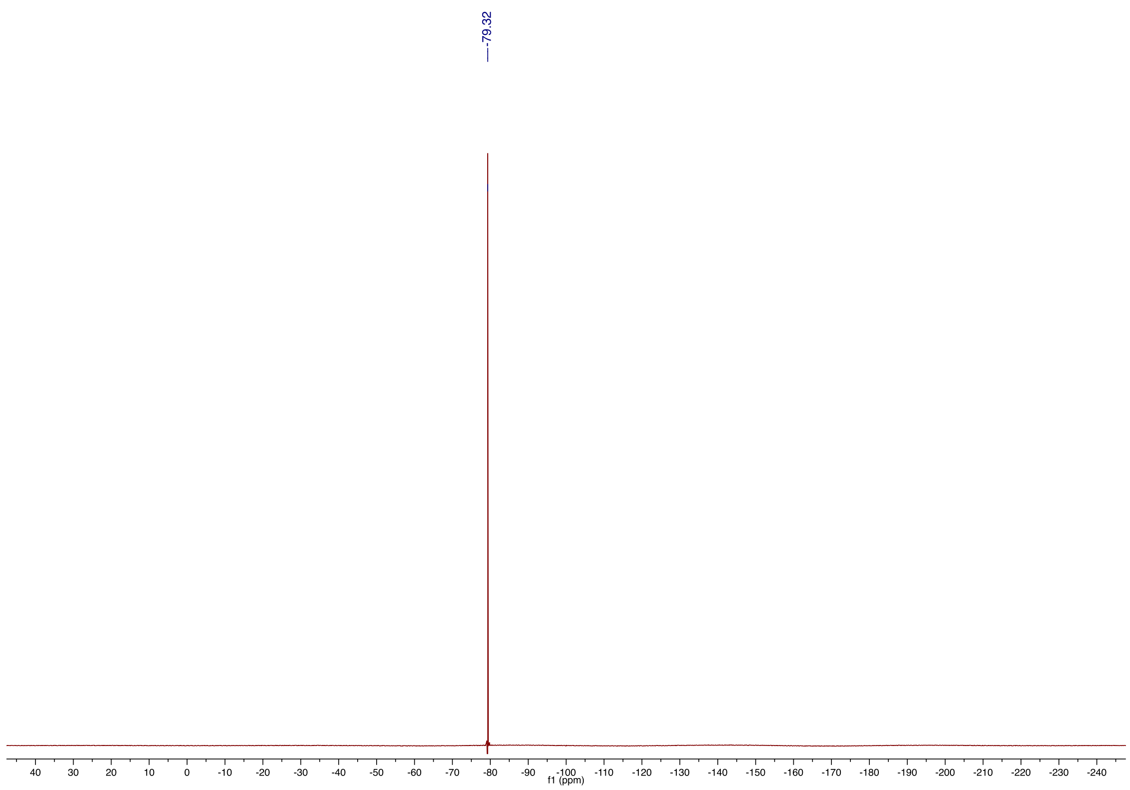

Figure S49. ^{19}F **NMR (470 MHz,** CD_3CN**) spectrum of [bis-acetonitrile(**η^3**-cyclohexenyl)palladium(II)] triflate, [17][OTf]**

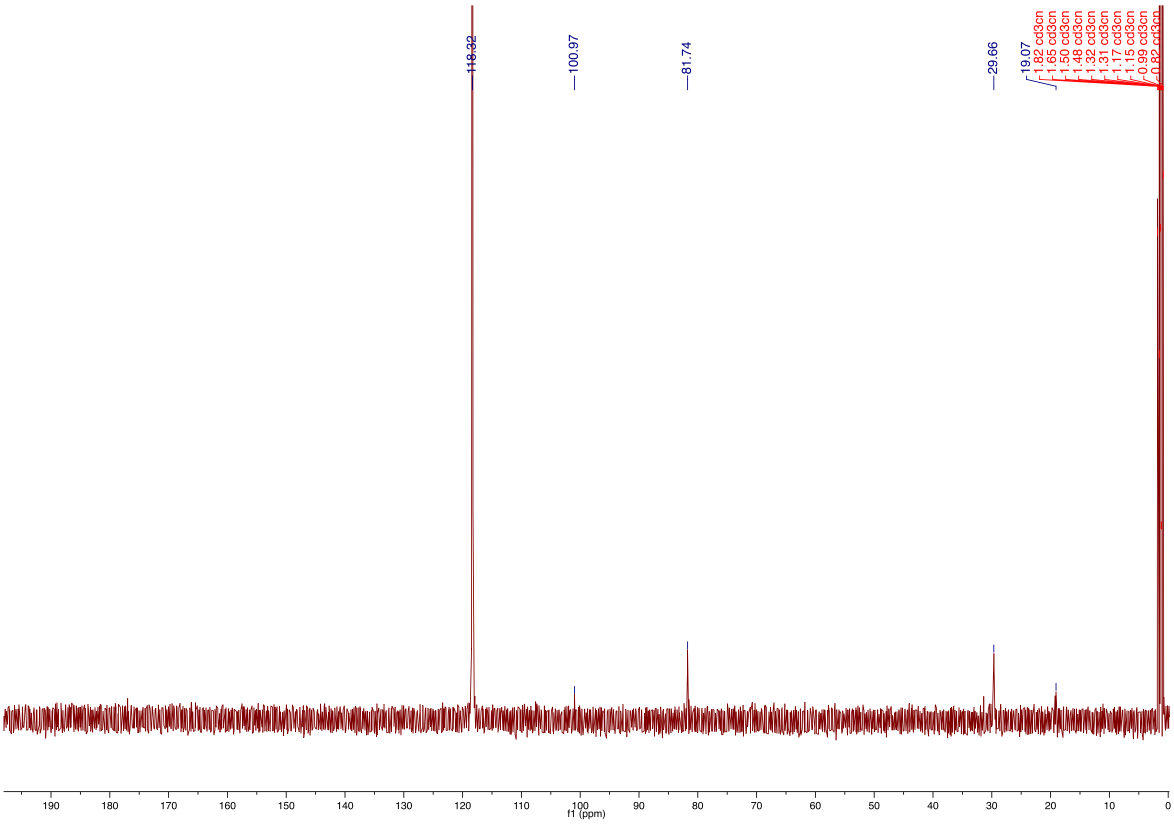

Figure S50. ^{13}C **NMR (126 MHz,** CD_3CN**) spectrum of [bis-acetonitrile(**η^3**-cyclohexenyl)palladium(II)] triflate, [17][OTf]**

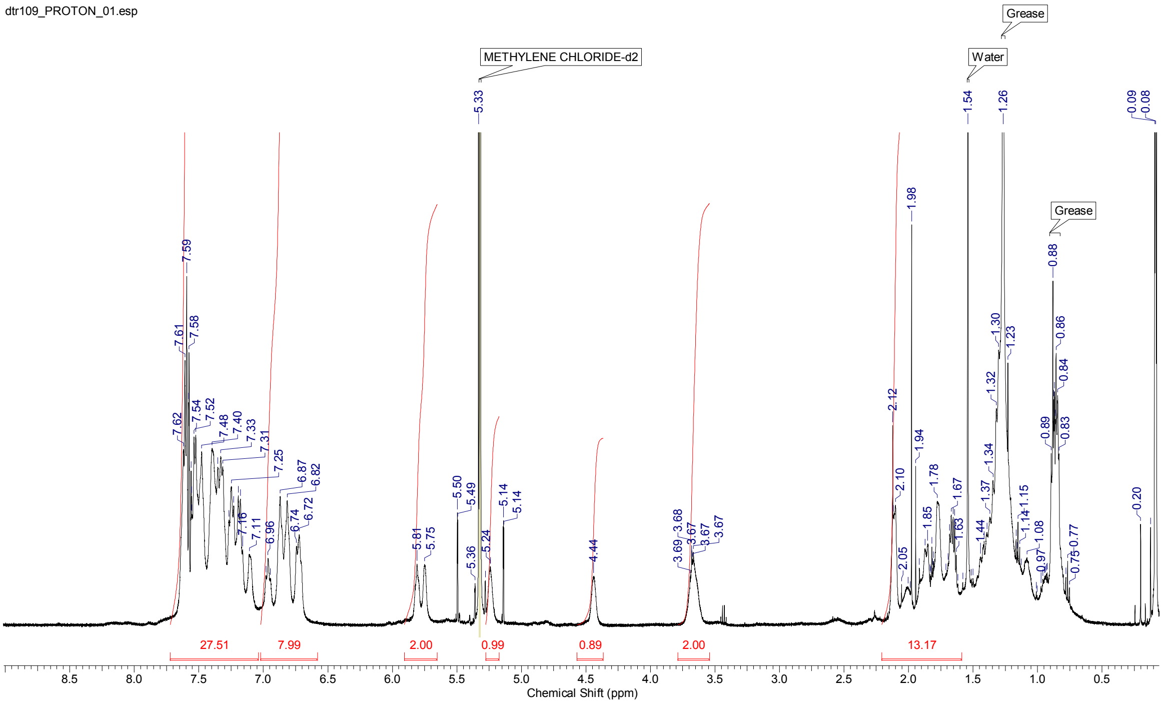

Figure S51. ^{1}**H NMR (500 MHz, CH_2Cl_2, 4.05 mM) spectrum of [(η^3-cyclohexenyl)palladium(II){(1*R*,2*R*)-1,2-*N*-*N*'-bis[2'-(diphenylphosphino)benzoyl]diaminocyclohexane}] tetrakis(pentafluorophenyl)borate, [(*R*,*R*)-1][BAr$_F$]**

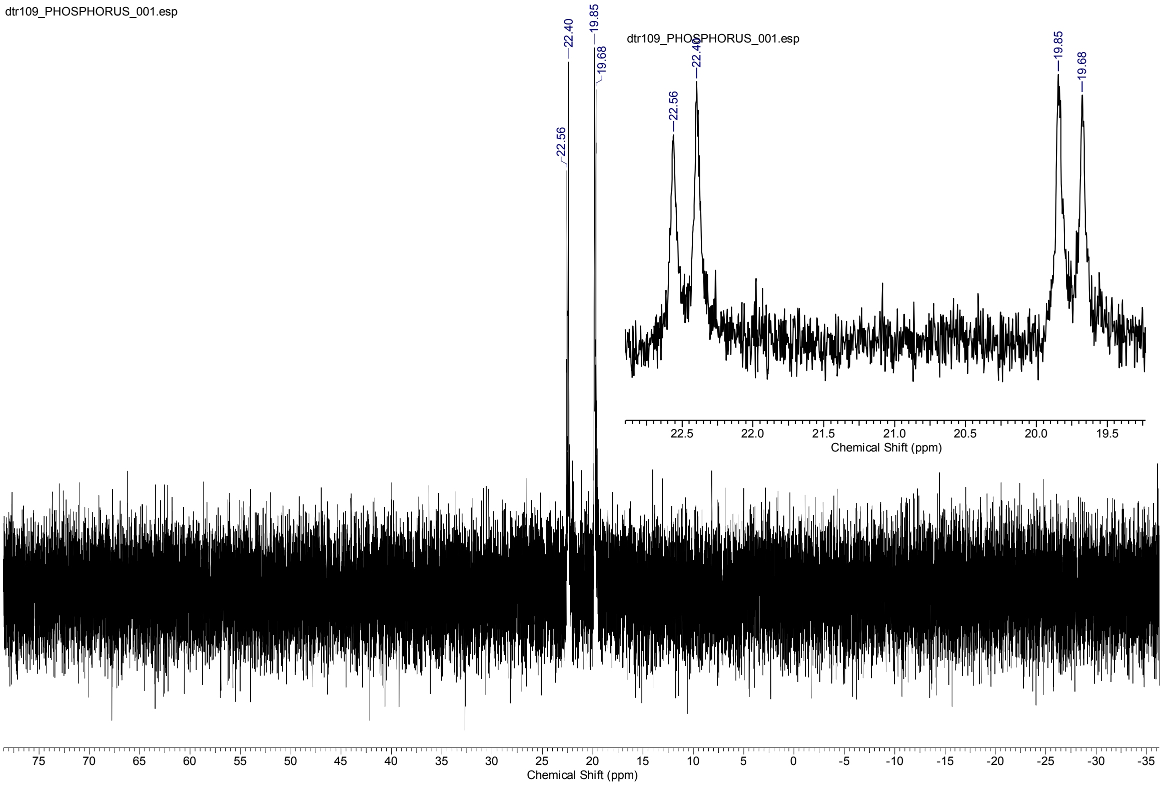

Figure S52. ^{31}**P NMR (202 MHz, CH_2Cl_2, 4.05 mM) spectrum of [(η^3-cyclohexenyl)palladium(II){(1*R*,2*R*)-1,2-*N*-*N*'-bis[2'-(diphenylphosphino)benzoyl]diaminocyclohexane}] tetrakis(pentafluorophenyl)borate, [(*R*,*R*)-1][BAr$_F$]**

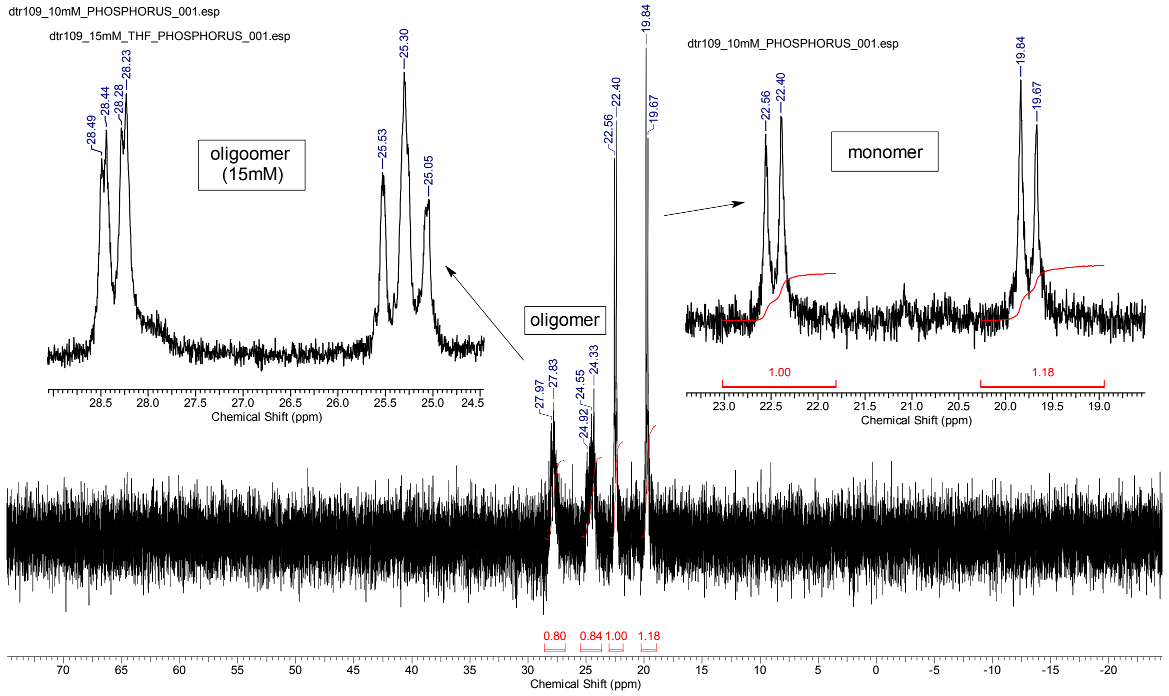

Figure S53. **^{31}P NMR (202 MHz, CH_2Cl_2, 10 mM) spectrum of [(η^3-cyclohexenyl)palladium(II){(1*R*,2*R*)-1,2-*N*-*N*'-bis[2'-(diphenylphosphino)benzoyl]diaminocyclohexane}] tetrakis(pentafluorophenyl)borate, [(*R*,*R*)-1][BAr$_F$]**

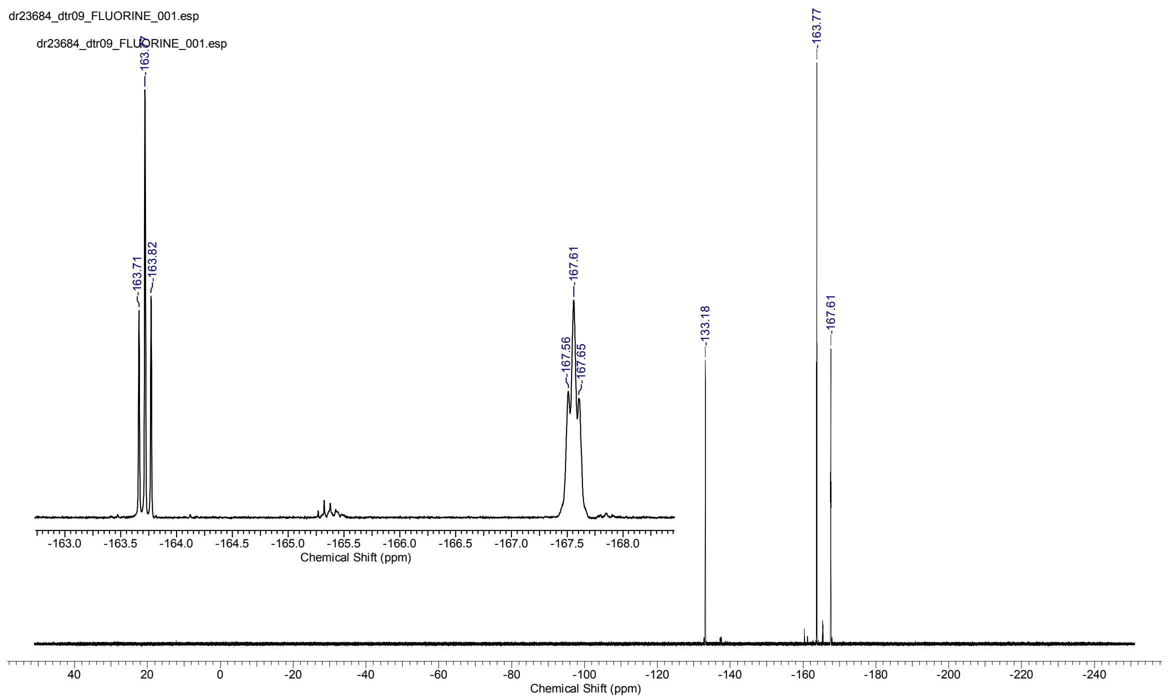

Figure S54. **^{19}F NMR (376 MHz, CH_2Cl_2) spectrum of [(η^3-cyclohexenyl)palladium(II){(1*R*,2*R*)-1,2-*N*-*N*'-bis[2'-(diphenylphosphino)benzoyl]diaminocyclohexane}] tetrakis(pentafluorophenyl)borate, [(*R*,*R*)-1][BAr$_F$]**

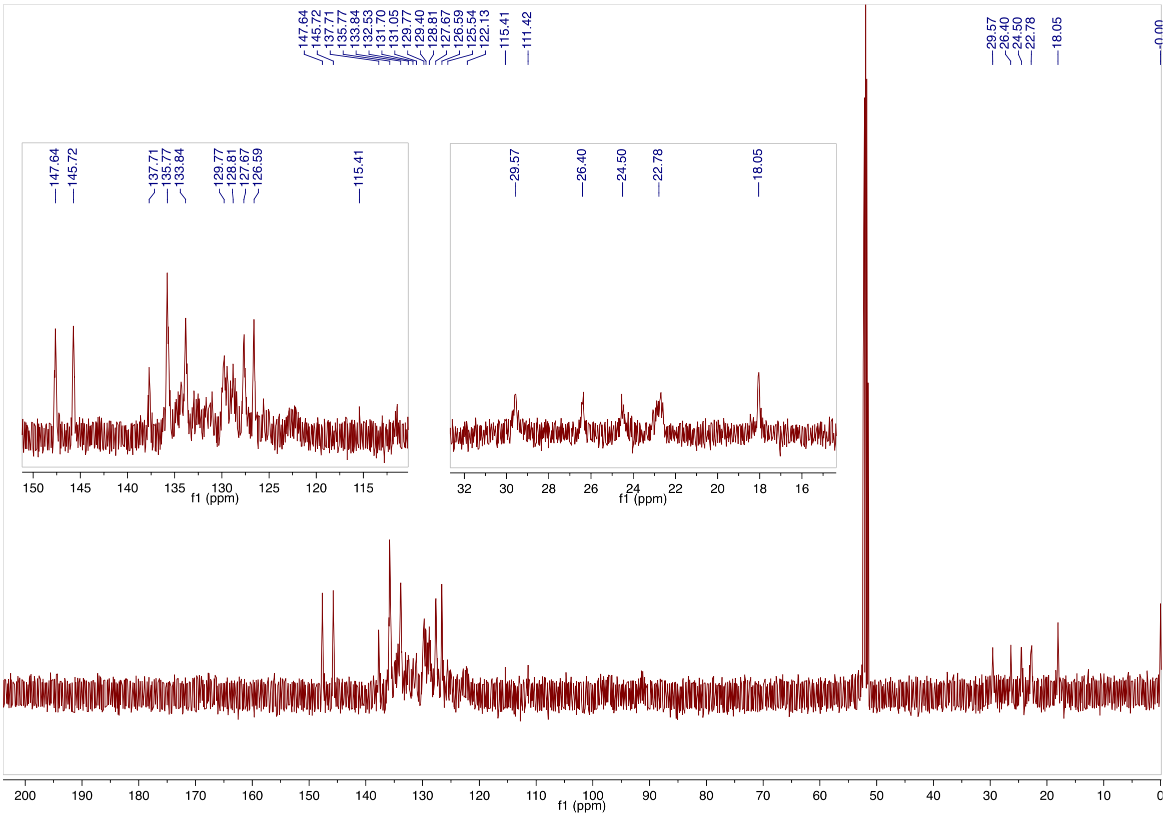

Figure S55. **^{13}C NMR (126 MHz, CH_2Cl_2) spectrum of [(η^3-cyclohexenyl)palladium(II){(1*R*,2*R*)-1,2-*N*-*N*'-bis[2'-(diphenylphosphino)benzoyl]diaminocyclohexane}] tetrakis(pentafluorophenyl)borate, [(*R*,*R*)-1][BAr_F]**

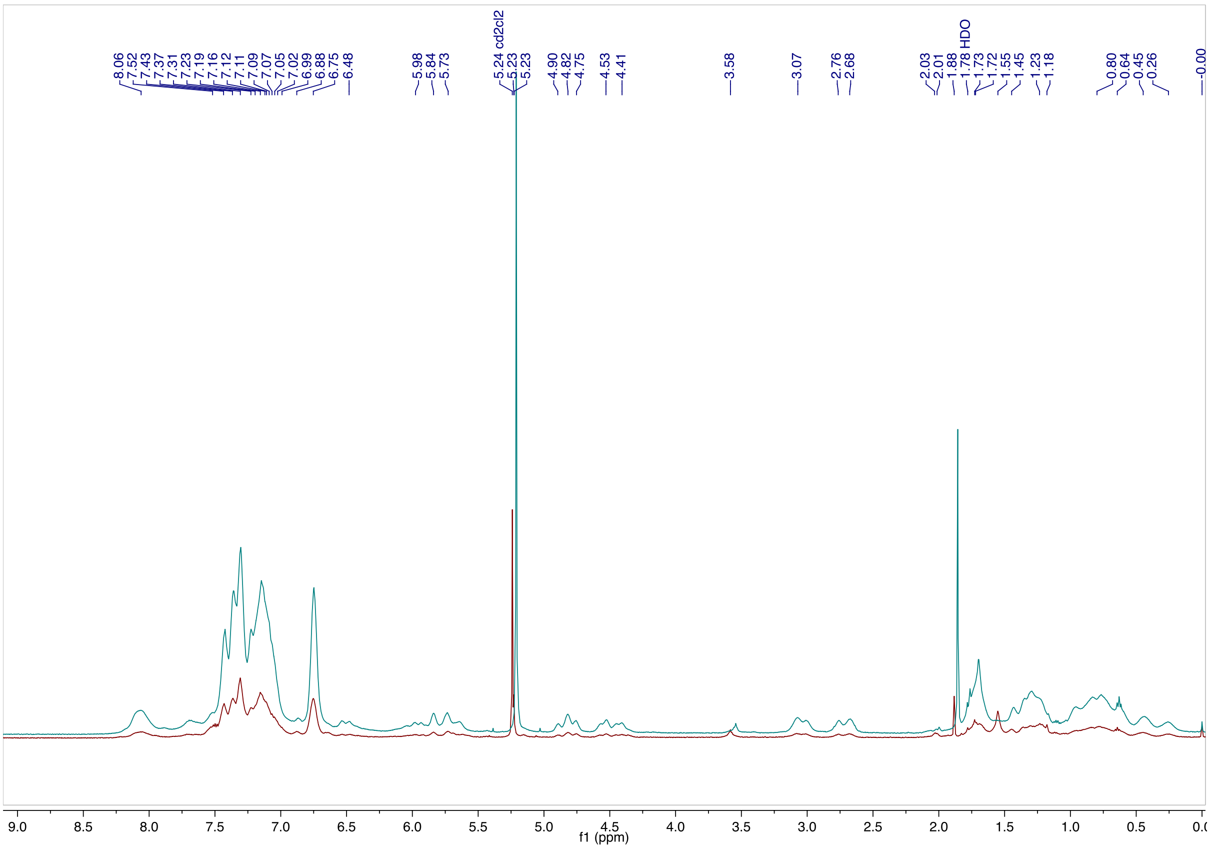

Figure S56. **^{1}H NMR (500 MHz, CH_2Cl_2) spectra of [(*R*,*R*)-1][BAr_F] and [(*S*,*S*)-1][BAr_F]**

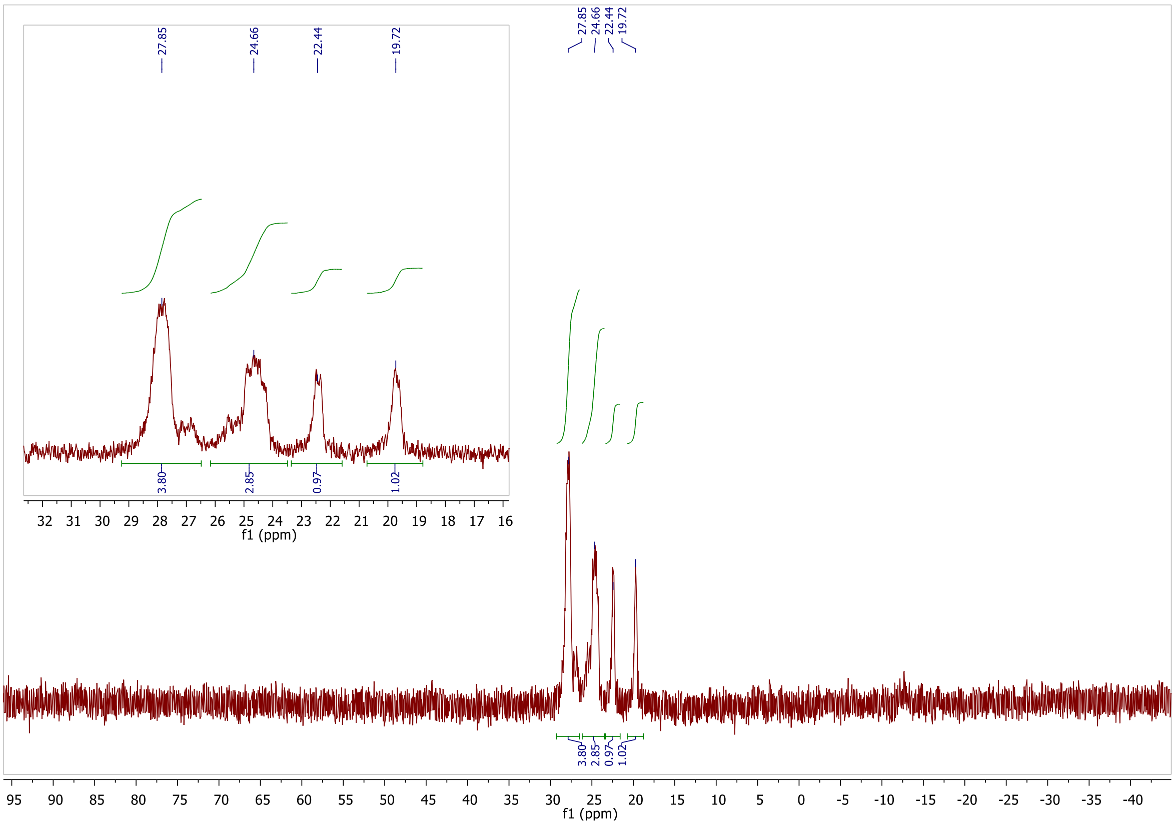

Figure S57. ^{31}**P NMR (202 MHz, unlocked) spectrum of [[D$_{47}$]-(*S*,S)-1][BAr$_F$]**

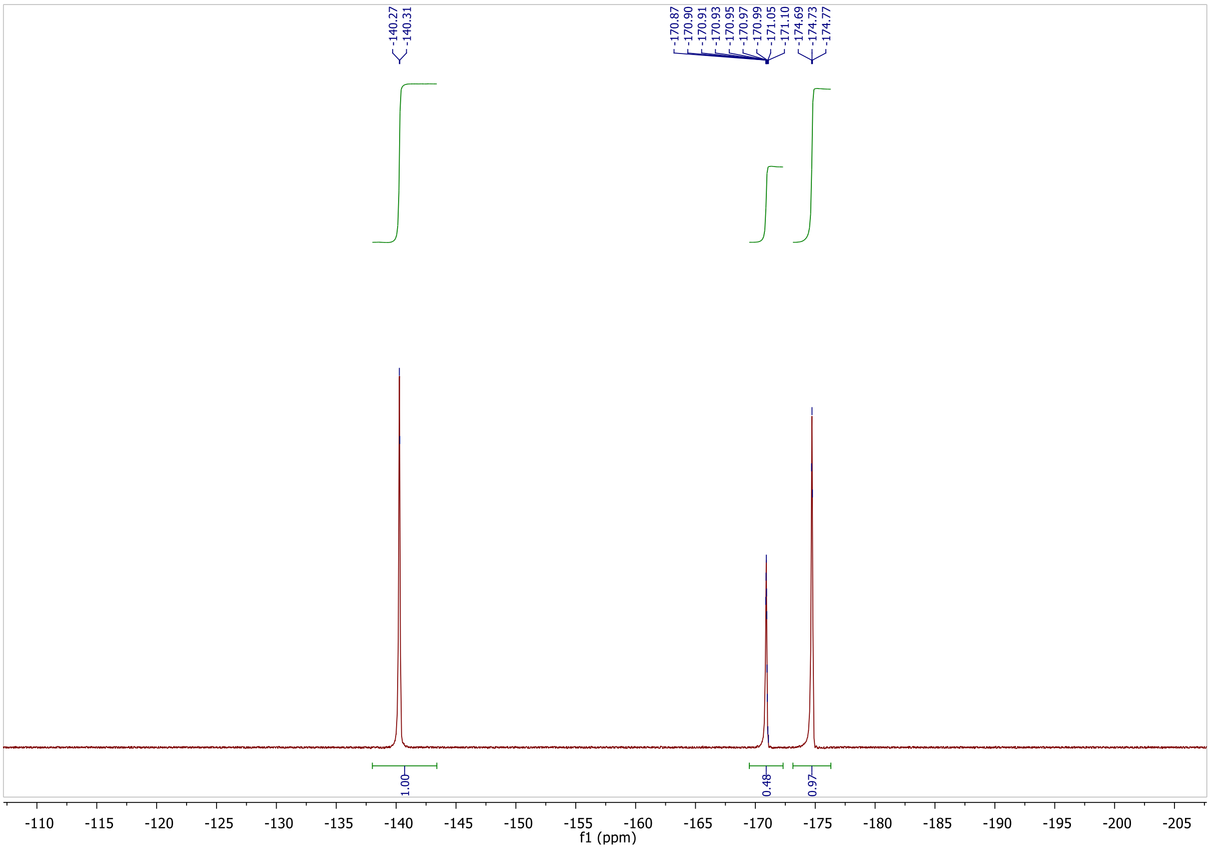

Figure S58. ^{19}**F NMR (470 MHz, unlocked) spectrum of [[D$_{47}$]-(*S*,S)-1][BAr$_F$]**

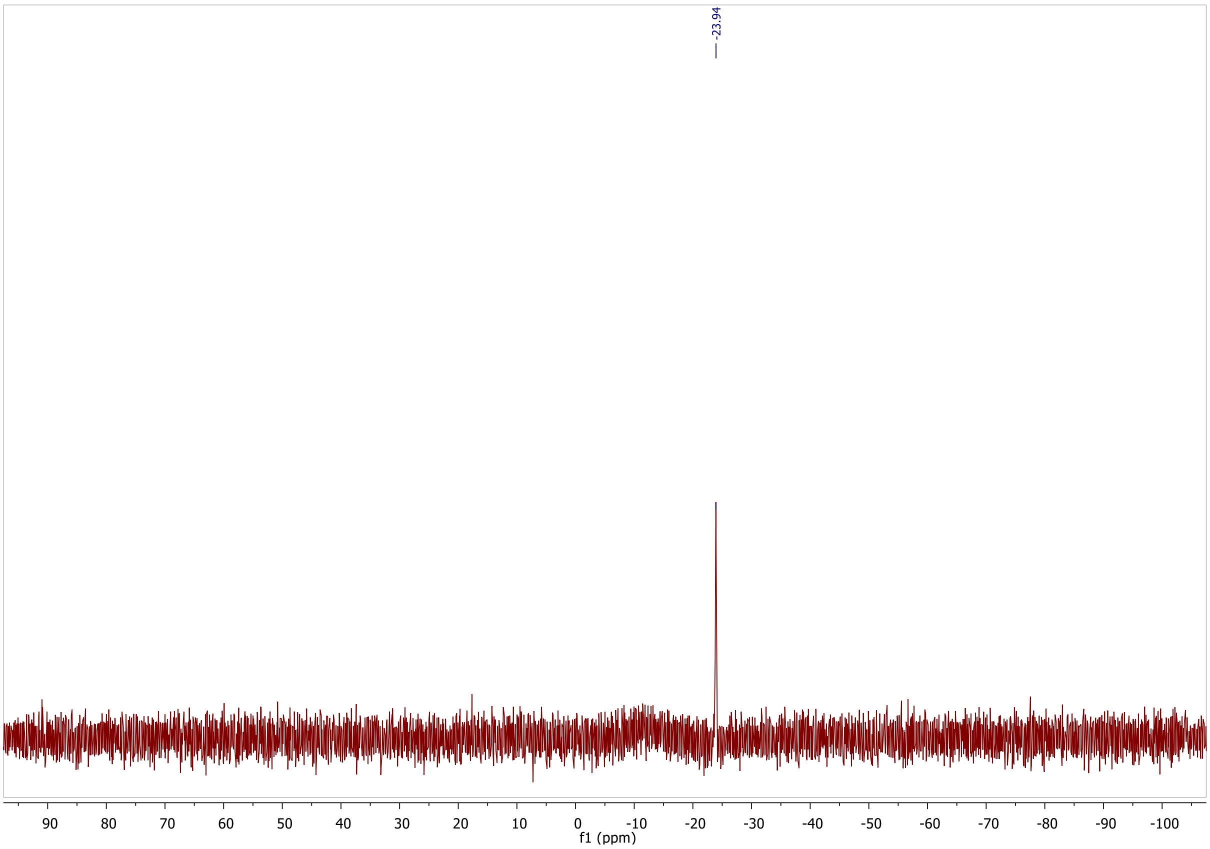

Figure S59. ^{11}**B NMR (160 MHz, unlocked) spectrum of [D$_{47}$]-(*S*,S)-1**

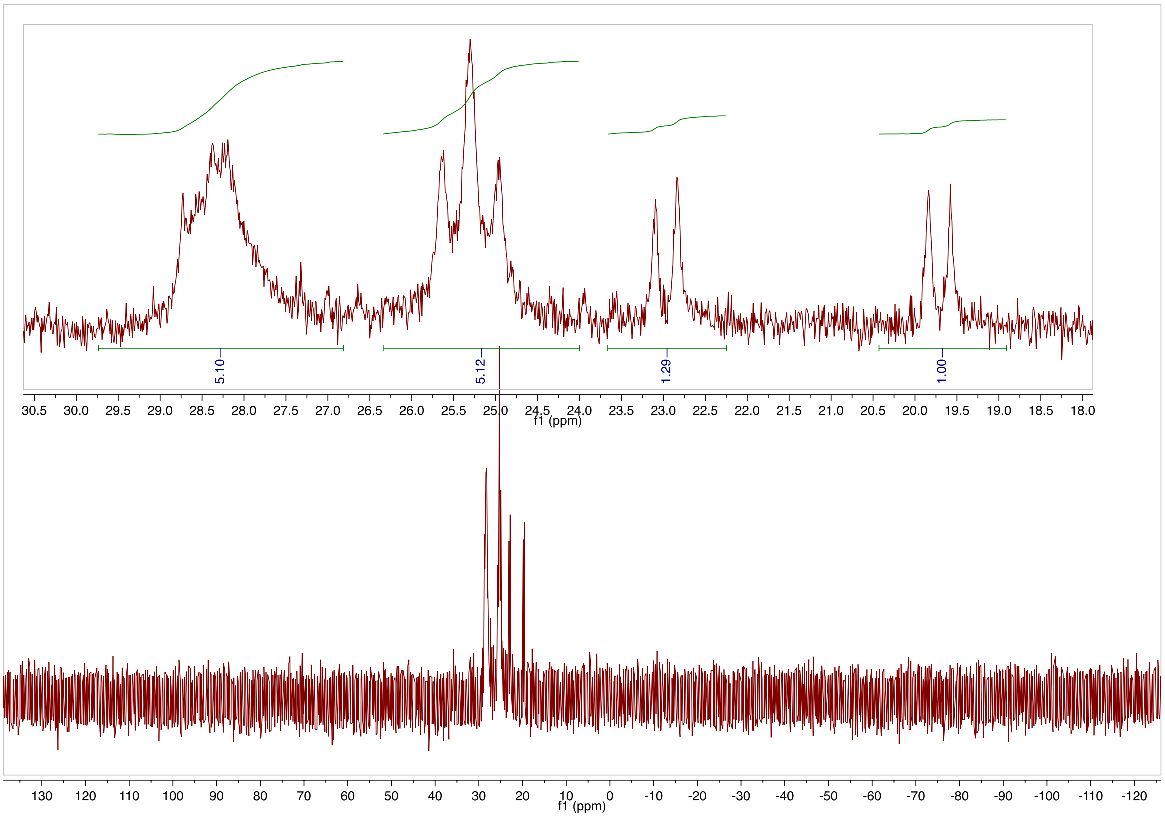

Figure S60. ^{31}**P NMR (121 MHz, unlocked) spectrum of [(η^3-cyclohexenyl)palladium(II){(1*S*,2*S*)-1,2-*N*-*N*'-bis[2'-(diphenylphosphino)benzoyl] diaminocyclohexane}] triflate, [(*S*,*S*)-1][OTf]**

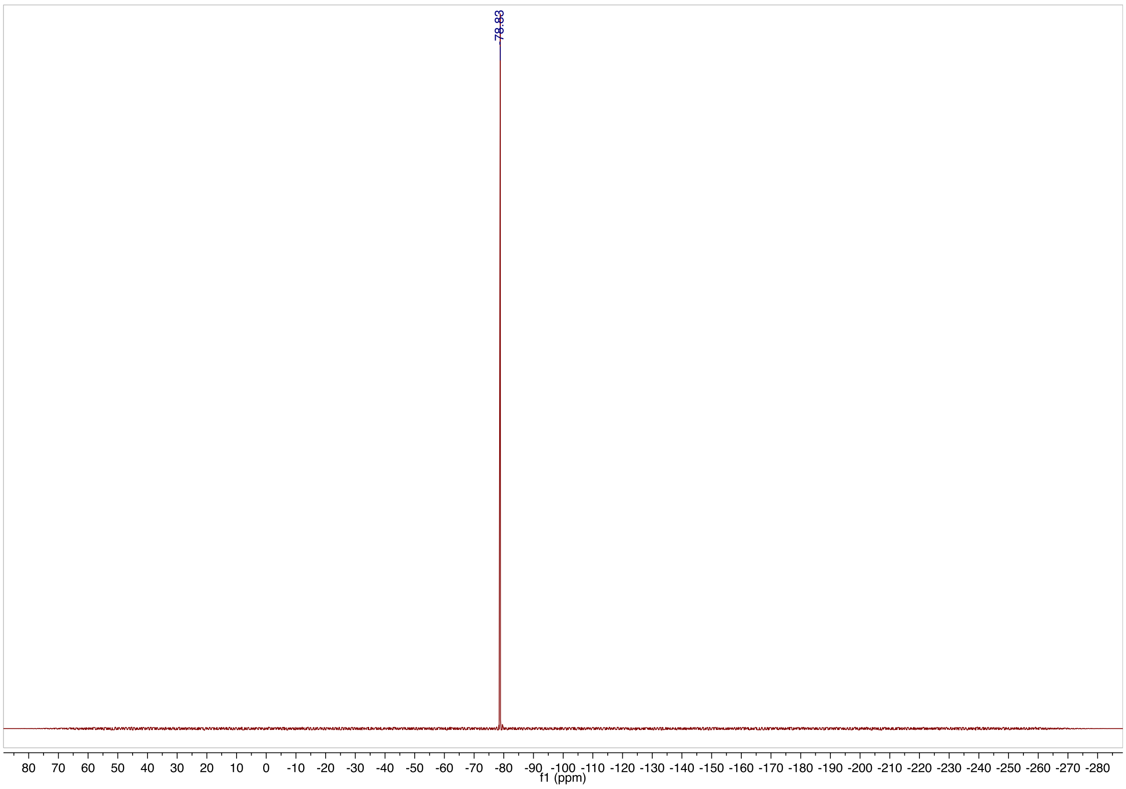

Figure S61. ^{19}F **NMR (228 MHz, unlocked) spectrum of [(η^3-cyclohexenyl)palladium(II){(1*S*,2*S*)-1,2-*N*-*N*'-bis[2'-(diphenylphosphino)benzoyl] diaminocyclohexane}] triflate, [(*S*,*S*)-1][OTf]**

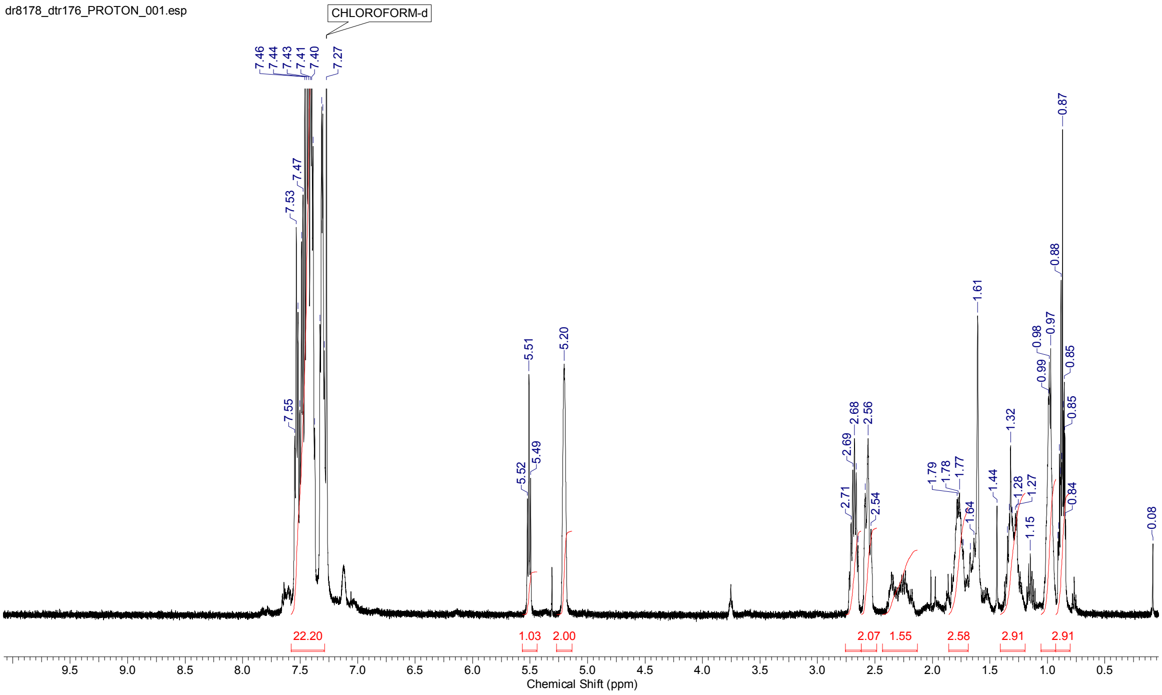

Figure S62. ^{1}H **NMR (500 MHz, $CDCl_3$) spectrum of [(η^3-cyclohexenyl)palladium(II){1,3-(diphenylphosphino)propane}] tetrakis(pentafluorophenyl)borate, [18][BAr_F]**

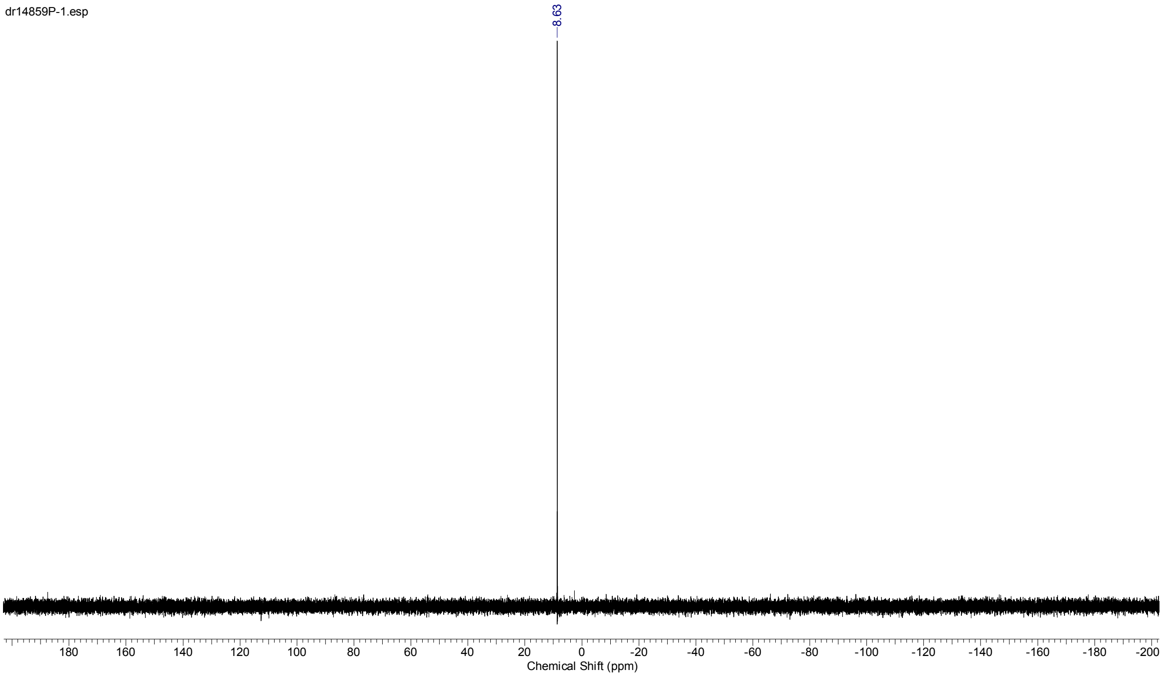

Figure S63. ^{31}P NMR (122 MHz, $CDCl_3$) spectrum of [(η^3-cyclohexenyl)palladium(II){1,3-(diphenylphosphino)propane}] tetrakis(pentafluorophenyl)borate, [18][BAr$_F$]

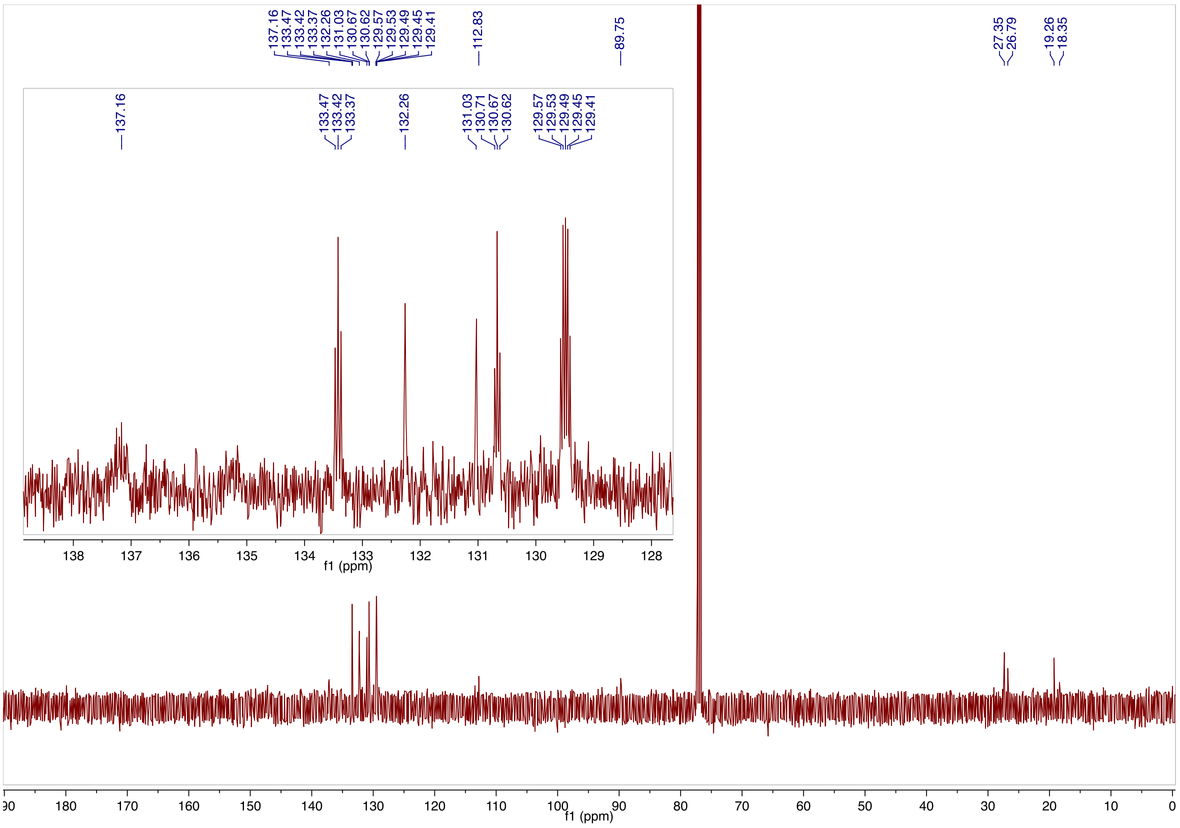

Figure S64. ^{13}C NMR (126 MHz, $CDCl_3$) spectrum of [(η^3-cyclohexenyl)palladium(II){1,3-(diphenylphosphino)propane}] tetrakis(pentafluorophenyl)borate, [18][BAr$_F$]

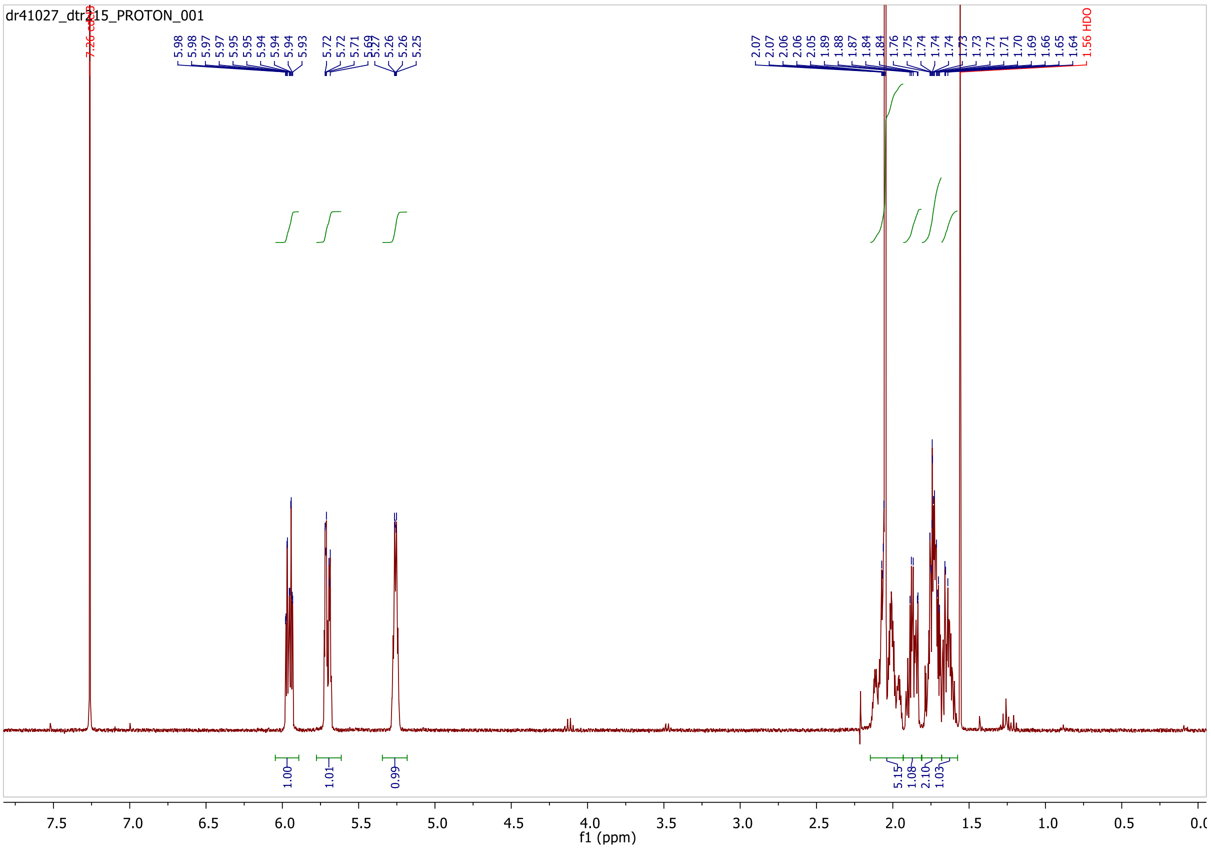

Figure S65. ^{1}**H NMR (400 MHz, CDCl**$_3$**) spectrum of cyclohex-2-en-1-yl acetate 19**

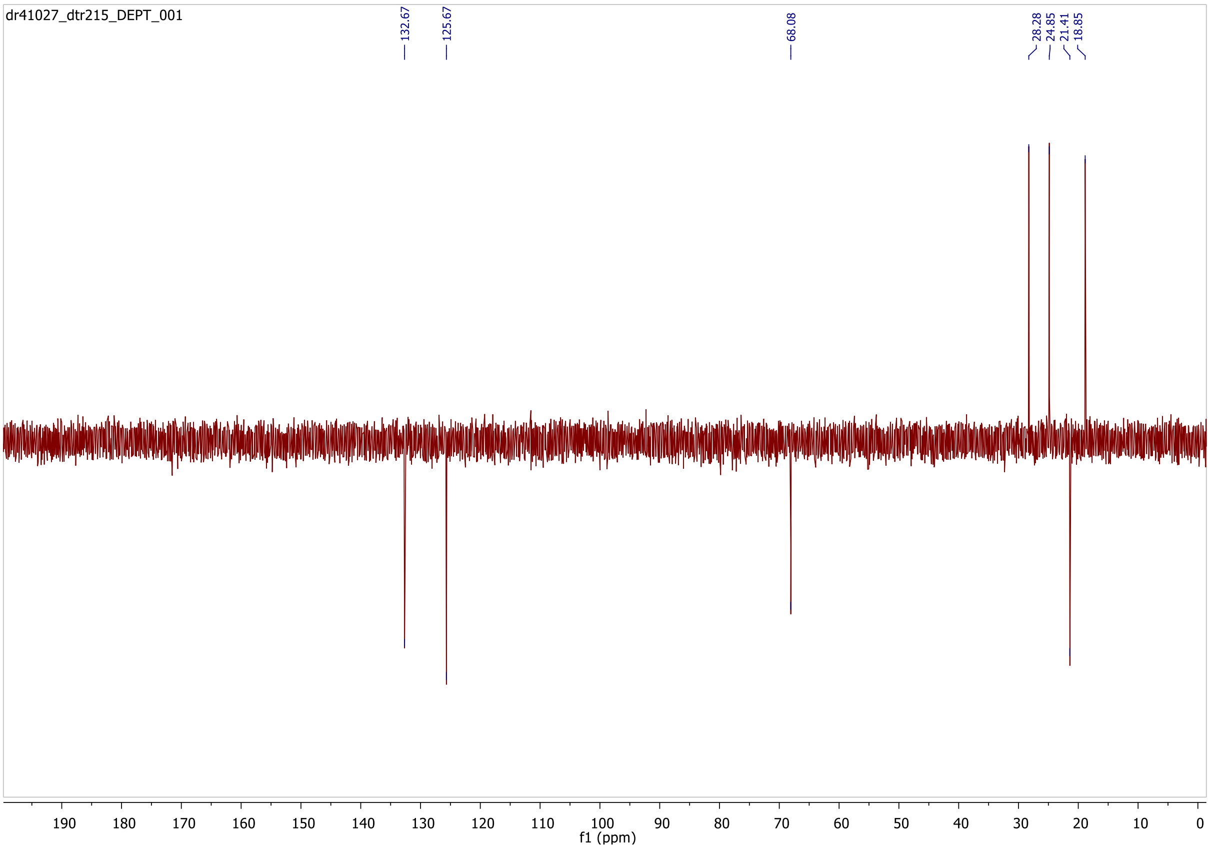

Figure S66. DEPT NMR (101 MHz, CDCl$_3$**) spectrum of cyclohex-2-en-1-yl acetate 19**

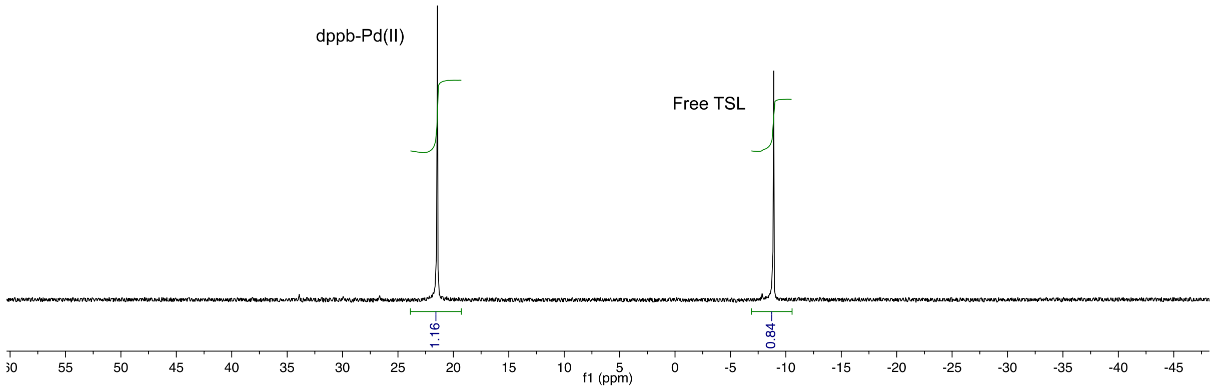

Figure S67. ^{31}P **NMR (202 MHz,** CH_2Cl_2**, unlocked) spectrum illustrating complete ligand exchange between 11 mM [1][BAr$_F$] and 11 mM free dppb ligand. TSL = ligand 2.**

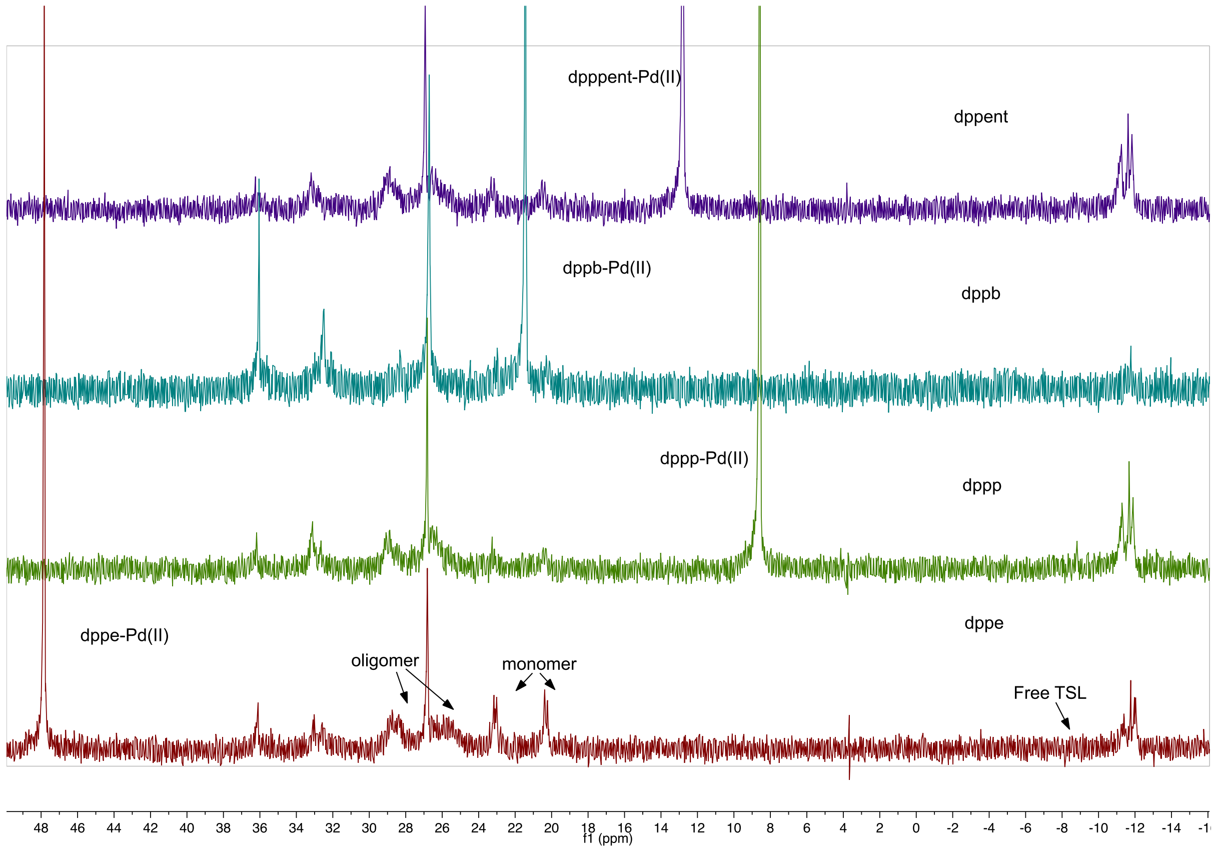

Figure S68. ^{31}P **NMR spectra (202 MHz,** CH_2Cl_2**, unlocked) of [(*R*,R)-1][BAr$_F$] 2:1 mixtures with free bidentate phosphine ligands. TSL = ligand 2.**

S.6.2 SANS data

Table S8. Scattering length densities of complex [1] and selected solvents

Material	Scattering length densities / cm^{-2}
H-THF	1.80×10^{9}
D_8-THF	6.36×10^{10}
CH_2Cl_2	1.73×10^{10}
CD_2Cl_2	3.69×10^{10}
CD_3CN	4.92×10^{10}
**1-BArF**	3.84×10^{10}
**[D_{47}]-1-BArF** (ρ = 2.1 g/cm^3)	7.59×10^{10}
**1-OTf**	2.95×10^{10}

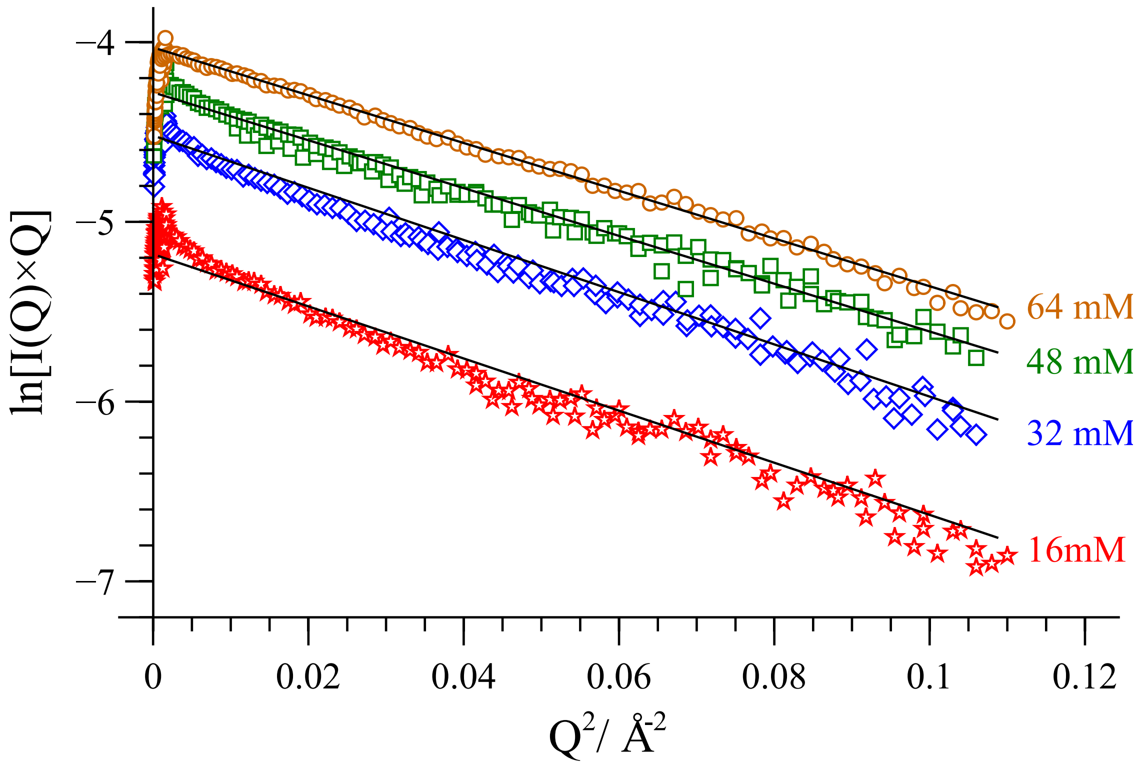

Figure S69. Fitted SANS profiles of 0.25-10.0 wt/vol% [(*R*,*R*)-1][BAr_F] samples. THF-D_8, 25 °C at D22, ILL, France.

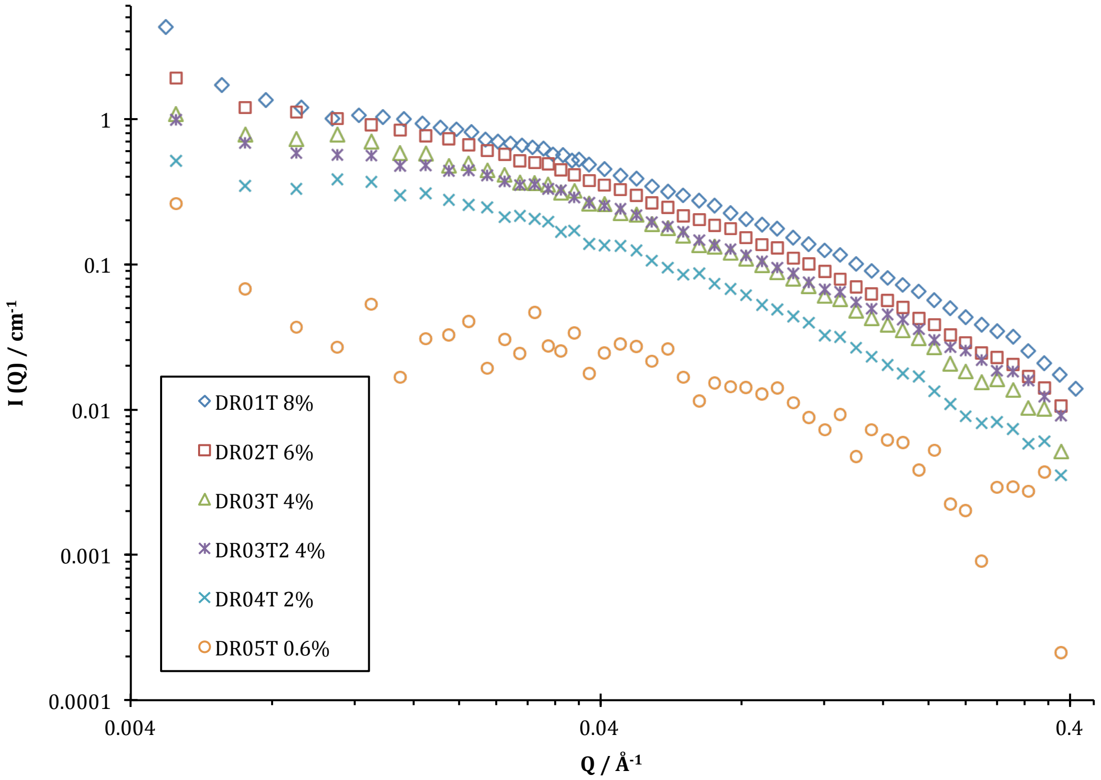

Figure S70. SANS profiles of [(*R*,*R*)-1][BAr$_F$] at different concentrations in THF-d$_8$, collected at 25 ˚C on SANS2D (ISIS)

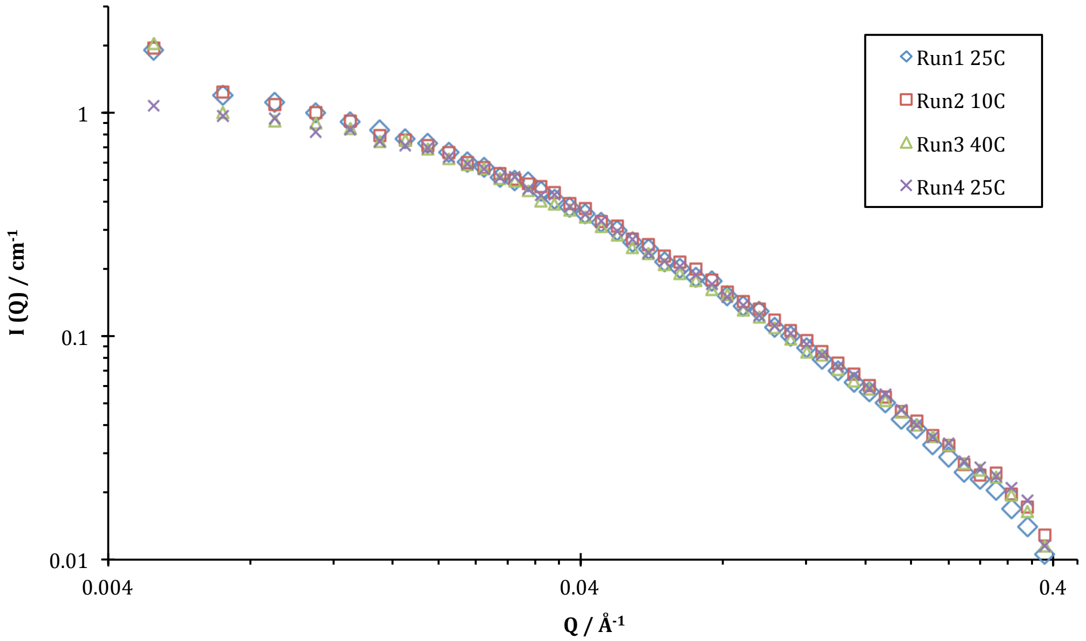

Figure S71. SANS profiles of 6.0% [(*R*,*R*)-1][BAr$_F$] in THF-d$_8$, collected at different temperatures on SANS2D (ISIS)

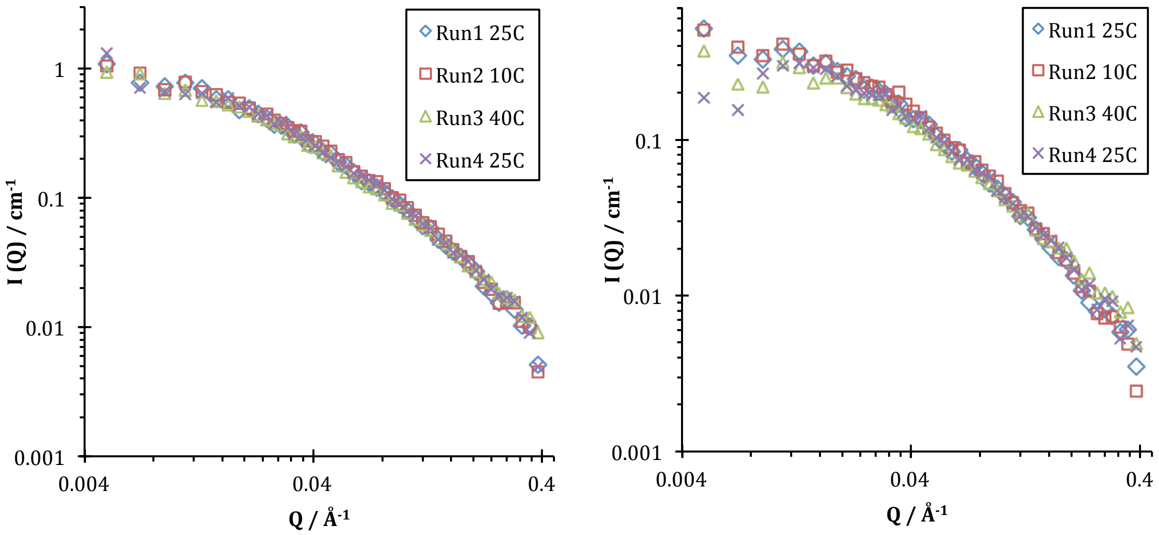

Figure S72. SANS profiles of 4.0% (left) and 2.0% (right) [(*R*,*R*)-1][BAr$_F$] in THF-d$_8$, collected at different temperatures on SANS2D (ISIS)

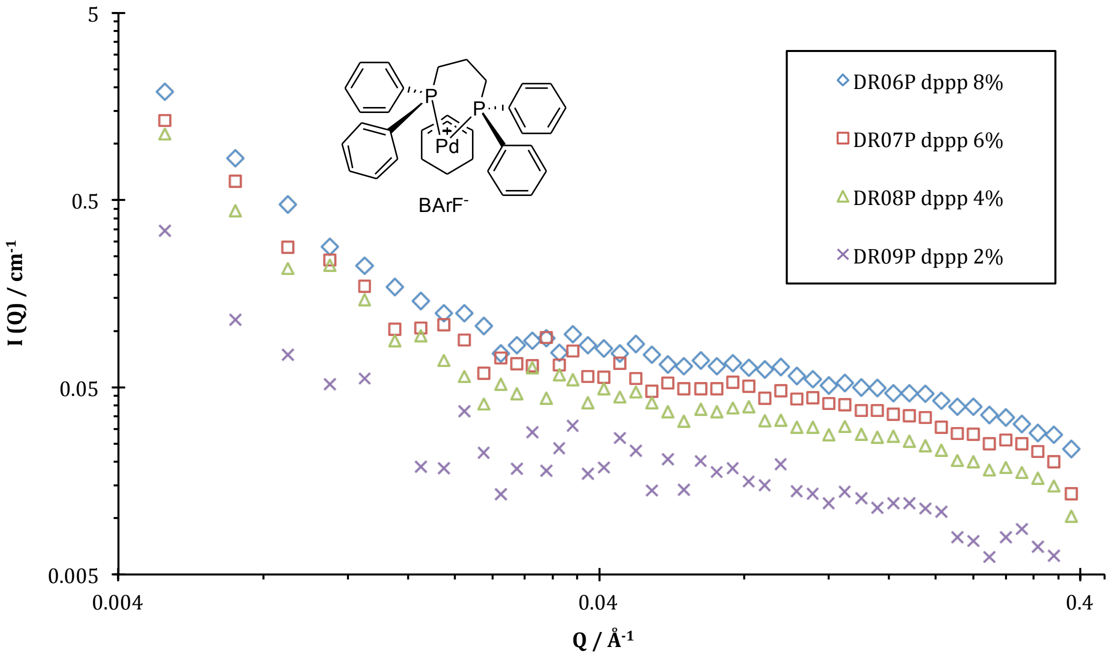

Figure S73. SANS profile of [18][BAr$_F$] in THF-D$_8$ at 25 ˚C

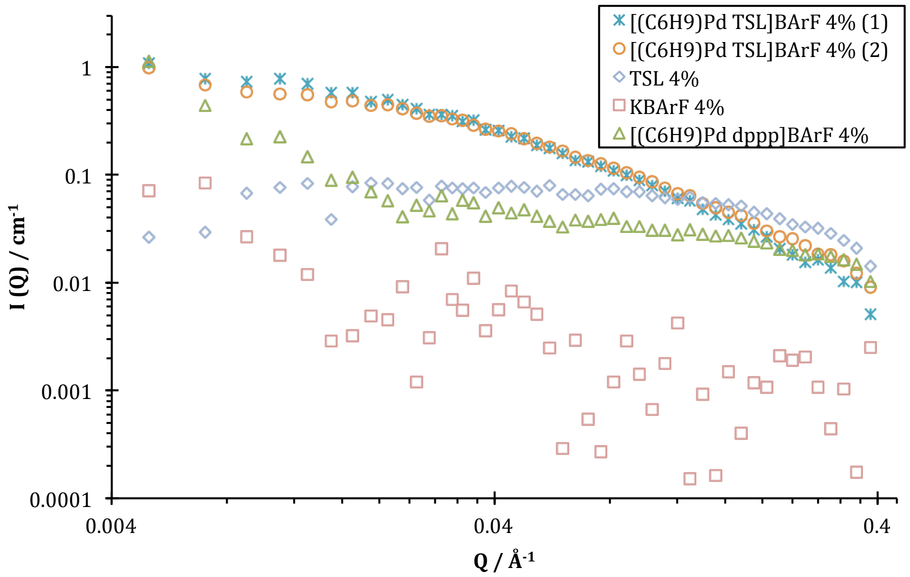

Figure S74. Comparison of the SANS profiles for 4% solutions of $(1)_n$, $KBAr_F$, TSL 2 and dppp complex [18][BAr_F] in THF-D_8 at 25 ˚C

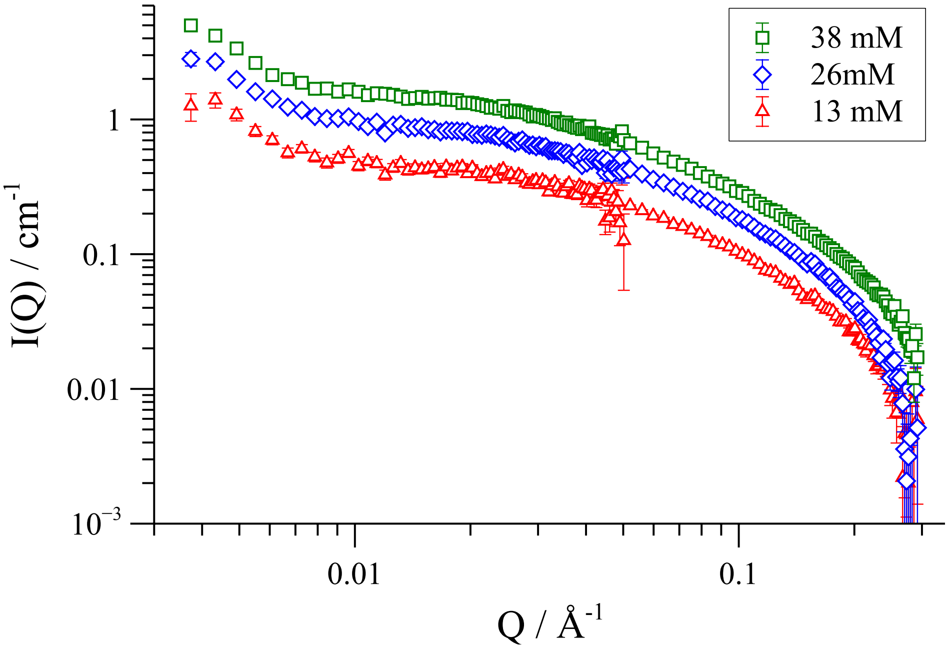

Figure S75. SANS profiles of [[D_{47}]-(*S*,*S*)-1][BAr_F] in THF at 25 ˚C, SANS2D, ISIS

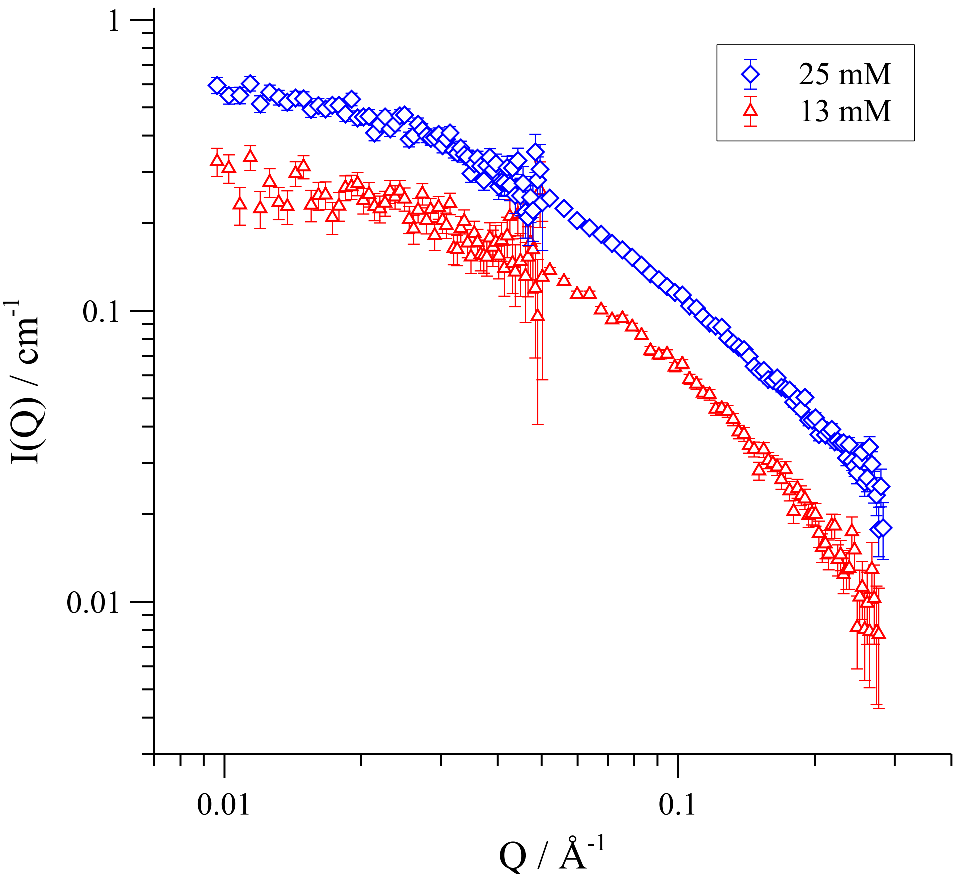

Figure S76. SANS of pseudo-enantiomers [[D_{47}]-(*S*,*S*)-1][BAr_F] + [[D_0]-(*R*,*R*)-1][BAr_F] in THF-H_8 at 25 °C, D11, ILL, France.

Table S9. Fitted size parameters of pseudo racemic deuterated, unlabelled and mixed samples of [1][BAr$_F$]; * data with extended high Q region from ILL D16; † instrument error: Radius ± 2Å; Length ± 20Å

	**[D_0-1] in D_8-THF**	**[D_{47}-1] in D_0-THF**	**[$D_{0/47}$-1] in D_8-THF**
**Length / Å**	~ 200†	~ 130	~ 150
**Radius (SasView) / Å**	~ 7*	~ 10	~ 10
**Radius (Guinier) / Å**	~ 8	~ 10	~ 9

Table S10. [1][BAr$_F$] fit parameters. SasView – cylinder model; FISH – Core Shell ROD with contr=0 and dr=0 parameters.

Scale Factor = Volume fraction × $(SLD_{Solvent} – SLD_{Substrate})^2 \times 10^{-24}$

**Parameter**	**10% ILL**	**8% ISIS**	**7.5% ILL**	**6% ISIS**	**5% ILL**	**4% ISIS**	**2.5% ILL**	**2% ISIS**
**Scale F. FISH**	3.0E-05	2.9E-05	2.5E-05	2.2E-05	1.8E-05	1.5E-05	9.8E-06	8.0E-06
**Scale F. Calc**	3.0E-05	2.4E-05	2.3E-05	1.8E-05	1.5E-05	1.2E-05	7.6E-06	6.1E-06
**Length FISH**	180	220	210	220	220	220	220	220
**Length SasV**	180	260	200	250	220	260	210	230
**Length - ANAL**	148	[157-186]	154					
**Radius FISH**	7.9	7.9	7.9	8.0	8.1	8.4	8.1	8.3
**Radius SasV**	8.1	8.3	8.6	8.6	8.8	9.0	9.3	9.5
**Radius - Guinier**	7.9	8	7.6					
**Radius - Porod**	9.5							

S.7 References

[1] Ishibashi, K.; Matsubara, S. *Chem. Lett.* **2007**, *36*, 724-725.

[2] Pouchet, C. J.; Behnke, J. *The Aldrich Library of 13C and 1H FT NMR Spectra*, 1st Ed., Aldrich Chemical Company Inc., **2003**, Vol. 1, p.p. 376C.

[3] Nishida, A.; Shirato, F.; Nakagawa, M. *Tetrahedron* **2000**, *11*, 3789-3805.

[4] Sosa-Rivadeneyra, M.; Muñoz-Muñiz, O.; Anaya de Parrodi, C.; Quintero, L.; Juaristi, E. *J. Org. Chem.* **2003**, *68*, 2369-2375.

[5] Mastranzo, V. M.; Santacruz, E.; Huelgas, G.; Paz, E.; Sosa-Rivadeneyra, M. V.; Bernès, S.; Juaristi, E.; Quintero, L.; Parrodi, C. A. de *Tetrahedron: Asymmetry* **2006**, *17*, 1663-1670.

[6] Watson, I. D. G.; Yudin, A. K. *J. Org. Chem.* **2003**, *68*, 5160-5167.

[7] Chandrasekhar, M.; Sekar, G.; Singh, V. K. *Tetrahedron Lett.* **2000**, *41*, 10079-10083.

[8] Fisher, D. M.; Fenton, R. R.; Aldrich-Wright, J. R. *Chem. Commun.* **2008**, 5613-5615.

[9] Yamashita, M.; Cuevas Vicario, J. V.; Hartwig, J. F. *J. Am. Chem. Soc.* **2003**, *125*, 16347-16360.

[10] Vachal, P.; Fletcher, J. M.; Hagmann, W. K. *Tetrahedron Lett.* **2007**, 48, 5761.

[11] Zhdankin, V. V.; Koposov, A. Y.; Litvinov, D. N.; Ferguson, M. J.; McDonald, R.; Luu, T.; Tykwinski, R. R. *J. Org. Chem.* **2005**, 70, 6484.

[12] Lin, L. L.; Hoyt, H. M.; van Halbeek, H.; Bergman, R. G.; Bertozzi, C. R. *J. Am. Chem. Soc.* **2005**, 127,
2686.

[13] Lloyd-Jones, G. C.; Stephen, S. C. *Chem. Eur. J.* **1998**, *4*, 2539-2549.

[14] Kim, Y. K.; Lee, S. J.; Ahn, K. H. *J. Org. Chem.,* **2000**, *65*, 7807-7813.

[15] Trost, B. M., Van Vranken, D. L., Bingel, C., *J. Am. Chem. Soc.* **1992**, 114, 9327.

[16] Imaizumi, S.; Matsuhisa, T.; Senda, Y. *J. Organomet. Chem.* **1985**, *280*, 441-448

[17] Trost, B. M.; Strege, P. E.; Weber, L.; Fullerton, T. J.; Dietsche, T. J. *J. Am. Chem. Soc.* **1978**, *100*, 3407–3415.

[18] Butts, C. P.; Filali, E.; Lloyd-Jones, G. C.; Norrby, P.-O.; Sale, D. A; Schramm, Y. *J. Am. Chem. Soc.* **2009**, *131*, 9945-9957.

[19] Pearson, A. J.; Hsu, S.-Y. *J. Org. Chem.*, **1986**, 51, 2505-2511.

[20] http://www.small-angle.ac.uk/small-angle/Software/FISH.htmL

[21] www.sasview.org v2.2. This work benefitted from SasView software,originally developed by the DANSE project under NSF award DMR-0520547

[22] Sears, V. F. *Neutron News*, **1992**, *3*, 26-37.

[23] Eastoe, J. *Surfactant Chemistry*, Wuhan University Press, **2005**, pp 153-173.

[24] Heenan, R. K. *FISH Data Analysis Program*; ISIS, UK, http://www.small-angle.ac.uk/small-angle/Software/FISH.html

[25] SasView, http://www.sasview.org/

[26] Hagelin, H.; Svensson, M.; Åkermark, B.; Norrby, P.-O. *Organometallics* **1999**, *18*, 4574–4583.

[27] Hagelin, H.; Åkermark, B.; Norrby, P.-O. *Organometallics* **1999**, *18*, 2884-2895.

[28] **Suite 2012**: MacroModel, version 9.9, Schrödinger, LLC, New York, NY, 2012.
